# Heart failure pharmacotherapy and cancer: pathways and pre-clinical/clinical evidence

**DOI:** 10.1093/eurheartj/ehae105

**Published:** 2024-03-05

**Authors:** Nabil V Sayour, Ágnes M Paál, Pietro Ameri, Wouter C Meijers, Giorgio Minotti, Ioanna Andreadou, Antonella Lombardo, Massimiliano Camilli, Heinz Drexel, Erik Lerkevang Grove, Gheorghe Andrei Dan, Andreea Ivanescu, Anne Grete Semb, Gianluigi Savarese, Dobromir Dobrev, Filippo Crea, Juan-Carlos Kaski, Rudolf A de Boer, Péter Ferdinandy, Zoltán V Varga

**Affiliations:** Department of Pharmacology and Pharmacotherapy, Semmelweis University, H-1085 Budapest, Üllői út 26, Hungary; HCEMM-SU Cardiometabolic Immunology Research Group, H-1089 Budapest, Nagyvárad tér 4, Hungary; MTA-SE Momentum Cardio-Oncology and Cardioimmunology Research Group, H-1089 Budapest, Nagyvárad tér 4, Hungary; Department of Pharmacology and Pharmacotherapy, Semmelweis University, H-1085 Budapest, Üllői út 26, Hungary; Cardiovascular Disease Unit, IRCCS Ospedale Policlinico San Martino, Italian IRCCS Cardiology Network, Genova, Italy; Department of Internal Medicine, University of Genova, Genova, Italy; Department of Cardiology, Thorax Center, Erasmus University Medical Center, Rotterdam, The Netherlands; University Campus Bio-Medico, Via Álvaro del Portillo, 21, 00128 Rome, Italy; Laboratory of Pharmacology, School of Pharmacy, National and Kapodistrian University of Athens, Athens, Greece; Department of Cardiovascular and Pulmonary Sciences, Catholic University of the Sacred Heart, Rome, Italy; Department of Cardiovascular Medicine, Fondazione Policlinico Universitario A. Gemelli IRCCS, Rome, Italy; Department of Cardiovascular and Pulmonary Sciences, Catholic University of the Sacred Heart, Rome, Italy; Department of Cardiovascular Medicine, Fondazione Policlinico Universitario A. Gemelli IRCCS, Rome, Italy; Vorarlberg Institute for Vascular Investigation & Treatment (VIVIT), Carinagasse 47, A-6800 Feldkirch, Austria; Department of Cardiology, Aarhus University Hospital, Aarhus, Denmark; Department of Clinical Medicine, Faculty of Health, Aarhus University, Aarhus, Denmark; Carol Davila University of Medicine and Pharmacy, Colentina University Hospital, Bucharest, Romania; Cardiology Department, Colentina Clinical Hospital, Bucharest, Romania; Carol Davila University of Medicine and Pharmacy, Colentina University Hospital, Bucharest, Romania; Cardiology Department, Colentina Clinical Hospital, Bucharest, Romania; Division of Research and Innovation, REMEDY-Centre for Treatment of Rheumatic and Musculoskeletal Diseases, Diakonhjemmet Hospital, Oslo, Norway; Division of Cardiology, Department of Medicine, Karolinska Institutet, Stockholm, Sweden; Heart and Vascular and Neuro Theme, Karolinska University Hospital, Stockholm, Sweden; Institute of Pharmacology, West German Heart and Vascular Center, University Duisburg-Essen, Essen, Germany; Department of Medicine and Research Center, Montreal Heart Institute and Université de Montréal, Montréal, QC, Canada; Department of Integrative Physiology, Baylor College of Medicine, Houston, TX, USA; Department of Cardiovascular and Pulmonary Sciences, Catholic University of the Sacred Heart, Rome, Italy; Department of Cardiovascular Medicine, Fondazione Policlinico Universitario A. Gemelli IRCCS, Rome, Italy; Molecular and Clinical Sciences Research Institute, St. George’s University of London, London, United Kingdom; Department of Cardiology, Thorax Center, Erasmus University Medical Center, Rotterdam, The Netherlands; Department of Pharmacology and Pharmacotherapy, Semmelweis University, H-1085 Budapest, Üllői út 26, Hungary; Pharmahungary Group, Szeged, Hungary; MTA-SE System Pharmacology Research Group, Department of Pharmacology and Pharmacotherapy, Semmelweis University, Budapest, Hungary; Department of Pharmacology and Pharmacotherapy, Semmelweis University, H-1085 Budapest, Üllői út 26, Hungary; HCEMM-SU Cardiometabolic Immunology Research Group, H-1089 Budapest, Nagyvárad tér 4, Hungary; MTA-SE Momentum Cardio-Oncology and Cardioimmunology Research Group, H-1089 Budapest, Nagyvárad tér 4, Hungary

**Keywords:** Cardio-oncology, Heart failure, Cancer, Beta-blocker, Mineralocorticoid Receptor antagonist, Sodium-glucose cotransporter 2 inhibitor, Angiotensin receptor blocker, Angiotensin-converting enzyme inhibitor, Angiotensin receptor-neprilysin inhibitor

## Abstract

Heart failure (HF) patients have a significantly higher risk of new-onset cancer and cancer-associated mortality, compared to subjects free of HF. While both the prevention and treatment of new-onset HF in patients with cancer have been investigated extensively, less is known about the prevention and treatment of new-onset cancer in patients with HF, and whether and how guideline-directed medical therapy (GDMT) for HF should be modified when cancer is diagnosed in HF patients. The purpose of this review is to elaborate and discuss the effects of pillar HF pharmacotherapies, as well as digoxin and diuretics on cancer, and to identify areas for further research and novel therapeutic strategies. To this end, in this review, (i) proposed effects and mechanisms of action of guideline-directed HF drugs on cancer derived from pre-clinical data will be described, (ii) the evidence from both observational studies and randomized controlled trials on the effects of guideline-directed medical therapy on cancer incidence and cancer-related outcomes, as synthetized by meta-analyses will be reviewed, and (iii) considerations for future pre-clinical and clinical investigations will be provided.

## Graphical Abstract

**Graphical abstract:**
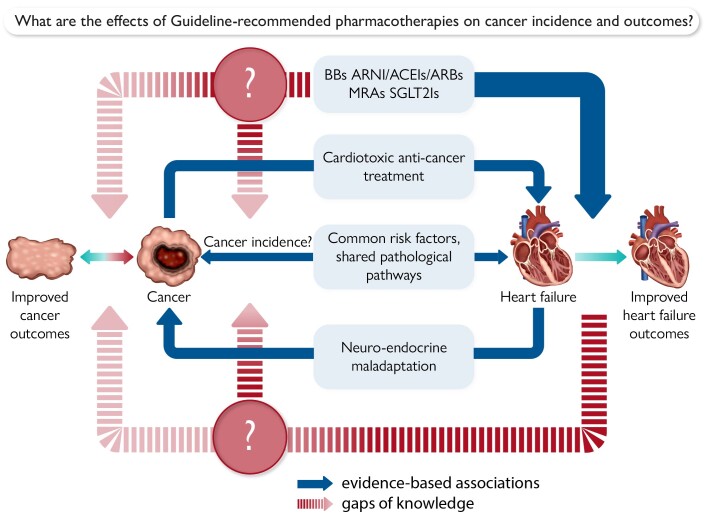
The risk factors and pathophysiological pathways of both heart failure and cancer are common. The discipline of cardio-oncology investigates how heart failure and cancer progression are connected: on one hand, the cardiac effects of anti-cancer medications or cancer-derived metabolic byproducts are investigated, whereas on the other hand, the possible effects of heart failure on cancer progression are examined, such as those mediated by maladaptive neuroendocrine activation and factors secreted from the failing heart. Nevertheless, there is a lack of systematic knowledge on how heart failure pharmacotherapies affect new-onset cancer incidence or prevalent cancer outcomes, and whether these effects are mediated through the improvement in cardiac functions. ACEI, angiotensin-converting enzyme inhibitor; ARB, angiotensin receptor blocker; BB, beta-blocker; MRA, mineralocorticoid receptor antagonist; SGLT2I, sodium-glucose cotransporter 2 inhibitor.

## Introduction

Heart failure (HF) and cancer are leading causes of mortality worldwide.^1–6^ Although HF and cancer are conventionally viewed as two separate disease entities, an implicit bidirectional relationship between them has been identified by recent studies,^7^ as (i) major risk factors and mechanistic pathways overlap in HF and cancer,^8–11^ (ii) in patients with prevalent cancer, cardiovascular diseases are the leading causes of non-cancer mortality,^12–14^ (iii) several cancer pharmacotherapies exert cardiotoxic effects,^15–18^ and (iv) in patients with prevalent HF, the majority of observational evidence reported increased cancer incidence and worse cancer outcomes compared with subjects free of HF, irrespective of patients’ age, HF etiology, and cancer type.^19–25^ However, an epidemiological study on men with self-reported HF reported no such associations,^26^ moreover, a Danish nationwide study reported a significant decrease in cancer incidence in patients with prevalent HF after adjusting for multiple variables including co-morbidities and medications.^27^ Interestingly, despite major improvements in HF therapies, cancer incidence in HF patients has remained unchanged for the past 20 years, underscoring the importance of cancer in the setting of HF.^28,29^

Guideline recommendations exist regarding prevention, screening, monitoring, and treatment of new-onset HF in patients receiving cancer therapies.^3^ However, no recommendations are available that define if and how HF treatment should be modified (i) to prevent cancer incidence in HF patients, or (ii) when cancer is diagnosed during the course of HF, and no ongoing clinical studies are available addressing these questions. Indeed, based on a systematic search on ClinicalTrials.gov, we found that all of the ongoing clinical studies of cardio-oncology are related to prevention or treatment of cancer therapy-related cardiotoxicity (see Supplementary data online, *Table S1*).

The purpose of this review is to provide (i) an overview of the effects and proposed mechanisms of action of GDMT of HF on cancer based on pre-clinical data, and (ii) a balanced interpretation of findings reported in clinical meta-analyses investigating the effects of HF GDMT on cancer incidence and outcomes. Moreover, gaps in knowledge and areas of future pre-clinical and clinical research will also be highlighted.

In this narrative review, we collected evidence from *in vivo* (see Supplementary data online, *Table S2*) and *in vitro* (see Supplementary data online, *Table S3*) pre-clinical studies, with a special emphasis on drug-type, dosing, cancer type, and endpoints. We also collected information from meta-analyses of clinical studies investigating the effect of HF GDMT on cancer incidence in cancer-free patients, or other cancer-related outcomes (e.g. cancer-specific or recurrence-free survival) in patients with pre-existing cancer at baseline (see Supplementary data online, *Table S4*).

## Effects of beta-blockers on cancer

**Figure 1:**
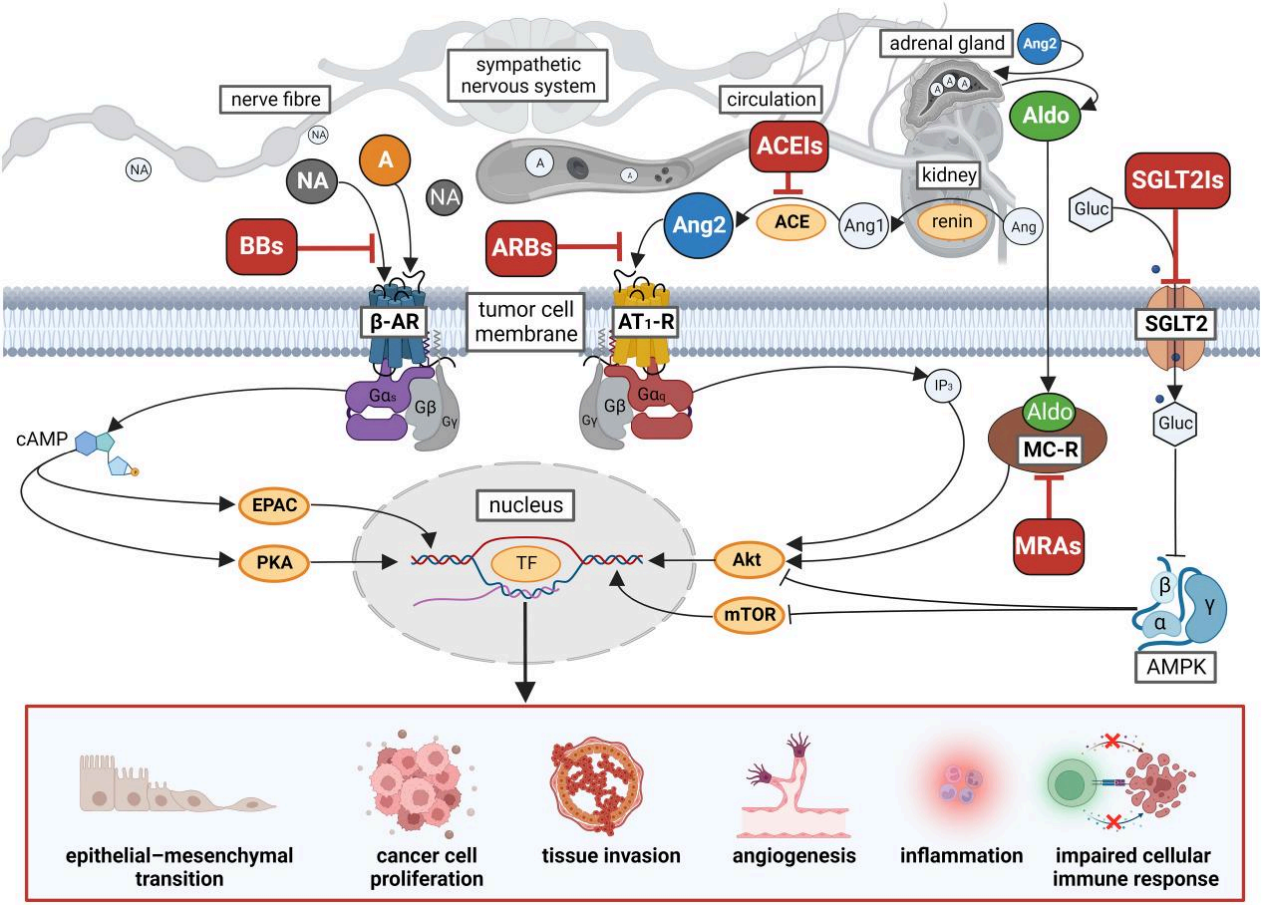
Suggested mechanism of action of the different heart failure pharmacotherapies on cancer cells. A: Adrenaline, NA: noradrenaline, Ang: angiotensinogen, Ang1: angiotensin-I, Ang2: angiotensin-II, ACE: angiotensin-converting enzyme, Aldo: aldosterone, ARB: angiotensin receptor blocker, ACEI: angiotensin-converting enzyme inhibitor, BB: β-blocker, SGLT2I: sodium-glucose cotransporter 2 inhibitor, ß-AR: ß-adrenergic receptor, AT1-R: angiotensin II receptor type 1, SGLT2: sodium-glucose cotransporter 2, MC-R: mineralocorticoid receptor, MRA: mineralocorticoid receptor antagonist, cAMP: cyclic adenosine-monophosphate, EPAC: exchange protein directly activated by cAMP, PKA: protein-kinase A, IP3: inositol-triphosphate, Akt: protein-kinase B, mTOR: mammalian target of rapamycin, Gluc: glucose, TF: transcription factors. Figure created with BioRender.com.

Beta-adrenoceptor signalling has been suggested to play a contributory role in cancer biology, as it modulates cancer progression mainly via the activation of protein kinase A and the exchange protein activated by adenylyl cyclase (*Figure 1*).^30^ Catecholamines regulate beta-adrenoceptors on cancer cells, stromal cells, and tumour-associated macrophages,^31^ resulting in a procarcinogenic microenvironment. Accordingly, beta-blockers (BBs) may have a potential to decrease cancer incidence or improve cancer outcomes.

### Pre-clinical studies assessing the effects of beta-blockers on cancer

Effects of BBs on cancer have been extensively investigated in pre-clinical studies, almost unanimously demonstrating potent anti-cancer effects both *in vivo* (see Supplementary data online, *Table S2*) and *in vitro* (see Supplementary data online, *Table S3*). Most *in vivo* studies tested the non-selective BB propranolol. Propranolol exerted significant anti-cancer effects *in vivo* by inhibiting tumour growth,^32–35^ reducing metastases,^36,37^ influencing tumour immuno-microenvironment,^38^ and by repressing angiogenesis.^39^ In contrast, some studies showed that propranolol did not have anti-cancer effects *per se*,^40^ as it could only enhance the effects of other anti-cancer therapies.^38,41–44^ Among *in vivo* studies investigating the anti-cancer effects of non-selective BBs other than propranolol, carvedilol was the most commonly used agent. Most of these studies demonstrated significant anti-cancer effects of carvedilol when used alone in a variety of cancer types.^45–52^ Other BBs, such as the beta1-selective metoprolol^41^ and nebivolol,^53,54^ as well as the non-selective labetalol^33^ were also shown to have either anti-cancer effects *per se*, or enhance the anti-cancer effects of other drugs.

Anti-cancer effects of various BBs on several cancer types have been assessed by *in vitro* studies, resulting in variable results. For instance, anti-cancer efficacy of propranolol or beta2-adrenoceptor blockade was reported to be higher compared to beta1-selective BBs by the majority of studies.^34,55–58^ Conversely, a study on non-small cell lung cancer cell lines demonstrated no correlation between beta-adrenoceptor selectivity and anti-cancer efficacy of BBs, as both propranolol and the beta1-selective betaxolol significantly decreased colony formation.^59^ Of note, metoprolol, the only beta1-selective BB approved for HF that was investigated in this study, was ineffective in such settings. Moreover, the superiority of beta1-selective BBs over propranolol has also been demonstrated *in vitro*,^60–62^ further complicating the picture.

Of note, BB use emerged not only in prevention of cancer development *per se*, but also in prevention of cancer therapy-related cardiotoxicity, as beta-adrenoceptor signalling was shown to share an intricate conundrum with human epidermal growth factor receptor type 2 (ERBB2) in the cardiovascular system, and also in breast cancer.^63,64^ As a result, the BB carvedilol was shown to prevent ERBB2-blockade-induced cardiotoxicity.^65^

### Clinical studies assessing the effects of beta-blockers on cancer

**Figure 2:**
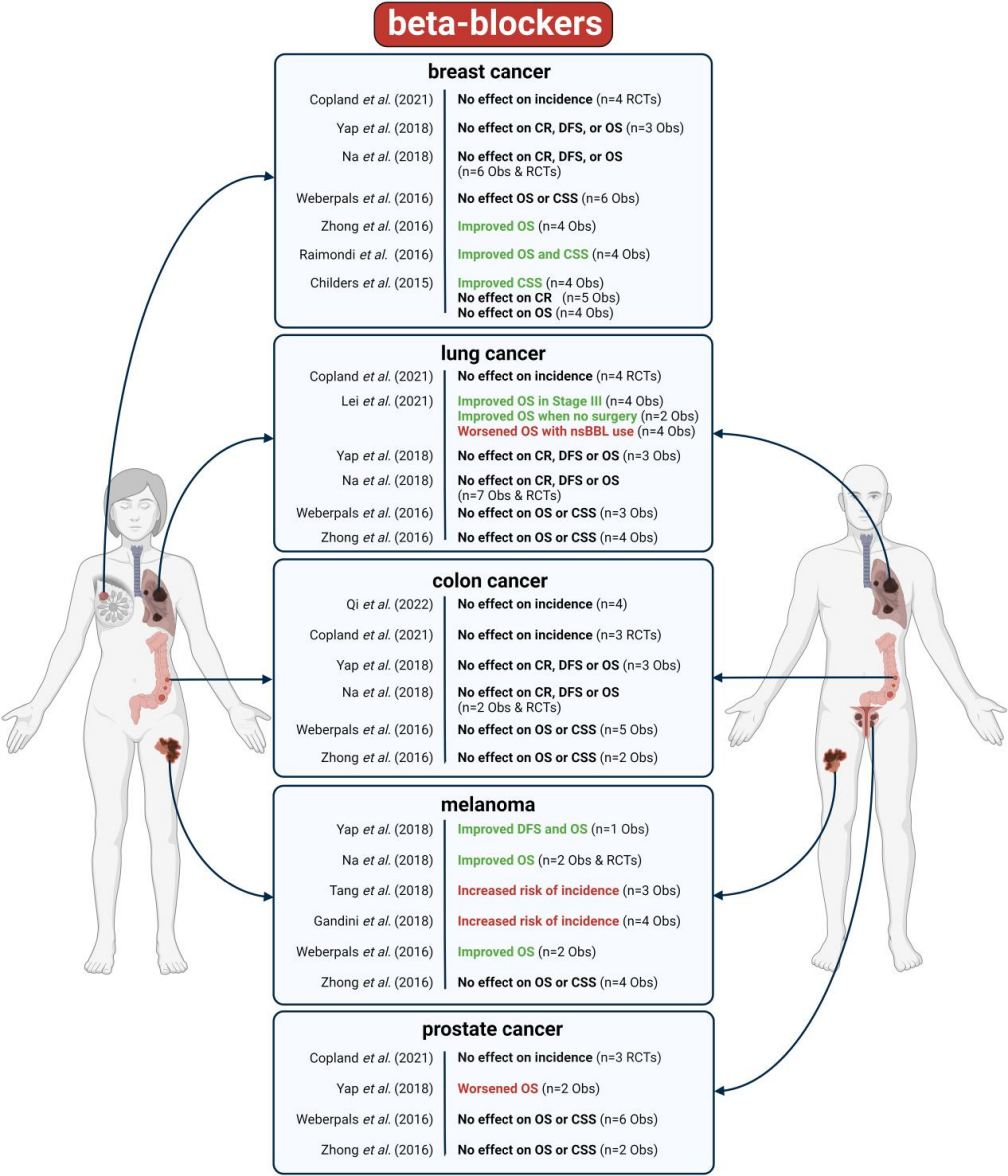
Meta-analyses of clinical studies on the effect of beta-blockers on cancer incidence or outcomes. The number of studies used for overall effect size estimation is marked (n). OS: overall survival, CSS: cancer specific survival, CR: cancer recurrence, DFS: disease-free survival, Obs: observational studies, RCTs: randomized controlled trials. The meta-analyses are referenced in the text. Figure created with BioRender.com.

Intriguingly, contrary to pre-clinical studies, meta-analyses on clinical observational studies or randomized controlled trials (RCTs) show disparate results regarding effects of BBs on cancer, both in cancer-free patients, and in cancer patients (*Figure 2*, see Supplementary data online, *Table S4*).

#### Effects of beta-blockers on risk of cancer in cancer-free patients

In a meta-analysis on nine RCTs, BB use was associated with a non-significant trend towards lower overall risk of any cancer type.^66^ Likewise, a network meta-analysis on 70 RCTs showed that the use of BBs was not associated with any change in risk of any cancer type, or cancer mortality.^67^ In other meta-analyses, BB use was associated with mixed effects on cancer incidence, varied by cancer types. For instance, two meta-analyses showed no association between BB use and risk of new-onset breast, lung, colon, or prostate cancer.^68,69^ Other meta-analyses showed that BB use was associated with a significantly increased risk of melanoma,^70,71^ but of note, these meta-analyses included the same primary studies. In addition, a meta-analysis resulted in a significantly increased risk of kidney or bladder cancer in BB users,^72^ while another meta-analysis demonstrated a significantly reduced risk for hepatocellular carcinoma in patients with liver cirrhosis using non-selective BBs.^73^ Of interest, none of these meta-analyses used HF patients, or HF as an indication for BB use exclusively, according to their study eligibility criteria.

#### Effects of beta-blockers on cancer outcomes in patients with prevalent cancer

Meta-analyses on breast cancer showed that BB use was associated with either no effect,^74–76^ or with improved cancer outcomes^77–79^ compared to non-users, even when BBs were started after diagnosis of malignancy.^79^ Most meta-analyses on lung cancer show no associations of BB use with cancer outcomes.^74–76,79^ Still, one meta-analysis reported that (i) non-selective BB use was associated with significantly worse overall survival of lung cancer patients, and that (ii) BB use (not stratified by selectivity) was associated with improved overall survival in stage III patients and in those without surgical cancer treatment.^80^ With respect to colorectal cancer, no association has been found between BB use and cancer outcomes.^74–76,79^ Regarding malignant melanoma, repeated analyses on the same cohorts hinted towards improved overall survival in patients using BB.^74–76^ Conversely, another meta-analysis that included additional cohort studies showed no association of BB use with beneficial cancer outcomes in patients with malignant melanoma.^79^

In conclusion, although BBs have been shown to exert significant anti-cancer effects in pre-clinical studies, meta-analyses of clinical studies show inconsistent and sometimes conflicting results regarding the associations of BB use with cancer incidence and outcomes, independently from cancer site and outcome measures. Moreover, there is no consensus on how beta-adrenoceptor selectivity influences the effect of BBs on cancer neither in pre-clinical nor in clinical studies. It should also be stressed that BBs have no proven effects in HF with preserved ejection fraction, and only minor cardioprotective effects in cancer patients receiving chemotherapy.^81^ Nevertheless, as the activation of sympathetic nervous system on cancer outcomes has been well-established in both pre-clinical studies and the clinical settings,^82^ there is a strong rationale to further investigate the sympathetic nervous system–cancer relationships.

## Effects of renin-angiotensin-aldosterone system inhibitors on cancer

Many studies have shown that dysregulation of the renin-angiotensin-aldosterone system (RAAS) may promote cancer, mainly driven by the AT1R-Akt axis (*Figure 1*).^83^ The idea for investigating the effects of RAAS blockade on cancer has first emerged by the retrospective analysis of Lever *et al.*, showing that patients using angiotensin-converting enzyme inhibitors (ACEIs) had a reduced risk for developing cancer, and also suggested the need to assess the effects of angiotensin receptor blockers (ARBs) on cancer as well.^84^ This seminal study gave rise to pre-clinical and clinical studies testing the hypothesis that RAAS blockade entails anti-cancer effects, as well as to investigations demonstrating that components of RAAS are expressed in various human cancers and in their microenvironment,^83^ which are associated with worse outcomes.^85,86^

### Pre-clinical studies assessing the effects of renin-angiotensin-aldosterone system inhibitors on cancer

The anti-cancer effects of blocking the RAAS with ACEIs were tested in numerous pre-clinical *in vivo* studies, mostly demonstrating benefits, that could be exerted either alone or in combination with other anti-cancer therapies (see Supplementary data online, *Table S2*).^87–91^ Nevertheless, contradictory findings were reported in an early study by Wysocki and colleagues, where captopril did not exert significant anti-cancer effects, but was associated with increased mortality in immunocompetent mouse models of renal cancer.^92^ However, in a more recent study using a similar immunocompetent cancer model and the same cancer cell line, captopril significantly reduced primary tumour weight and lung metastases. Of note, captopril treatment was started 2 days prior to tumour inoculation, and in a lower dose.^93^ The anti-cancer effects of RAAS blockade by ACEIs are further supported by *in vitro* studies, showing a reduction in cell proliferation, migration, and invasion.^94–99^ Nevertheless, in contrast to these *in vivo* studies, several *in vitro* studies reported no direct anti-cancer effects of ACEIs,^91,100–102^ or no synergism with other anti-cancer agents (see Supplementary data online, *Table S3*).^103^ Whether findings of these pre-clinical studies are a class effect not known, as captopril was assessed almost exclusively. Thus, a comprehensive, systematic research strategy to assess the effects of different types of ACEIs is lacking.

ARBs inhibit the AT1R, the key target of angiotensin II, which is the major effector peptide of the RAAS. In tumour-bearing mice, ARBs exert significant anti-cancer effects by reducing tumour growth and/or fibrosis,^104–107^ metastases,^108,109^ tumour neo-angiogenesis,^110–112^ and influencing tumour immuno-microenvironment (see Supplementary data online, *Table S2*).^108,113^ In a seminal study by Rhodes *et al.*, AT1R is overexpressed in 10%–20% of breast cancer cases across multiple independent patient cohorts. The study indicated that marked AT1R-overexpression defines a subpopulation of estrogen receptor-positive, ERBB2-negative breast cancer that may benefit from targeted therapy with ARBs, most particularly losartan. These findings were obtained in both *in vitro* and *in vivo* models AT1R-overexpressing breast cancer, but not in AT1R^low^ cell line.^114^ Nevertheless, contradictory results were obtained by a number of *in vitro* and *in vivo* studies demonstrating no or less anti-cancer effects of ARBs, mostly using losartan or irbesartan^99,115–127^ (see Supplementary data online, *Table S3*).

Mineralocorticoid receptor antagonists (MRAs) represent a third pillar part of HF GDMT. Only a handful of pre-clinical studies investigated effects of MRAs on cancer currently. Leung and colleagues demonstrated that spironolactone decreased the number of intestinal polyps in APJ^min^ mice (a mouse model of spontaneous intestinal adenoma formation), and inhibited metastases in colorectal carcinoma-implantation studies by pathways that are independent of the mineralocorticoid receptor.^128^ Other *in vivo* studies also demonstrated a significant anti-cancer effect of MRAs by reducing tumour volume,^129–131^ and/or by inhibiting metastatic spread^132^ (see Supplementary data online, *Table S2*). Accordingly, the majority of *in vitro* studies also show an overall anti-cancer effect of MRAs either given alone or in combination with other therapeutics (see Supplementary data online, *Table S3*).^129,133–137^ Of note, Gold and colleagues reported differences in anti-cancer efficacy of different MRAs, showing superiority of spironolactone over eplerenone,^130^ a differential effect that needs further elucidation regarding mechanism. Conversely, lack of anti-cancer effects for spironolactone was also found on liver,^131^ and pituitary cancer cell lines.^138^ Moreover, Aldaz and colleagues demonstrated that spironolactone (either alone, or in combination with dexamethasone) protected the glioblastoma cells against radiation-induced damage.^139^

### Clinical studies assessing the effects of renin-angiotensin-aldosterone system inhibitors on cancer

**Figure 3:**
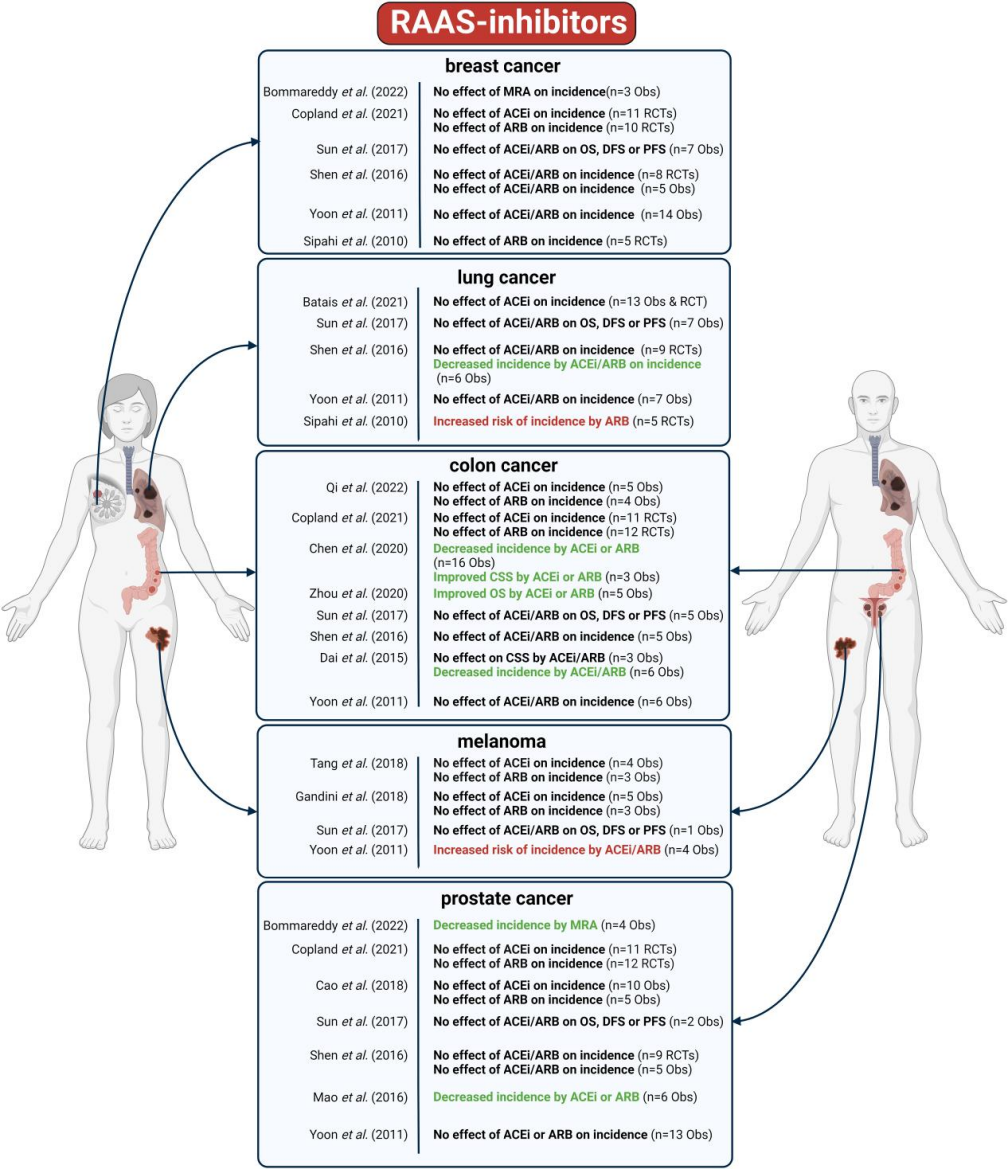
Meta-analyses of clinical studies on the effect of RAAS inhibitors (angiotensin receptor blockers, angiotensin-converting enzyme inhibitors, or mineralocorticoid receptor antagonists) on cancer incidence or outcomes. The number of studies used for overall effect size estimation is marked (n). OS: overall survival, CSS: cancer specific survival, CR: cancer recurrence, DFS: disease-free survival, Obs: observational studies, RCTs: randomized controlled trials. The meta-analyses are referenced in the text. Figure created with BioRender.com.

The putative effects of RAAS blockade on malignancy in HF patients were investigated in a seminal meta-analysis by Sipahi and colleagues, in which only RCTs of ARBs were analyzed (*Figure 3*, see Supplementary data online, *Table S4*). Here, a significant association between the use of ARBs and overall cancer risk was reported, mostly attributed to new-onset lung cancer.^140^ These results raised doubts about the reliability of this meta-analysis, as adjudication of cancer diagnoses was not uniform among the included studies.^141^

#### Effects of renin-angiotensin-aldosterone system inhibitors on risk of cancer in cancer-free patients

Other meta-analyses of RCTs reported that ARB or ACEI usage was not associated with cancer risk compared to placebo.^67,68,142–145^ This lack of association between RAAS inhibitor (RAASI) use and incident cancer has also been suggested by meta-analyses of cohort studies across multiple cancer types.^69–71,146,147^ However, meta-analyses of non-randomized investigations demonstrated a significantly decreased incidence of esophagus,^148^ colorectal,^149^ prostate,^150^ and lung cancer,^143^ but an increased risk for renal cancer^72,148^ and melanoma^148^ amongst users of ACEI/ARB compared to non-users. Although a large number of meta-analyses have been conducted to investigate the association between ACEI/ARB use and cancer incidence, only a single recent meta-analysis by Bommareddy and colleagues assessed the effect of spironolactone on cancer occurrence.^151^ This meta-analysis synthesized data from observational studies, showing a significantly decreased risk for prostate cancer, but no effect on other cancer types. Similar to BBs, effects of RAASIs on cancer incidence were mostly assessed in hypertensive, but not in HF populations by the meta-analyses.

#### Effects of renin-angiotensin-aldosterone system inhibitors on cancer outcomes in patients with prevalent cancer

In contrast to the disparate effects of RAASIs on cancer incidence, meta-analyses of observational studies demonstrated significantly improved cancer outcomes in patients with digestive system malignancies,^152–154^ renal cancer,^153,155^ or all-cause cancer^156^ in users of ACEIs or ARBs. Nevertheless, a meta-analysis on RCTs showed the neutral effect of RAASIs in cancer patients irrespective of the cancer type.^157^

In conclusion, the effect of RAASIs on new-onset cancer risk is conflicting in the current clinical data, varying mostly by cancer types, and also, by primary study design (i.e. RCT or observational). However, in patients with prevalent cancer, the majority of meta-analyses either show safety, or even improved cancer-related outcomes when RAASIs are used, compared to non-users. Nevertheless, a major factor that complicates the interpretation of these results is the confounding by indication, i.e. most of the clinical data are derived from patients with hypertension, and not with HF, urging for further evidence in the HF populations as well.

## Effects of angiotensin receptor-neprilysin inhibitor on cancer

There is a general lack of pre-clinical and clinical evidence regarding the effect of angiotensin receptor-neprilysin inhibitor (ARNI, i.e. sacubitril/valsartan) on cancer, which is another part for HF GDMT,^1^ a drug that enhances the beneficial cardiovascular effects of endogenous natriuretic peptides (NP)^158,159^ Of note, in the landmark RCT leading to approval of ARNI for treatment of HF with reduced ejection fraction, proportion of cancer deaths was comparable in the ARNI and ACEI arms.^160^ Moreover, in a recent cohort study on patients with HF with mildly reduced ejection fraction, ARNI/ACEI/ARB use significantly increased cancer incidence in the primary outcomes within 3 years, although this association was not significant by falsification analysis.^161^

NPs have been shown to inhibit tumour growth in several *in vitro* and *in vivo* studies,^162–165^ nevertheless, these associations should be interpreted with caution, as some malignant cells are also able to produce NPs, questioning generalizability of tumour-inhibitory effects of NPs.^166,167^ In addition, it is noteworthy that in principle neprilysin inhibition also increases the availability of factors other than NPs that might influence cancer cell biology.^168^ The effects of these substrates should also be considered in future investigations addressing the effects of ARNI on cancer.

## Effects of sodium-glucose cotransporter 2 inhibitors on cancer

As glucose is a major substrate required for cancer cell survival and growth, Scafoglio and colleagues hypothesized that the metabolism-shifting effect of sodium-glucose cotransporter 2 inhibitors (SGLT2Is) might be protective against malignancy as well.^169^ In this seminal investigation, functional expression of SGLT2 on human pancreatic and prostate cancers was demonstrated. In addition, this was the first study providing evidence on SGLT2Is blocking glucose uptake and reducing tumour growth in a xenograft model of pancreatic cancer, which led to the conduction of subsequent pre-clinical studies investigating the anti-cancer effects of SGLT2Is.^170^

### Pre-clinical studies assessing the effects of sodium-glucose cotransporter 2 inhibitors on cancer

Glucose uptake/metabolism-dependent anti-cancer mechanisms of SGLT2Is have been further demonstrated in cancer-bearing mice, which was attributed to activation of adenosine monophosphate-activated protein kinase (AMPK), and thus, to the inhibition of mTOR^171–173^ (*Figure 1,*  Supplementary data online, *Table S2*). Nevertheless, Kaji *et al.* reported the anti-cancer effect of SGLT2 inhibition to be exerted independently of the systemic glycemic status, although cellular glucose-uptake was not assessed here.^174^ Other mechanistic pathways on the anti-cancer effects of SGLT2 blockade were suggested by other studies, showing that SGLT2 inhibition (i) decreases pro-carcinogenic inflammation,^175^ (ii) activates AMPK, which leads to inactivation of the protooncogene sonic hedgehog,^176^ (iii) suppresses cancer progression by inhibiting the Hippo signalling pathway through downregulating YAP1 expression.^177^ In contrast, however, Korfhage and colleagues reported an increased intestinal adenoma burden in female, but not in male APC^min^ mice, when treated with canagliflozin,^178^ a result that needs further mechanistic elucidation.

Several *in vitro* studies also indicated that anti-cancer mechanisms of SGLT2Is are mainly attributed to the induction of AMPK,^179^ which subsequently leads to the inhibition of the Akt/mTOR pathway (see Supplementary data online, *Table S3*).^180^ In addition, analyses of metabolomics in SGLT2I-treated cancer cell lines showed that besides the glucose-dependent mechanisms, alteration of other metabolic pathways (e.g. fatty acid metabolism) also contributes to the decrease in cancer cell proliferation.^181,182^ Other *in vitro* studies reported that anti-proliferative effects of SGLT2Is are exerted by a significant repression of DNA synthesis,^183^ subsequent cell cycle arrest,^184^ and by blocking aberrant activation of ß-catenin.^185^ In the latter study, dapagliflozin and empagliflozin (the two SGLT2Is that are currently recommended in HF) exerted non-significant effects, questioning the presence of a class effect.

Of note, in addition to tumour growth studies, SGLT2Is were also investigated in cancer therapy-related toxicity studies. For instance, dapagliflozin and empagliflozin were shown to revert ponatinib-induced endothelial cell senescence and dysfunction.^186^

### Clinical studies assessing the effects of sodium-glucose cotransporter 2 inhibitors on cancer

**Figure 4:**
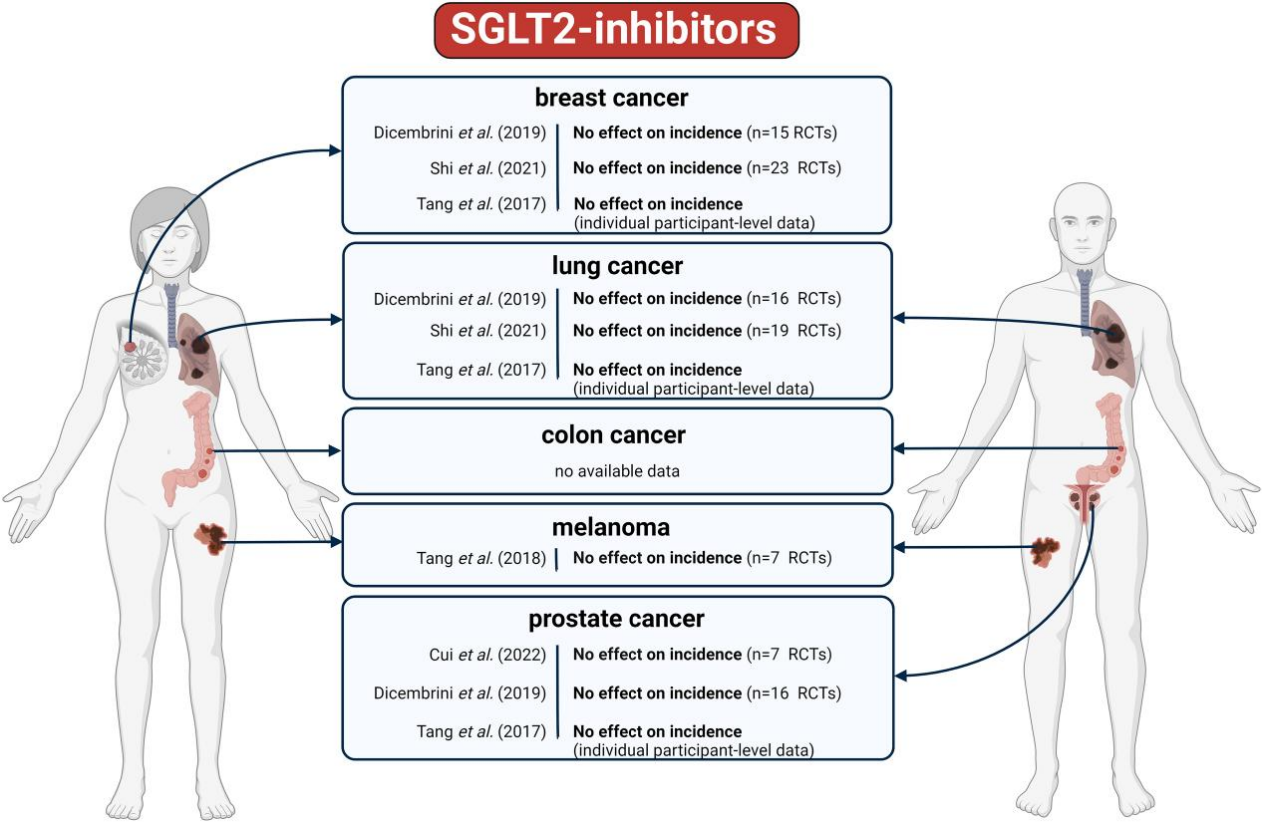
Meta-analyses of clinical studies on the effect of sodium-glucose cotransporter 2 inhibitors on cancer incidence or outcomes. The number of studies used for overall effect size estimation is marked (n). OS: overall survival, CSS: cancer-specific survival, CR: cancer recurrence, DFS: disease-free survival, Obs: observational studies, RCTs: randomized controlled trials. The meta-analyses are referenced in the text. Figure created with BioRender.com.

During the safety trials of SGLT2Is in diabetic patients, no significant increase in overall cancer events was observed. Nevertheless, a nominal increase in bladder cancer incidence in men, and breast cancer incidence in women were noted in the SGLT2I-treated arm.^187^ These observations have led to systematic investigations of the association between SGLT2I use and cancer, showing inconsistent results (see Supplementary data online, *Table S4* and *Figure 4*).

For instance, a recent meta-analysis of hyperglycaemic patients reported an overall reduced risk of cancer associated with SGLT2I use, and most particularly with dapagliflozin and ertugliflozin vs. placebo.^188^ Of note, in this meta-analysis, two trials with large sample sizes may have shifted the overall effect size towards benefit by SGLT2Is, whereas the majority of the included studies had large confidence intervals (i.e. small sample sizes) with non-significant effects. Surprisingly, an earlier meta-analysis showed no association of SGLT2I with malignancy, however (i) dapagliflozin significantly increased risk of overall cancer compared to other antidiabetic drugs, and (ii) empagliflozin nominally increased the risk of overall cancer compared to placebo in patients with type 2 diabetes mellitus (T2DM).^189^ Another meta-analysis on T2DM patients showed that risk of overall cancer in obese patients was significantly increased in association with SGLT2I use. This meta-analysis also showed a tendency towards increased risk of cancer in studies with a follow-up period of >52 months. Moreover, risk of bladder cancer also significantly increased, mainly associated with the use of empagliflozin.^190^ In contrast, Dicembrini and colleagues reported a significant risk reduction in bladder cancer associated with dapagliflozin use, although this result was derived from four RCTs, one of which might have been overweighed, thus, dominating the overall effect size.^191^

Meta-analyses investigating effects of SGLT2Is on outcomes by cancer types show no significant change in the risk of breast cancer,^189–191^ lung cancer,^189–191^ prostate cancer,^190–192^ or melanoma,^193^ with the latter cancer showing a tendency to increase by SGLT2Is. In addition, similar neutral results were reported regarding renal, pancreatic and hepatocellular cancers as well.^191^

As the use of SGLT2I with the indication for HF has only recently been introduced, most meta-analyses synthesize data from studies of patients with diabetes. Nevertheless, the putative association between SGLT2I use and cancer outcomes of HF patients—with or without diabetes—requires investigation in future meta-analyses. Moreover, as the above meta-analyses assessed only the risk of cancer, future studies should also assess the outcomes of patients with prevalent cancer.

## Effects of digoxin on cancer

Although not considered as a pillar part for HF pharmacotherapy, digoxin is still used in selected HF patients.^1^ Effects of digoxin on cancer have been investigated in a variety of *in vitro* studies, mostly showing anti-cancer properties by causing cell-cycle arrest.^194–200^ These findings were supported by *in vivo* studies, showing inhibition of tumour growth,^200–202^ or reducing distant tumour formation.^203^ Despite the appealing pre-clinical data, meta-analyses on clinical studies show rather contradictory results. For instance, Ahern and colleagues performed an observational study and a meta-analysis showing a significantly increased risk of breast cancer in digoxin users vs. non-users.^204^ This finding was further supported by a meta-analysis also reporting significantly increased risk of breast cancer, lung cancer, and colorectal cancer, but not prostate cancer in association with digoxin use.^205^ In addition, significantly increased all-cause mortality of cancer patients using digoxin was also reported, but no increase in cancer-specific mortality could be detected. It should be emphasized here that these results should be interpreted with caution, as clinical studies may be biased by (i) a higher likelihood of medical contact, and (ii) an intrinsic tendency towards worse outcomes (not only restricted to cancer or cardiovascular outcomes), as patients taking digoxin are on average sicker than those not on this medication.

Overall, there is an apparent discrepancy between the pre-clinical studies (almost unanimously demonstrating anti-cancer effects) and the clinical investigations (showing a tendency towards worse cancer outcomes) regarding effects of digoxin on cancer. This discrepancy highlights the need for increasing the translational value of pre-clinical research, and the reliability of clinical data that are synthesized by meta-analyses.

## Effects of diuretics on cancer

Loop diuretics (e.g. furosemide) are used in HF patients to reduce symptoms and signs of congestion.^1^ The target molecule of furosemide, Na-K-2Cl-transporter has been shown to be expressed on cancer cells, playing a key role in cancer cell growth. Pre-clinical studies have demonstrated anti-cancer effects of furosemide, which was attributed to Na-K-2Cl-transporter inhibition,^206–208^ however, no such effects were seen in clinical studies.^209^ Although thiazides are not the preferred diuretic agents for decongestion purposes in HF, it should be noted that use of hydrochlorothiazide has been brought in association with increased risk of skin cancer,^210^ however, a recent meta-analysis has found no such associations.^211^ In summary, the interaction between diuretics and malignancy remains inconclusive, especially in HF populations, and further investigations are required to validate the interaction between diuretics and cancer.

## Future directions for decreasing cancer burden of heart failure

**Figure 5:**
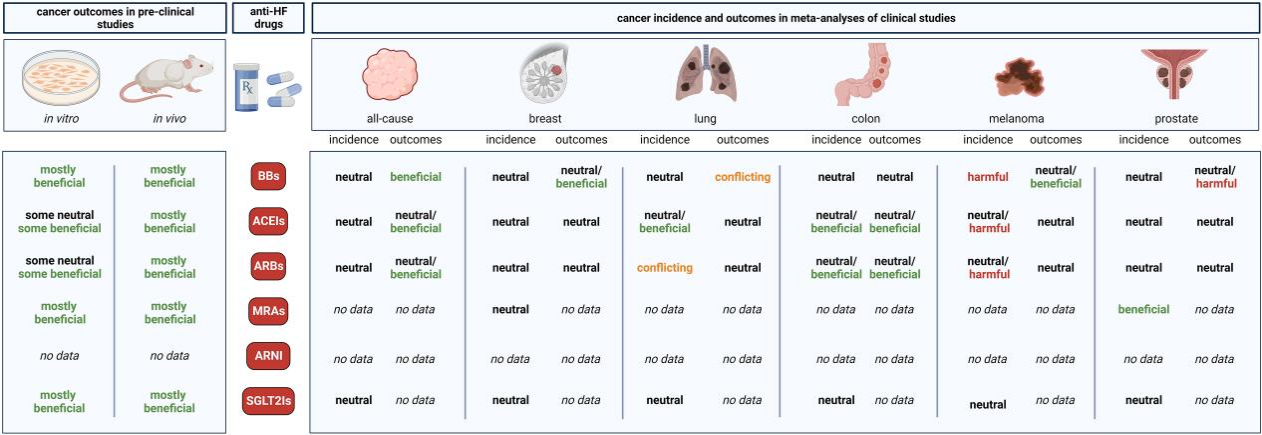
A graphical summary for both the pre-clinical and clinical evidence on the effect of different heart failure pharmacotherapies on cancer. The terms were defined as follows: beneficial: decreases cancer incidence, or improves any patient outcome; neutral: no effect on cancer incidence, or no effect on any patient outcome; harmful: increased incidence or worsening of any patient outcome; conflicting: there are studies showing either benefit or harm on cancer incidence or outcomes. Figure created with BioRender.com.

**Figure 6:**
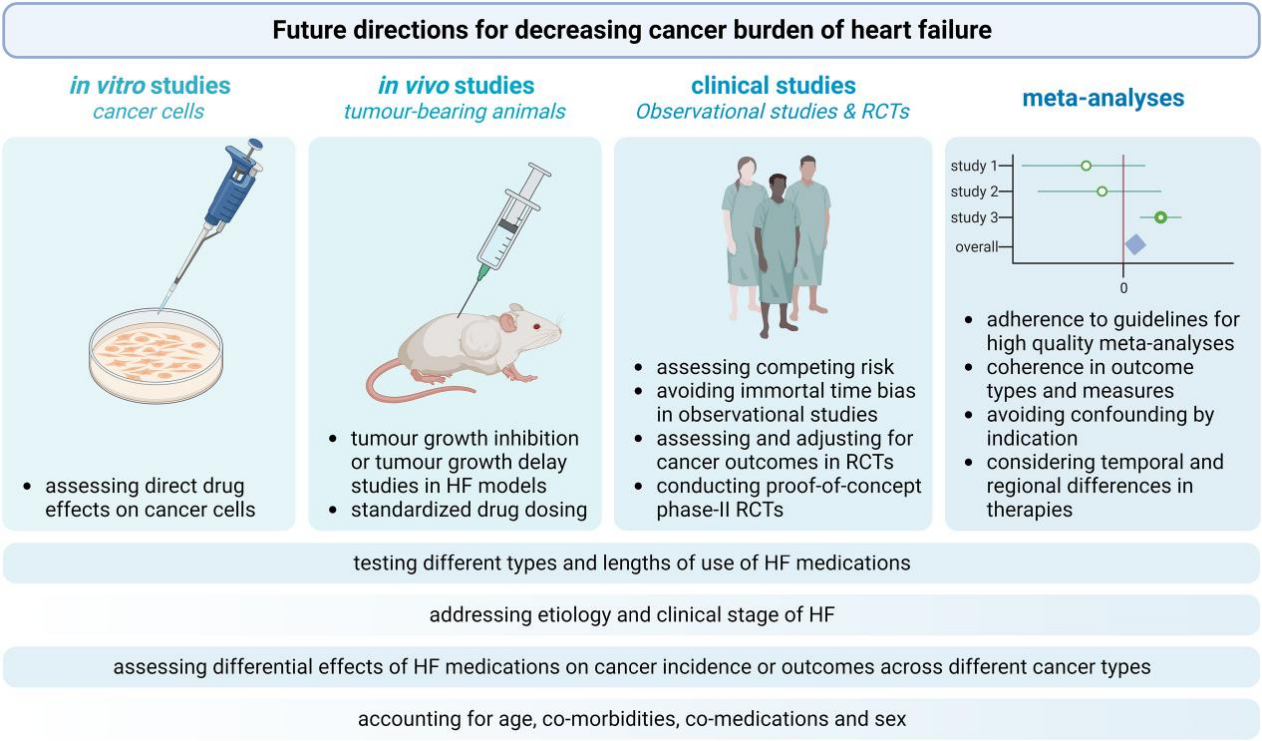
A graphical summary of future directions for decreasing cancer burden of heart failure from pre-clinical studies to clinical investigations and their meta-analyses. Figure created with BioRender.com.

Overall, extensive pre-clinical evidence shows significant anti-cancer effects of all HF GDMT drug classes, nevertheless, no such anti-cancer effects of HF drugs could be confirmed in the clinical reality (*Figure 5*)—a discrepancy that is not at all restricted to the field of cardio-oncology.^212,213^ These findings emphasize the need to conduct pre-clinical studies of higher translational value, and more robust and reliable clinical studies of higher quality, in order to facilitate the formation of recommendations aiming to decrease cancer burden of HF patients (*Figure 6*).

### Considerations for future pre-clinical studies investigating the effect of heart failure drugs on cancer

Pre-clinical *in vivo* and *in vitro* studies complement each other, as *in vitro* studies might fail in taking into account the complexity of the systematic effects of a drug, while better exploring the direct effects on cancer.

Another limitation for translation stems from the lack of standardized practice for drug dosing and administration, as there is a high variety of doses of the same drug between cancer studies. Also, HF drug doses in cancer studies do not necessarily correspond to doses used in HF studies.

In addition, there is a difference in the interpretation of studies where (i) administration starts prior to tumour inoculation (i.e. tumour growth inhibition study), or where (ii) administration starts after an established tumour nodule has already formed (i.e. tumour growth delay study).

Another important aspect for increasing translational value could be the use of *in vivo* tumour-models where cardio-metabolic diseases are also induced to better mimic the frequent clinical situation of HF with co-morbidities. Although pioneer *in vivo* studies assessing tumour growth in animals with prevalent HF induced by either myocardial infarction or transverse aortic constriction (TAC) have already been published, studies assessing the effect of HF medications either alone or combined in such settings should follow.^214–217^ Of note, a TAC model, depending on the severity and length of constriction, could mimic cardiac diseases ranging from non-ischemic HF with reduced or preserved ejection fraction, to aortic stenosis,^218^ which mimic important sub-populations of HF patients.

Finally, to further enhance translational value, (i) differential effects of HF medications either combined or alone, (ii) different etiologies and stages of prevalent HF, (iii) different cancer types, (iv) effects of age, co-morbidities—e.g. hypertension, atrial fibrillation, obesity, and co-medications that are present amongst the majority of HF patients, and (v) sex-based differences should be considered when planning future pre-clinical studies to this field.

### Considerations for future clinical investigations on the effect of heart failure drugs on cancer

Several issues are intrinsic to the study of cardiovascular disease and cancer. First, a major obstacle in the synchronous study of cardiovascular disease and cancer is that, inevitably, successful treatment of the one condition will provide an opportunity for the other condition to progress and become a more important cause of death. For instance, a very powerful HF drug might reduce HF-related outcomes and cardiovascular death, e.g. an MRA. But at the same time, this will provide more opportunity for (latent) cancers to progress and become manifest. This *competing risk* by no means is synonymous to a pro-oncogenic effect of MRA. Vice versa, breast cancer survivors have after 10 years a larger cardiovascular risk than cancer risk.^12^ Since these women have survived one potentially lethal condition, they have beaten the cancer risk (at least for some time), while their cardiovascular risk continues and likely has risen due to aggressive cancer treatments. This complex interplay complicates the simultaneous study of cardiovascular disease and cancer and is very difficult to adjust.

Second, observational studies are prone to biases, a problem that was touched upon by the meta-analysis of Weberpals and colleagues, where BB use significantly increased overall survival and cancer specific survival of cancer patients. However, when observational studies prone to immortal time bias were excluded from the analysis, no significant effects were found for any investigated outcome.^74^

Third, indication for the use of HF drugs was not always attributed to HF exclusively in current observational studies, but rather to hypertension (for ACEIs/ARBs) or diabetes (for SGLT2Is), causing confounding by indication. Therefore, to generate evidence whether and how HF treatment should be modified to improve (or at least not to worsen) cancer-related outcomes in cohorts of HF patients with prevalent cancer is of paramount importance.

Fourth, crucially, cancer outcomes generally are poorly adjudicated in most cardiovascular RCTs, which intrinsically flaws the outputs of any meta-analysis. Systematic assessment of new-onset cancer risk in future HF RCTs is essential to collect valuable information with potential clinical implications, that may require longer follow-up after termination for cardiovascular end-points.^219,220^ On the other hand, systematic assessment of cardiovascular outcomes in future cancer trials is equally important. For instance, the latest RCT with immune checkpoint inhibitors (ICI) did not systematically collect troponin values, while we know that ICI-mediated myocarditis is a potentially lethal side-effect of immunotherapy, which occurs in 2%–5% of all patients.^221^

In general, stratifying patients in clinical investigations based on (i) type and length of use of HF pharmacotherapy of different combinations, (ii) etiology and clinical stage of HF, (iii) cancer type, (iv) age, co-morbidities, and co-medications, is essential because individual patients with co-morbidities may require other types of drugs than HF medications, and importantly (v) based on sex, may more clearly show how HF medications affect cancer incidence, progression, and outcomes, being of paramount importance for clinical decision-making. For instance, the differential sex-related effects of HF medications were addressed by Stolfo and colleagues who showed that female HF patients were more likely to receive BBs, diuretics, and digoxin. Of note, digoxin use was associated with an increased risk of death in females,^222^ but females were less likely to receive RAASIs compared to male HF patients.^223^

Overall, definitive answers would be obtained from proof-of-concept phase II RCTs that directly assess the effects of HF drugs on cancer in HF patients; therefore, such investigations are eagerly waiting to be conducted in the future. Of note, although direct effects of HF drugs on cancer, or the effects of successfully reversed HF on cancer may be hard to dissect in future studies, if the outcome is definitive, this question should be addressed by further mechanistic investigations (*Graphical Abstract*).

Besides guideline-directed pharmacotherapies of HF, investigating other therapeutic options would also facilitate solving this issue. For instance, the interleukin-1beta inhibitor canakinumab has been shown to reduce HF-related hospitalization and mortality,^224,225^ and cumulative incidence of lung cancer in atherosclerotic patients,^226^ raising the question whether targeting inflammation, a shared pathomechanistic pathway of both HF and cancer, could mean a solution for decreasing cancer burden of HF patients.

In conclusion, translatability of pre-clinical studies, and reliability of clinical investigations should be improved to facilitate decision-making on whether and how HF treatment should be modified to decrease cancer incidence and improve cancer outcomes of HF patients.

## Supplementary Material

ehae105_Supplementary_Data

## Declarations

### Data Availability

No data were generated or analysed for or in support of this paper.
