# Supplementary material for: Heart failure pharmacotherapy and cancer: pathways and pre-clinical/clinical evidence
Source: Eur Heart J. 2024 Mar 5;45(14):1224–40. doi: 10.1093/eurheartj/ehae105 (PMC11023004; doi:10.1093/eurheartj/ehae105)
Supplement: ehae105_Supplementary_Data [file ehae105_supplementary_data.zip › Supplementary_table_1_20231211.docx]

| **NCT number** | **Status** | **Study design** | **Population** | **Intervention** | **Comparator** | **Outcome (primary)** |
| --- | --- | --- | --- | --- | --- | --- |
| **NCT04962711** | recruiting | prospective, randomized, controlled | cancer survivors >65 years old with chemotherapy >10 years ago | cardio-oncology disease management plan (optimization of pharmacotherapy, exercise intervention) | usual care | change in exercise capacity |
| **NCT04598646** | not yet recruiting | prospective, randomized, controlled | pediatric, adolescent and young adult (aya) cancer survivors | cario-oncology rehabilitation | behavioral support only | patient access and recruitment; testing- and intervention-related serious adverse events; patient exercise adherence |
| **NCT03882580** | recruiting | prospective, cohort | cancer patients who were evaluated within a cardio-oncology program | - | - | number of patients having a benefit after this specific cardio-oncology check up and follow up |
| **NCT05194111** | recruiting | prospective, randomized, single blinded, phase 1&2 | cancer survivors diagnosed at ≤39 years of age who have stage b heart failure | sacubitril-valsartan | valsartan | evaluating the eligibility requirements and determining the tolerability of treatment with sacubitril-valsartan |
| **NCT02943590** | active, not recruiting | prospective, randomized, double blinded, phase 2 | patients ≥18 years of age with newly diagnosed nhl and hl scheduled to receive anthracycline-based therapy | atorvastatin | placebo | left ventricular ejection fraction (lvef) |
| **NCT05921279** | recruiting | prospective, cohort | adult female patients diagnosed with stage i-iii breast cancer receiving chemotherapy | - | - | number of participants with successful application of guideline-directed cardio-oncology assessments and surveillance |
| **NCT02818517** | recruiting | prospective, cohort | all oncologic patients who were evaluated in the cardio-oncology clinic | ace inhibitors and beta blockers | N/A | echo-global strain; troponin; ace inhibitor and beta blocker treatment effect; bnp |
| **NCT04680442** | recruiting | prospective, randomized, double blinded, phase 2 | patients with stage i-iii her-2 positive breast cancer receiving adjuvant or neoadjuvant therapy with trastuzumab, pertuzumab, or trastuzumab-emtansine, with evidence of left ventricular dysfunction | review by a cardiologist and receiving acei/arb and/or bb, and for dose titration | continuing or holding anti-cancer therapy guided by an adaptation of the 2008 canadian recommendations | the proportion of participants completing trastuzumab, pertuzumab, or trastuzumab-emtansine (t-dm1) as planned at its initiation; co-primary safety outcomes as lvef at the close-out visit, and the composite of nyha class iii or iv heart failure or cardiovascular death |
| **NCT03760588** | active, not recruiting | prospective, randomized, double blinded, phase 2 | women with histological evidence of invasive early breast cancer scheduled for adjuvant therapy with anti-cancer regimens that include anthracyclines | sacubitril-valsartan | placebo | change in left ventricular ejection fraction by cardiovascular magnetic resonance |
| **NCT03186404** | active, not recruiting | prospective, randomized, double blinded, phase 2 | patients with malignancies requiring anthracycline based chemotherapy with a curative intent: (breast cancer; aggressive lymphomas; leukemia; sarcoma) and with high cardiovascular risk | atorvastatin | placebo | cardiac mri measured lvef within 4 weeks of anthracycline completion |
| **NCT02717507** | active, not recruiting | prospective, randomized, double blinded, phase 2 | cancer diagnosis < 22 years of age that completed cancer treatment ≥2 years prior enrollment, lifetime cumulative anthracycline dose of ≥250 mg/m^2^ doxorubicin equivalent | carvedilol | placebo | left ventricular thickness-dimension ratio derived from echocardiogram |
| **NCT04262830** | recruiting | prospective, cohort | long-term childhood cancer survivors treated with anthracyline therap | - | - | left ventricular ejection fraction |
| **NCT05851053** | recruiting | cross-sectional, cohort | breast cancer survivors matched with women of the same age (±1 year), but who had no history of cancer or cancer treatment (chemotherapy or radiotherapy) | - | - | left ventricular systolic dysfunction |
| **NCT04055636** | recruiting | prospective, cohort | male and female more than 18 years old with verified cancer or with non-toxic dilated cardiomyopathy (control group) | - | - | all-cause mortality; heart transplantation; cardioverter-defibrillator implantation; hospitalization with heart failure decompensation |
| **NCT05298072** | not yet recruiting | cohort | initial diagnosis of breast cancer; planned anthracycline-based therapy; first-line chemotherapy; first visit before initiation of chemotherapy | N/A | N/A | cardiotoxicity during observational period |
| **NCT06005259** | not yet recruiting | prospective, randomized, triple masked, phase 4 | patients of >18 years of age diagnosed with cancer indicated for anthracycline chemotherapy treatment | spironolactone | placebo | incidence of cardiotoxicity, defined as: a decrease in ejection fraction by 10% or more to lvef < 50%, as seen on transthoracic echocardiogram; or relative drop in global longitudinal strain greater than 15% compared to baseline, observed on transthoracic echocardiogram; new increase in cardiac biomarkers |
| **NCT05892146** | recruiting | prospective, randomized, quadruple masked | patients of 20-65 years of age who are newly diagnosed with breast cancer or lymphoma and never accepted anti-cancer therapy | sacubitril-valsartan | conventional therapy | change in absolute global longitudinal strain value measured by left ventricular global peak systolic longitudinal strain |
| **NCT05607017** | not yet recruiting | phase 1, single group, open label | patients who are receiving radiation therapy as part of standard of care treatment for breast cancer | losartan | - | extracellular volume of myocardial fibrosis measured by cardiac mri |
| **NCT04737265** | recruiting | prospective, randomized, open label, phase 1&2 | patients of ≥18 years of age diagnosed with breast cancer or lymphoma (any subtype), planned to receive an anthracycline based chemotherapy regimen | biomarker guided intervention | usual care | recruitment rate; retention rate; adherence rate; compliance rate; maximum tolerated dose; incidence of adverse events |
| **NCT02962661** | recruiting | prospective, randomized, open label, phase 1 | patients with lvef ≤40% with nyha class i, ii and iii, documented from treatment with anthracyclines for any malignancy at any dose at any time without evidence of other causes of cardiomyopathy; for patients who have received trastuzumab: persistent lv dysfunction must be present 90 days after discontinuation of trastuzumab; treated with appropriate maximal medical therapy for heart failure. | intravenous or transendocardial implantation of human mesenchymal stem cells | usual care | incidence of adverse events, change in left ventricular ejection fraction |
| **NCT05732051** | recruiting | prospective, randomized, triple masked, phase 2 | women with metastatic breast cancer (stage iv breast cancer) scheduled for anthracycline-containing chemotherapy | nicotinamide riboside | placebo | reduction in left ventricular systolic function measured by cardiovascular magnetic resonance |
| **NCT04023110** | active, not recruiting | prospective, randomized, open label, phase 1 | females of at least 18 years old diagnosed with stage i-iii breast cancer with treatment plan to include therapy with anthracyclines and/or trastuzumab in the adjuvant or neo-adjuvant setting | carvedilol | usual care | left ventricular ejection fraction; treatment adherence; adverse events |
| **NCT05880160** | not yet recruiting | randomized, open label | adult patients with prior diagnosis of human epidermal growth factor receptor 2 - targeted therapy related cardiac dysfunction, who currently receive standard heart failure/cardioprotective medications | cancer treatment withdrawal | cancer treatment continuation | number of participants with relapse in cardiotoxicity, defined based on international cardio-oncology society 2021 guidelines |
| **NCT05507879** | recruiting | prospective, cohort | patients with breast cancer who have previously received chemotherapy or are about to be treated with chemotherapy | - | - | significance oftrpc6 coding sequencing |
| **NCT02610426** | recruiting | retrospective, case-control | patients with breast cancer enrolled on e5103 with or without congestive heart failure | - | - | identification of rare coding variants of large effect that predict the risk of chf |
| **NCT05377320** | not yet recruiting | prospective, cohort | cancer survivors at intermediate, high, or very high risk for developing cardiovascular disease will pursue a cardio-oncology visit | clinical decision aid | usual care | medication use, imaging surveillance |
| **NCT05465031** | not yet recruiting | prospective, randomized, double blinded, phase 4 | female patients of ≥18 years of age with histologically confirmed breast cancer and complete assessment of tumor phenotype gradin ia-iiic or oligometastatic grade iv with a plan of using systemic treatment (preoperative, postoperative or combined) with anthracyclines and/or anti-her2 drugs | sacubitril-valsartan | placebo | decrease in left ventricular ejection fraction ≥ 5% |

**Supplementary table 1.:** On-going clinical studies of cardio-oncology. The following search string was used on ClinicalTrials.gov (accessed: 2023.09.18): ’heart failure’ as condition or disease, AND ’cancer’ as other term. Search was restricted to either ’recruiting’, or ’active, not recruiting’, or ’not yet recruiting’. A total of 98 registered trials was screened for eligibility, of which 27 on‑going studies were related to cardio-oncology. Most of the studies were designed to assess interventions that may decrease cancer therapy-related cardiovascular diseases, with some them testing the effects of HF medications compared to placebo/usual care.
