# Supplementary material for: Heart failure pharmacotherapy and cancer: pathways and pre-clinical/clinical evidence
Source: Eur Heart J. 2024 Mar 5;45(14):1224–40. doi: 10.1093/eurheartj/ehae105 (PMC11023004; doi:10.1093/eurheartj/ehae105)
Supplement: ehae105_Supplementary_Data [file ehae105_supplementary_data.zip › Supplementary_table_2_20231211.docx]

| **Effects of BBs on cancer assessed *in vivo*** | | | | | | | | |
| --- | --- | --- | --- | --- | --- | --- | --- | --- |
| **Cancer type** | **Study** | **Cell line** | **Animal model** | **Treatment** | **Tumor volume measurement** | **Major outcomes for treatment** | **Suggested mechanism of action** | **Synergism** |
| **Breast cancer** | **R. D. Gillis et al. (2021)**^1^ | MDA-MB-231 | orthotopic xenograft tumour implantation in mice | carvedilol  (2 mg/kg/day) | bioluminescence | carvedilol reduced primary tumour growth and metastsis only in SN activation | - | - |
| **Breast cancer** | **M. Tibensky et al. (2021)**^2^ | - | N-methyl-N-nitrosourea-induced breas cancer in rats | propranolol  (20 mg/kg five times a week) | caliper | propranolol prolonged time of first palpable tumor detection, reduced tumor incidence | increased Caspase-3 gene expression | - |
| **Breast cancer** | **R. P. Dawes et al. (2020)**^3^ | - | MMTV-PyMT mice, spontaneous | nadolol  (1.5 mg in s.c.  60-day continuous release pellet) | terminal primary tumour weight, metastasis histology | nadolol increased primary tumour weight and metastases | increased exosomal TGFß2 | - |
| **Breast cancer** | **D. Liu et al. (2015)**^4^ | MCF-7/Her2 | 17β-estradiol containing pellets plus orthotopic xenograft tumour implantation in mice | propranolol  (2 mg/kg i.p. twice a week) | caliper | propranolol alone did not affect cancer growth | inhibition of Akt, ERK and mTOR phosphorylation | propranolol reversed trastuzumab-resitence of cancer: propranolol+trastuzumab decreased tumour volume |
| **Breast cancer** | **D. M. Lamkin et al. (2015)**^5^ | MDA-MB-231HM | orthotopic xenograft tumour implantation in mice | propranolol  (2 mg/kg/day s.c.) | caliper, bioluminescence | propranolol alone did not affect cancer growth, but inhibited phentolamine-induced tumour growth and metastases | - | - |
| **Breast cancer** | **J. P. Campbell et al. (2012)**^6^ | MDA-MB-231 | heterotopic xenograft tumour implantation in Foxn1^nu^ BALB/c mice | propranolol  (0.5 g/L in drinking water ad libitum) | caliper | proppranolol inhibited sympathetic activation-induced tumour growth and metastastic bone colonization | - | - |
| **Lung cancer** | **D. Hu et al. (2021)**^7^ | LLC | heterotopic syngraft tumour implantation in mice | propranolol  (0.5 g/L in drinking water ad libitum) | caliper | propranolol alone inhibited tumour growth | increased NK cell cytotoxicity | grain-sized moxibustion ± propranolol combination: no difference in anti-cancer efficacy |
| **Lung cancer** | **M. Niu et al. (2021)**^8^ | H1975 or  PC-9 or  PC-9/AZDR | heterotopic xenograft tumour implantation in mice | nebivolol  (10 mg/kg i.p. six times a week) | caliper | nebivolol alone inhibited tumor growth and cell proliferation | nebivolol upregulated FBXL2 expression which inhibits EGFR-driven tumour growth | nebivolol + osimertinib/Grp94-inhibitor-1: strong inhibitory effects on osimertinib-resistant cells |
| **Lung cancer** | **A. Chang et al. (2015)**^9^ | A549 | heterotopic xenograft tumour implantation in mice | carvedilol  (26 mg/kg/day, p.o.) | caliper | carvedilol alone reduced tumor volume | - | - |
| **Colorectal cancer** | **K. Y. Fjæstad et al. (2022)**^10^ | MC38 | heterotopic syngraft tumour implantation in mice | propranolol  (0.5 g/L in drinking water ad libitum) | caliper | propranolol alone decreased tumour growth | anti-angionetic effect by decreasing intratumoral Vegfa gene expression | proranolol increased anti-cancer efficacy of anti-CTLA4 therapy |
| **Colorectal cancer** | **C. R. MacDonald et al. (2019)**^11^ | CT26.CL25 | heterotopic syngraft tumour implantation in mice | propranolol  (10 mg/kg/day i.p.) | caliper | propranolol was used only in combination with radiation | higher expression of granzyme-B in tumour microenvironment | propranolol improved anti-cancer efficacy of radiation |
| **Colorectal cancer** | **L. Sorski et al. (2016)**^12^ | CT26 | syngraft tumour injection to spleen or portal vein in mice | propranolol 5 mg/kg subcutaneously in a single dose | number of surface hepatic metastases, liver weight | propranolol alone did not affect metastasis | NK cell activaiton | etodolac+propranolol combination: improved host resistance to metastasis |
| **Melanoma** | **F. Moisan et al. (2021)**^13^ | A375 | orthotopic xenograft tumour implantation in mice | propranolol  (2 mg/kg/day) | caliper | propranolol was used only in combination with bevacizumab | no significant anti-cancer activity | bevacizumab+propranolol combination: no significant anti-cancer effect |
| **Melanoma** | **K. H. Cleveland et al. (2018)**^14^ | A375 with BRAF^V600E^ mutation | orthotopic xenograft tumour implantation in mice | carvedilol 600 ug/day p.o. | caliper | carvedilol decreased tumour growth | - | - |
| **Melanoma** | **X. Kuang et al. (2017)**^15^ | A375 | orthotopic xenograft tumour implantation in mice | propranolol 2mg/kg/day p.o. | caliper | propranolol alone did not affect tumour growth | - | propranolol enhanced anti-tumour effect of low dose sunitinib |
| **Melanoma** | **K. M. Kokolus et al. (2017)**^16^ | B16-F10 | orthotopic syngraft tumour implantation | metoprolol  (10 mg/kg/day i.p.) | caliper | metoprolol alone did not affect tumour growth | - | metoprolol did not enhance anti-tumour effects of αPD-1+IL-2 combination |
| **Melanoma** | **K. M. Kokolus et al. (2017)**^16^ | B16-F10 | orthotopic syngraft tumour implantation | propranolol  (10 mg/kg/day i.p.) | caliper | propranolol alone decreased tumor growth | - | propranolol enhanced anti-tumour effects of αPD-1, αPD-1+IL-2 combination, but not IL-2 |
| **Melanoma** | **S. Maccari et al. (2017)**^17^ | B16-F10 | orthotopic syngraft tumour implantation | propranolol  (10, 20, 30, 40 mg/kg/day i.p.) | caliper | propranolol decreased tumour growth in a biphasic dose (10 and 40 mg/kg/day) | influencing systemic vascular resistance | - |
| **Melanoma** | **L. J. Wrobel et al. (2016)**^18^ | - | MT/ret mice, spontaneous | propranolol  (0.5 g/L in drinking water ad libitum) | primary tumor occurrence (primary tumour-free survival), metastases | propranolol prolonged primary tomour-free survival, and delayed formation of metastases | reduced myeloid infiltration, increased granzyme-B-expressing lymphid cells in primary tumours, increased NK and cytotoxic T-cells in metastases | - |
| **Melanoma** | **C. Zhou et al. (2016)**^19^ | A375 or human patient derived melanoma | orthotopic xenograft tumour implantation | propranolol  (2 or 10 mg/kg/day i.p.) | caliper | propranolol decreased tumour growth (lower dose was more effective) | inhibited proliferation, induced apoptosis and promoted cell necrosis in tumors | - |
| **Melanoma** | **L. J. Wrobel et al. (2015)**^20^ | human patient derived melanoma | orthotopic xenograft tumour implantation | propranolol  (0.5 g/L in drinking water ad libitum) | caliper | propranolol decreased tumour growth and metastases | inhibited proliferation and angiogenesis, induced apoptosis through upregulation of TP53 and downregulation of Akt3 and HIF1a | - |
| **Melanoma** | **G-H. Deng et al. (2014)**^21^ | B16-F1 | orthotopic syngraft tumour implantation | propranolol  (1 μmol/100 g/day by microosmotic pumps) | caliper | propranolol alone did not affect tumour growth, but inhibited tumor growth promoting effect of norepinephrine | - | - |
| **Prostate cancer** | **D. Palm et al. (2006)**^22^ | PC-3 | heterotopic xenograft tumour implantation in mice | propranolol  (1 μmol/100 g bw s.c. by microosmotic pumps) | bioluminescence | significantly decreased lymph-node metastases and inhibited norepinephrine-induced tumour growth | - | - |
|  | | | | | | | | |
|  | | | | | | | | |
|  | | | | | | | | |
|  | | | | | | | | |
|  | | | | | | | | |
|  | | | | | | | | |
|  | | | | | | | | |
|  | | | | | | | | |
|  | | | | | | | | |
| **Effects of ACEIs on cancer assessed *in vivo*** | | | | | | | | |
| **Lung cancer** | **K. Nakaya et al. (2016)**^23^ | A549 | heterotopic xenograft tumour implantation in severe combined immunodeficiency mice | captopril  (3 mg/mouse/day) | 18F-FDG-PET/CT imaging | captopril reduced tumor volume and metabolic tumor volume | - | - |
| **Lung cancer** | **S. Attoub et al. (2008)**^24^ | LMN35 | heterotopic xenograft tumour implantation in mice | captopril  (2.8 mg/kg i.p.  6 days a week) | caliper | captopril reduced primary tumor volume and angiogenesis, tendentially reduced lymph node metastasis | - | - |
| **Lung cancer** | **R. R. Kohl et al. (2007)**^25^ | A549 | heterotopic xenograft tumour implantation | ramipril  (2.5 mg/kg/day in drinking water ad libitum) | caliper | ramipril did not affect tumor response to radiation, but reduced radiation injury of normal tissue | - | - |
| **Colorectal cancer** | **Y. Yang et al. (2020)**^26^ | SW620 | heterotopic xenograft tumour implantation in mice | enalapril  (0.6 mg/kg/day p.o.) | caliper | enalapril alone did not affect tumour growth | enalapril + 5-FU:   EMT suppression, NF-κB/STAT3 suppression, MMP-9 and MMP-2 suppression | enalapril+5-FU combination: reduced tumour growth, proliferation, angiogenisis, metastasis |
| **Colorectal cancer** | **S. L. Koh et al. (2014)**^27^  **G. E. Riddiough et al. (2021)**^28^ | mouse colorectal cancer cells, developed via dimethyl-hydrazine induction of colon carcinoma in CBA mice | intrasplenic injeciton of cancer cells | captopril  (250 mg/kg/day i.p.) | stereometric tumour burden analysis | captopril decreased metastasis burden of regenerating liver remnant | captopril increased PD-1 expression in T-cells, modulates myeloid-derived suppressor cell populaitons | - |
| **Colorectal cancer** | **D. L. V. Ardila et al. (2020)**^29^ | mouse colorectal cancer cells, developed via dimethyl-hydrazine induction of colon carcinoma in CBA mice | intrasplenic injeciton of cancer cells | captopril  (750 mg/kg/day i.p.) | stereometric tumour burden analysis | captopril reduced metastatic growth and tumor viability, modulates spatio-temporal infiltration of lymphocytes into tumour | - | - |
| **Colorectal cancer** | **T. Kochi et al. (2014)**^30^ | - | azoxymethane-induced colonic premalignant lesions in diabetic and hypertensive rats | captopril  (8 mg/kg/day in drinking water ad libitum) | aberrant cryptic foci size and density | captopril inhibits early size and density of aberrant cryptic foci | reduced colonic epithelial expression of AT1R, ACE, TNF-alpha, IL-18, MCP-1, iNOS and VEGF mRNA expression | - |
| **Colorectal cancer** | **S. Wen Wen et al. (2013)**^31^ | mouse colorectal cancer cells, developed via dimethyl-hydrazine induction of colon carcinoma in CBA mice | intrasplenic injeciton of cancer cells | captopril  (750 mg/kg/day i.p.) | stereometric tumour burden analysis | captopril decreased tumor load | increased number of liver AT1R-positive Kuppfer cells | - |
| **Melanoma** | **P. J. Wysock et al. (2006)**^32^ | B78-H1 | heterotopic syngraft tumour implantation | captopril  (25, or 60 mg/kg/day) | caliper | captopril did not affect tumour growth | - | - |
|  | | | | | | | | |
|  | | | | | | | | |
|  | | | | | | | | |
|  | | | | | | | | |
|  | | | | | | | | |
|  | | | | | | | | |
|  | | | | | | | | |
|  | | | | | | | | |
| **Effects of ARBs on cancer assessed *in vivo*** | | | | | | | | |
| **Breast cancer** | **W. Li et al. (2021)**^33^ | MCa-M3C | orthotopic syngraft tumour implantation in mice | losartan  (40 mg/kg/day p.o.) | caliper | losartan alone did not affect tumour growth | - | losartan enhanced tumour response to radiation therapy |
| **Breast cancer** | **L. E. Mainetti et al. (2020)**^34^ | M-234p or M-406 | heterotopic syngraft tumour implantation | losartan  (150-200 mg/kg/day p.o.) | caliper | losartan alone did not affect tumour growth | - | losartan enhanced tumour response to cyclophosphamide: increased number of TUNEL+ and Foxp3+ cells, decreased number of HIF1-α positive cells, lower % of αSMA+ positive cells |
| **Breast cancer** | **X-J. Cai et al. (2021)**^35^ | NIH 3T3 & 4T1 (3:1) | heterotopic syngraft tumour implantation | candesartan  (20 mg/kg/day p.o.) | caliper | candesartan decreased tumour growth and metastasis | tumor vessel normalization and depletion of ECM, inhibition of metastasis by eliminating collagen‑I | candesartan + pegylated liposome–encapsulated zoledronic acid combination: stronger decrease in active TGF-β1 but no significant improvement over single treatment |
| **Breast cancer** | **T. Takiguchi et al. (2021)**^36^ | 4T1-Luc | orthotopic syngraft tumour implantation | valsartan  (60 mg/kg/day p.o.) | micro-computed tomography imaging | valsartan attenuates tumour growth and metastases induced by angiotensin-II | attenuated upregulation of protein expressions caused by Ang II (c-Myc, cyclin D1, fibronectin, vimentin, αSMA and Snail) | - |
| **Breast cancer** | **Y. Ma et al. (2019)**^37^ | MDA-MB-231, or 4T1 | orthotopic xenograft tumour implantation | losartan  (40 mg/kg/day p.o.) | caliper, bioluminescence imaging | losartan reduces tumor growth and lymph node metastasis | reduced CXCR4/SDF-1α expression | - |
| **Breast cancer** | **R. Coulson et al. (2017)**^38^ | **-** | MPA+DMBA-induced breast cancer | losartan  (600 mg/L in drinking water ad libitum) | timet o first tumour incidence | losartan delayed tumor onset, inhibited progression | decreased cytokine production, decreased TGFβ1, integrin β3, connective tissue growth factor | - |
| **Breast cancer** | **T. Xia et al. (2018)**^39^ | 4T1 | heterotopic syngraft tumour implantation | losartan  (2.5, or 10 mg/kg/day i.v.) | caliper | losartan did not affect tumour growth | - | - |
| **Breast cancer** | **E. Oh et al. (2016)**^40^ | MCF7, or AGTR1-overexpressing MCF7 | heterotopic xenograft tumour implantation | losartan  (90 mg/kg i.p. three times a week) | caliper | losartan inhibited tumor growth and angiogenesis upregulated by AGTR1 overexpression | increasing mesenchymal markers and decreasing E-cadherin levels | - |
| **Breast cancer** | **D. R. Rhodes (2009)**^41^ | MCF7-AGTR1 | orthotopic xenograft tumour implantation | losartan  (90 mg/kg/day) | caliper | losartan reduced early and late tumor growth | - | - |
|  |  | MCF7-Gus | orthotopic xenograft tumour implantation | losartan  (90 mg/kg/day) | caliper | no effect on tumor growth | - | - |
| **Lung cancer** | **D. Volonte et al. (2021)**^42^ | - | K-RasLA2-G12D mice | losartan  (50 mg/kg/day i.p.) | number of surface lung tumors | losartan inhibits lung tumor formation | downregulation of phospho(Tyr705)-STAT3, upregulated AGT and HMGA1 mRNAs | - |
| **Lung cancer** | **D. P. Regan et al. (2019)** | 4T1-luc, or CT26-luc, or CT26-GFP | heterotopic syngraft tumour implantation | losartan (60mg/kg/day i.p.) | bioluminescence imaging | losartan supressed pulmonary metastasis growth | sustained blockade of inflammatory monocyte recruitment | not performed |
| **Colorectal cancer** | **E. Tabatabai et al. (2021)**^43^ | CT-26 | heterotopic syngraft tumour implantation | candesartan  (6.5 mg/kg/day i.p.) | caliper | candesartan inhibited tumour growth | decreasing collagen content, inducing tumor necrosis and changing the oxidant/antioxidant balance in tumor tissue | candesartan enhanced the anti-tumor effects of 5-FU |
| **Colorectal cancer** | **M. Hashemzehi et al. (2021)**^44^ | HT-29 | heterotopic xenograft tumour implantation | losartan  (90 mg/kg/day i.p.) | caliper | losartan inhibited tumor growth | inhibiting angiogenesis and changing the oxidant/anti-oxidant balance in tumor tissue | losartan enhanced the anti-tumor effects of 5-FU |
| **Colorectal cancer** | **U. Dougherty et al. (2019)**^45^ | - | Apc^+/LoxP^ ; Cdx2P-Cre mice | losartan  (160 mg/L in drinking water ad libitum) | colonoscopy | losartan decreased cancer incidence and tumour size | reduced pAKT and pERK | losartan+vitamin D combination: tumor multiplicity was numerically less, reduced β-catenin expression and ADAM17 from VD single treatment was not observed in the combination treatment |
| **Colorectal cancer** | **F. Asgharzadeh et al. (2022)**^46^ | CT-26 | heterotopic syngraft tumour implantation in mice | valsartan  (40 mg/kg/day p.o.) | caliper | valsartan reduced tumour growth | induction of apoptosis via inhibiting RAS pathway | valsartan increased the anti-cancer efficacy of 5-FU |
| **Colorectal cancer** | **S. C. W. Stevens et al. (2015)**^47^ | C26 | heterotopic syngraft tumour implantation in mice | losartan  (10 mg/kg in drinking water ad libitum) | terminal tomour weight | losartan reduced tumor weight | reduction in adenocarcinoma cell proliferation | - |
| **Melanoma** | **S. Ishikane et al. (2018)**^48^ | B16-F10 | syngraft lung metastasis model (tail vein injection) | valsartan (10, or 20, or 40 mg/kg/day in drinking water ad libitum) | nodule counting | valsartan alone did not affect metastases, but inhibited angiotensin-II-induced metastatic colony formation | - | - |
| **Melanoma** | **A. H. Otake et al. (2010)**^49^ | B16-F10 | orthotopic syngraft tumour implantation | losartan  (75 mg/kg/day doubled every 3 days until 300 mg/kg/day p.o.) | caliper | losartan decreased tumor volume, no decrease in metastasis | lower new vessel formation in tumors | - |
| **Prostate cancer** | **A. Alhusban et al. (2014)**^50^ | PC3 | heterotopic xenograft tumour implantation | candesartan  (6.5 mg/kg/day i.p.) | caliper | candesartan inhibited tumour growth | inhibited VEGF mRNA expression Independent of AT2-R activation | - |
| **Prostate cancer** | **S. Takahashi et al. (2012)**^51^ | - | TRAP rats, spontaneous | telmisartan or candesartan  (2 or 10 mg/kg/day in drinking water ad libitum) | counting total acini in each prostatic lobe | both doses of candesartan or telmisartan attenuated prostate carcinogenesis | activation of caspases, inactivated p38 MAPK, down-regulated androgen receptors | - |
|  |  |  |  |  |  |  |  |  |
|  |  |  |  |  |  |  |  |  |
|  |  |  |  |  |  |  |  |  |
|  |  |  |  |  |  |  |  |  |
|  |  |  |  |  |  |  |  |  |
|  |  |  |  |  |  |  |  |  |
|  |  |  |  |  |  |  |  |  |
|  |  |  |  |  |  |  |  |  |
|  | | | | | | | | |
|  | | | | | | | | |
| **Effects of MRAs on cancer assessed *in vivo*** | | | | | | | | |
| **Colorectal cancer** | **W-H. Leung et al. (2013)**^52^ | - | C57BL/6J-APCMin/J mice | spironolactone (1.25 mg/mouse i.p. twice a week) | polyp counting | spironolactone decreased number of polyps | increased the expression of Raet1 ligand in the polyps, up-regulation of NKG2DL expression independent of the MR pathway | - |
|  |  | HT29, or HCT116 | heterotopic xenograft tumour implantation in mice | spironolactone (1.25 mg/mouse i.p. twice a week) | bioluminescence imaging | spironolactone inhibits tumor metastasis | antimetastatic effect is independent of MR but activation of the ATM–ATR pathway requires the activation of RXRγ | - |
|  |  |  |  |  |  |  |  |  |
|  |  |  |  |  |  |  |  |  |
|  |  |  |  |  |  |  |  |  |
|  |  |  |  |  |  |  |  |  |
|  |  |  |  |  |  |  |  |  |
|  |  |  |  |  |  |  |  |  |
|  |  |  |  |  |  |  |  |  |
|  | | | | | | | | |
| **Effects of SGLT2Is on cancer assessed *in vivo*** | | | | | | | | |
| **Breast cancer** | **J. Zhou et al. (2020)**^53^ | MCF-7 | heterotopic xenograft tumour implantation | dapagliflozin  (100 mg/kg/day p.o.) | caliper | dapagliflozin delayed tumor growth | - | - |
| **Breast cancer** | **A. R. Nasiri et al. (2019)**^54^ | E0771 | heterotopic syngraft tumour implantation in obese and non-obese mice | dapagliflozin 2.5 mg/kg/day in drinking water ad libitum | caliper | dapagliflozin slows tumor growth in insulin-dependent manner | reversing hyperinsulinemia | - |
| **Colorectal cancer** | **A. R. Nasiri et al. (2019)**^54^ | MC38 | heterotopic syngraft tumour implantation in obese and non-obese mice | dapagliflozin 2.5 mg/kg/day in drinking water ad libitum | caliper | dapagliflozin slows tumor growth in insulin-dependent manner | reversing hyperinsulinemia | - |
| **Colorectal cancer** | **J. Korfhage et al. (2022)**^55^ | - | C57BL/6J-Apc^min^ mice, spontaneous | chow containing 180 parts per million canagliflozin for 30-72 days | intestinal section imaging | canagliflozin increased intestinal adenoma burden in female mice | - | - |
| **Prostate cancer** | **C. Scafoglio et al. (2015)**^56^ | PC3 | heterotopic xenograft tumour implantation | canagliflozin (30 mg/kg/day p.o.) | microPET/CT imaging | canagliflozin reduced tumor growth | increased central necrotic area | canagliflozin+gemcitabine combination: higher reduction in tumor growth |

**Supplementary table 2.:** *In vivo* studies investigating the effect of guideline-directed HF pharmacotherapies on cancer.

**References:**

1. Gillis, R. D. *et al.* Carvedilol blocks neural regulation of breast cancer progression in vivo and is associated with reduced breast cancer mortality in patients. *Eur. J. Cancer* **147**, 106–116 (2021).

2. Tibensky, M. *et al.* Chronic propranolol treatment moderately attenuated development of N-methyl-N-nitrosourea-induced mammary carcinoma in female rats. *Anticancer. Drugs* **32**, 1011–1018 (2021).

3. Dawes, R. P. *et al.* Chronic Stress Exposure Suppresses Mammary Tumor Growth and Reduces Circulating Exosome TGF-β Content via β-Adrenergic Receptor Signaling in MMTV-PyMT Mice. *Breast Cancer (Auckl).* **14**, 1178223420931511 (2020).

4. Liu, D. *et al.* β2-AR signaling controls trastuzumab resistance-dependent pathway. *Oncogene* **35**, 47–58 (2016).

5. Lamkin, D. M. *et al.* α2-Adrenergic blockade mimics the enhancing effect of chronic stress on breast cancer progression. *Psychoneuroendocrinology* **51**, 262–270 (2015).

6. Campbell, J. P. *et al.* Stimulation of host bone marrow stromal cells by sympathetic nerves promotes breast cancer bone metastasis in mice. *PLoS Biol.* **10**, e1001363 (2012).

7. Hu, D. *et al.* Grain-sized moxibustion promotes NK cell antitumour immunity by inhibiting adrenergic signalling in non–small cell lung cancer. *J. Cell. Mol. Med.* **25**, 2900–2908 (2021).

8. Niu, M. *et al.* FBXL2 counteracts Grp94 to destabilize EGFR and inhibit EGFR-driven NSCLC growth. *Nat. Commun.* **12**, 5919 (2021).

9. Chang, A. *et al.* Prevention of skin carcinogenesis by the β-blocker carvedilol. *Cancer Prev. Res.* **8**, 27–36 (2015).

10. Fjæstad, K. Y. *et al.* Blockade of beta-adrenergic receptors reduces cancer growth and enhances the response to anti-CTLA4 therapy by modulating the tumor microenvironment. *Oncogene* **41**, 1364–1375 (2022).

11. MacDonald, C. R. *et al.* Adrenergic receptor signaling regulates the response of tumors to ionizing radiation. *Radiat. Res.* **191**, 585–589 (2019).

12. Sorski, L. *et al.* Reducing liver metastases of colon cancer in the context of extensive and minor surgeries through β-adrenoceptors blockade and COX2 inhibition. *Brain Behav. Immun.* **58**, 91–98 (2016).

13. Moisan, F. *et al.* Critical role of Aquaporin-1 and telocytes in infantile hemangioma response to propranolol beta blockade. *Proc. Natl. Acad. Sci. U. S. A.* **118**, e2018690118 (2021).

14. Cleveland, K. H. *et al.* Phosphoproteome profiling provides insight into the mechanism of action for carvedilol-mediated cancer prevention. *Mol. Carcinog.* **57**, 997–1007 (2018).

15. Kuang, X. *et al.* Propranolol enhanced the anti-tumor effect of sunitinib by inhibiting proliferation and inducing G0/G1/S phase arrest in malignant melanoma. *Oncotarget* **9**, 802–811 (2018).

16. Kokolus, K. M. *et al.* Beta blocker use correlates with better overall survival in metastatic melanoma patients and improves the efficacy of immunotherapies in mice. *Oncoimmunology* **7**, e1405205 (2018).

17. Maccari, S. *et al.* Biphasic effects of propranolol on tumour growth in B16F10 melanoma-bearing mice. *Br. J. Pharmacol.* **174**, 139–149 (2017).

18. Jean Wrobel, L. *et al.* Propranolol induces a favourable shift of anti-tumor immunity in a murine spontaneous model of melanoma. *Oncotarget* **7**, 77825–77837 (2016).

19. Zhou, C. *et al.* Propranolol induced G0/G1/S phase arrest and apoptosis in melanoma cells via AKT/MAPK pathway. *Oncotarget* **7**, 68314–68327 (2016).

20. Wrobel, L. J. & Le Gal, F. A. Inhibition of Human Melanoma Growth by a Non-Cardioselective β-Blocker. *J. Invest. Dermatol.* **135**, 525–531 (2015).

21. Deng, G.-H. *et al.* Exogenous norepinephrine attenuates the efficacy of sunitinib in a mouse cancer model. *J. Exp. Clin. Cancer Res.* **33**, 21 (2014).

22. Palm, D. *et al.* The norepinephrine-driven metastasis development of PC-3 human prostate cancer cells in BALB/c nude mice is inhibited by β-blockers. *Int. J. Cancer* **118**, 2744–2749 (2006).

23. Nakaya, K., Otsuka, H., Kondo, K., Otani, T. & Nagata, M. Tumor growth-inhibitory effect of an angiotensin-converting enzyme inhibitor (captopril) in a lung cancer xenograft model analyzed using 18F-FDG-PET/CT. *Nucl. Med. Commun.* **37**, (2016).

24. Attoub, S. *et al.* Captopril as a Potential Inhibitor of Lung Tumor Growth and Metastasis. *Ann. N. Y. Acad. Sci.* **1138**, 65–72 (2008).

25. Kohl, R. R., Kolozsvary, A., Brown, S. L., Zhu, G. & Kim, J. H. Differential Radiation Effect in Tumor and Normal Tissue after Treatment with Ramipril, an Angiotensin-Converting Enzyme Inhibitor. *Radiat. Res.* **168**, 440–445 (2007).

26. Yang, Y. *et al.* Enalapril overcomes chemoresistance and potentiates antitumor efficacy of 5-FU in colorectal cancer by suppressing proliferation, angiogenesis, and NF-κB/STAT3-regulated proteins. *Cell Death Dis.* **11**, 477 (2020).

27. Koh, S. L. *et al.* Blockade of the renin–angiotensin system inhibits growth of colorectal cancer liver metastases in the regenerating liver. *Clin. Exp. Metastasis* **31**, 395–405 (2014).

28. Riddiough, G. E. *et al.* Captopril, a Renin&ndash;Angiotensin System Inhibitor, Attenuates Tumour Progression in the Regenerating Liver Following Partial Hepatectomy. *International Journal of Molecular Sciences* vol. 23 at https://doi.org/10.3390/ijms23095281 (2022).

29. Vallejo Ardila, D. L. *et al.* Immunomodulatory effects of renin-angiotensin system inhibitors on T lymphocytes in mice with colorectal liver metastases. *J. Immunother. cancer* **8**, (2020).

30. Kochi, T. *et al.* Preventive effects of the angiotensin-converting enzyme inhibitor, captopril, on the development of azoxymethane-induced colonic preneoplastic lesions in diabetic and hypertensive rats. *Oncol. Lett.* **8**, 223–229 (2014).

31. Wen, S. W., Ager, E. I., Neo, J. & Christophi, C. The renin angiotensin system regulates Kupffer cells in colorectal liver metastases. *Cancer Biol. Ther.* **14**, 720–727 (2013).

32. Wysocki, P. J. *et al.* Captopril, an Angiotensin-Converting Enzyme Inhibitor, Promotes Growth of Immunogenic Tumors in Mice. *Clin. Cancer Res.* **12**, 4095–4102 (2006).

33. Li, W. *et al.* Combining losartan with radiotherapy increases tumor control and inhibits lung metastases from a HER2/neu-positive orthotopic breast cancer model. *Radiat. Oncol.* **16**, 48 (2021).

34. Mainetti, L. E. *et al.* Losartan improves the therapeutic effect of metronomic cyclophosphamide in triple negative mammary cancer models. *Oncotarget* **11**, 3048–3060 (2020).

35. Cai, X.-J. *et al.* Candesartan treatment enhances liposome penetration and anti-tumor effect via depletion of tumor stroma and normalization of tumor vessel. *Drug Deliv. Transl. Res.* **11**, 1186–1197 (2021).

36. Takiguchi, T. *et al.* Angiotensin II promotes primary tumor growth and metastasis formation of murine TNBC 4T1 cells through the fibroblasts around cancer cells. *Eur. J. Pharmacol.* **909**, 174415 (2021).

37. Ma, Y. *et al.* AGTR1 promotes lymph node metastasis in breast cancer by upregulating CXCR4/SDF-1α and inducing cell migration and invasion. *Aging (Albany. NY).* **11**, 3969–3992 (2019).

38. Coulson, R. *et al.* The angiotensin receptor blocker, Losartan, inhibits mammary tumor development and progression to invasive carcinoma. *Oncotarget* **8**, 18640–18656 (2017).

39. Xia, T. *et al.* Losartan loaded liposomes improve the antitumor efficacy of liposomal paclitaxel modified with pH sensitive peptides by inhibition of collagen in breast cancer. *Pharm. Dev. Technol.* **23**, 13–21 (2018).

40. Oh, E. *et al.* Overexpression of angiotensin II type 1 receptor in breast cancer cells induces epithelial-mesenchymal transition and promotes tumor growth and angiogenesis. *Biochim. Biophys. Acta* **1863**, 1071–1081 (2016).

41. Rhodes, D. R. *et al.* AGTR1 overexpression defines a subset of breast cancer and confers sensitivity to losartan, an AGTR1 antagonist. *Proc. Natl. Acad. Sci. U. S. A.* **106**, 10284–10289 (2009).

42. Volonte, D., Sedorovitz, M., Cespedes, V. E., Beecher, M. L. & Galbiati, F. Cell autonomous angiotensin II signaling controls the pleiotropic functions of oncogenic K-Ras. *J. Biol. Chem.* **296**, (2021).

43. Tabatabai, E. *et al.* Inhibition of angiotensin II type 1 receptor by candesartan reduces tumor growth and ameliorates fibrosis in colorectal cancer. *EXCLI J.* **20**, 863–878 (2021).

44. Hashemzehi, M. *et al.* Angiotensin receptor blocker Losartan inhibits tumor growth of colorectal cancer. *EXCLI J.* **20**, 506–521 (2021).

45. Dougherty, U. *et al.* Losartan and Vitamin D Inhibit Colonic Tumor Development in a Conditional Apc-Deleted Mouse Model of Sporadic Colon Cancer. *Cancer Prev. Res.* **12**, 433–448 (2019).

46. Asgharzadeh, F. *et al.* Inhibition of angiotensin pathway via valsartan reduces tumor growth in models of colorectal cancer. *Toxicol. Appl. Pharmacol.* **440**, 115951 (2022).

47. Stevens, S. C. W. *et al.* Losartan treatment attenuates tumor-induced myocardial dysfunction. *J. Mol. Cell. Cardiol.* **85**, 37–47 (2015).

48. Ishikane, S. *et al.* Angiotensin II promotes pulmonary metastasis of melanoma through the activation of adhesion molecules in vascular endothelial cells. *Biochem. Pharmacol.* **154**, 136–147 (2018).

49. Otake, A. H. *et al.* Inhibition of angiotensin II receptor 1 limits tumor-associated angiogenesis and attenuates growth of murine melanoma. *Cancer Chemother. Pharmacol.* **66**, 79–87 (2010).

50. Alhusban, A. *et al.* Clinically relevant doses of candesartan inhibit growth of prostate tumor xenografts in vivo through modulation of tumor angiogenesis. *J. Pharmacol. Exp. Ther.* **350**, 635–645 (2014).

51. Takahashi, S. *et al.* Therapeutic targeting of angiotensin II receptor type 1 to regulate androgen receptor in prostate cancer. *Prostate* **72**, 1559–1572 (2012).

52. Leung, W.-H. *et al.* Modulation of NKG2D ligand expression and metastasis in tumors by spironolactone via RXRγ activation. *J. Exp. Med.* **210**, 2675–2692 (2013).

53. Zhou, J. *et al.* Sodium-glucose co-transporter-2 (SGLT-2) inhibition reduces glucose uptake to induce breast cancer cell growth arrest through AMPK/mTOR pathway. *Biomed. Pharmacother.* **132**, 110821 (2020).

54. Nasiri, A. R., Rodrigues, M. R., Li, Z., Leitner, B. P. & Perry, R. J. SGLT2 inhibition slows tumor growth in mice by reversing hyperinsulinemia. *Cancer Metab.* **7**, 10 (2019).

55. Korfhage, J. *et al.* Canagliflozin Increases Intestinal Adenoma Burden in Female ApcMin/+ Mice. *J. Gerontol. A. Biol. Sci. Med. Sci.* **77**, 215–220 (2022).

56. Scafoglio, C. *et al.* Functional expression of sodium-glucose transporters in cancer. *Proc. Natl. Acad. Sci. U. S. A.* **112**, E4111-9 (2015).
