# Supplementary material for: Heart failure pharmacotherapy and cancer: pathways and pre-clinical/clinical evidence
Source: Eur Heart J. 2024 Mar 5;45(14):1224–40. doi: 10.1093/eurheartj/ehae105 (PMC11023004; doi:10.1093/eurheartj/ehae105)
Supplement: ehae105_Supplementary_Data [file ehae105_supplementary_data.zip › Supplementary_table_3_20231211.docx]

| **Effects of BBs on cancer assessed *in vitro*** | | | | | | | |
| --- | --- | --- | --- | --- | --- | --- | --- |
| **Cancer type** | **Study** | **Cell line** | **Treatment** | **Tumor growth measurement** | **Major outcomes by BB** | **Suggested mechanism of action** | **Synergism** |
| **Breast cancer** | **S. Jdeed et al. (2022)**^1^ | MCF10DCIS.com | carvedilol 100 nmol/L for 4 days | fluorescent cell counting with DAPI, clonigenic assay | enhanced antiproliferative effect | normalized ARIDA1A levels | bex +carvedilol combination: markedly stronger inhibition of proliferation |
|  |  | MCF-7 | carvedilol 100 nmol/L for 5 days | fluorescent cell counting with DAPI, clonigenic assay | modestly suppressed growth | enriched ARID1A genomic occupancy at regulatory regions of the IGF-1 pathway | bex + carvedilol combination: markedly stronger inhibition of proliferation |
| **Breast cancer** | **R. D. Gillis et al. (2021)**^2^ | 4T1.2 | carvedilol 10 pM-1 μM for 10 min | cyclic adenosine monophosphate assay | prevents tumour cell invasion | - | isoprenaline+carvedilol combination: antagonised isoprenaline-induced cAMP production, isoprenaline-stimulated MMP2 expression and CRE activation |
|  |  | MDA-MB-231 | carvedilol 10 pM-1 μM for 10 min | cyclic adenosine monophosphate assay | prevents tumour cell invasion | - | isoprenaline+carvedilol combination: antagonised isoprenaline-induced cAMP production, isoprenaline-stimulated MMP2 expression and CRE activation |
| **Breast cancer** | **W-Y. Xie et al. (2019)**^3^ | MDA-MB-231 | propranolol 100, 200 and 400 µM for for  24, 48 and 72 h | Alamar Blue assay, annexin V-FITC apoptosis assay | inhibited cell  viability | increased number of in G0/G1 phase and induced apoptosis | - |
|  |  | MDA-MB-231 | metoprolol 100, 200 and 400 µM for for  24, 48 and 72 h | alamar blue assay, annexin V-FITC apoptosis assay | no notable effect | - | - |
| **Breast cancer** | **Z. Ma et al. (2019)**^4^ | MCF-10A | carvedilol 0.5-10 µmol/l for 48h | determination of intracellular ROS levels, single cell gel electrophoresis (comet assay) | BB was used only in combination with BaP | - | BaP+carvedilol combination: inhibits BaP-induced change in MDM2 and p53 expression levels MDM2, DNA damage and ROS production by PI3K/AKT signaling pathway |
| **Breast cancer** | **A. Montoya et al. (2017)**^5^ | SKBR3 | propranolol 10-200 μM for 48h | alamar blue assay | disrupted cell proliferation | decreased phosphorylation MAPKs and CREB and increased phosphorylation of AKT, GSK3 and p53 | - |
|  |  | SKBR3 | carvedilol 10-200 μM for 48h | alamar blue assay | reduces proliferative index | - | - |
|  |  | SKBR3 | esmolol 10-200 μM for 48h | alamar blue assay | reduces proliferative index | less effective then non-selective β-blockers | esmolol+butaxamine and esmolol+ICI-118,551 combination: failed to produce synergy |
|  |  | SKBR3 | nebivolol 10-200 μM for 48h | alamar blue assay | reduces proliferative index | more effective then selective β-blockers | - |
|  |  | SKBR3 | atenolol 10-200 μM for 48h | alamar blue assay | reduces proliferative index | less effective then non-selective β-blockers | atenolol+butaxamine and atenolol+ICI-118,551 combination: failed to produce synergy |
|  |  | AU565 | propranolol 10-200 μM for 48h | alamar blue assay | reduces proliferative index | - | - |
|  |  | BTS549 | propranolol 10-200 μM for 48h | alamar blue assay | reduces proliferative index | - | - |
|  |  | HCC38 | propranolol 10-200 μM for 48h | alamar blue assay | reduces proliferative index | - | - |
|  |  | HCC70 | propranolol 10-200 μM for 48h | alamar blue assay | reduces proliferative index | - | - |
|  |  | MDA-MB-231 | propranolol 10-200 μM for 48h | alamar blue assay | reduces proliferative index | - | - |
|  |  | MDA-MB-361 | propranolol 10-200 μM for 48h | alamar blue assay | reduces proliferative index | - | - |
|  |  | MDA-MB-175 | propranolol 10-200 μM for 48h | alamar blue assay | reduces proliferative index | - | - |
| **Breast cancer** | **C. Choy et al. (2016)**^6^ | MDA-MB-231 | propranolol 33.3 µM for 5 days | cell counting | decreased the rate of cell proliferation | - | propranolol+terbutaline sulfate combination: eliminated increased proliferation effect |
|  |  | MDA-MB-231Br | propranolol 33.3 µM for 5 days | cell counting | decreased the rate of cell proliferation | - | propranolol+terbutaline sulfate combination: eliminated increased proliferation effect |
| **Breast cancer** | **G. Talarico et al. (2016)**^7^ | MDA-MB-436 | atenolol 500 µM for 48 and 72h | 7-AAD flow cytometry apoptosis assay | not or marginally increased apoptotic frequency | - | - |
|  |  | ZR-75-1 | atenolol 500 µM for 48 and 72h | 7-AAD flow cytometry apoptosis assay | not or marginally increased apoptotic frequency | - | metformin+atenolol combination: mediated activation of AMPK |
| **Breast cancer** | **T. A. D. Smith et al. (2016)**^8^ | SKBR3 | carvedilol 25 ng/ml for 72 h | MTT assay | BB was used only in combination with doxorubicin and with or without trastuzumab | - | doxorubicin+carvedilol and doxorubicin+carvedilol+trastuzumab: not interfere growth-inhibitory effect |
|  |  | BT474 | carvedilol 25 ng/ml for 72 h | MTT assay | BB was used only in combination with doxorubicin and with or without trastuzumab | - | doxorubicin+carvedilol and doxorubicin+carvedilol+trastuzumab: not interfere growth-inhibitory effect |
| **Breast cancer** | **J. M. Wilson et al. (2015)**^9^ | MDA-MB-231 | propranolol 10, 25, 50 μM for 72 hours | thymidine proliferation assay | no significant differences in cell proliferation | - | - |
| **Breast cancer** | **G. Dezong et al. (2014)**^10^ | MCF‑7 | carvedilol 0.1, 1.0, and 5.0 µmol/L for 48h + NE | migration and invasion assay (membrane invasion culture system) | BB was used only in combination with norepinephrine | decreased potential of migration and invasion | norepinephrine+carvedilol combination: inhibited mainly PKCδ-Src pathway also cAMP/PKA-Src pathway |
|  |  | MDA‑MB‑231 | carvedilol 0.1, 1.0, and 5.0 µmol/L for 48h + NE | migration and invasion assay (membrane invasion culture system) | BB was used only in combination with norepinephrine | decreased potential of migration and invasion | norepinephrine+carvedilol combination: inhibited cAMP/PKA-Src pathway |
| **Breast cancer** | **M. Szewczyk et al. (2012)**^11^ | MCF‑7 | bisoprolol for 24h | BrDU assay, LDH assay | low cytotoxic impact and no influenced cell growth+ | - | - |
|  |  | BT20 | bisoprolol for 24h | BrDU assay, LDH assay | low cytotoxic impact with decreased cell growth | - | - |
|  |  | MCF‑7 | propranolol for 24h | BrDU assay, LDH assay | highly cytotoxic impact with strong decrease of cell proliferation | - | - |
|  |  | BT20 | propranolol for 24h | BrDU assay, LDH assay | highly cytotoxic impact with strong decrease of cell proliferation | - | - |
| **Colorectal cancer** | **J. Hu et al. (2021)**^12^ | HCT116 | propranolol (20, 40, 60, 80, 100, 120, and 160 μM; 25, 50, 100, 150, 200, 250, and 300 μM) for 48h | CCK-8 assay | inhibition of proliferation, apoptosis induction | - | T1012G+propranolol combination: enhanced inhibition of cell viability |
|  |  | Widr | propranolol (20, 40, 60, 80, 100, 120, and 160 μM; 25, 50, 100, 150, 200, 250, and 300 μM) for 48h | CCK-8 assay | inhibition of proliferation, apoptosis induction | - | T1012G+propranolol combination: enhanced inhibition of cell viability |
|  |  | MC38 | propranolol (20, 40, 60, 80, 100, 120, and 160 μM; 25, 50, 100, 150, 200, 250, and 300 μM) for 48h | CCK-8 assay | inhibition of proliferation, apoptosis induction | - | T1012G+propranolol combination: enhanced inhibition of cell viability |
|  |  | CT26WT | propranolol (20, 40, 60, 80, 100, 120, and 160 μM; 25, 50, 100, 150, 200, 250, and 300 μM) for 48h | CCK-8 assay | inhibition of proliferation, apoptosis induction | - | T1012G+propranolol combination: enhanced inhibition of cell viability |
| **Colorectal cancer** | **M. Coelho et al. (2015)**^13^ | HT-29 | propranolol 0.1-100 μM for for 12 or 24h | MTS assay, MTT assay | decreased cell proliferation | - | adrenaline+propranolol combination: reduced AD-induced cell proliferation, isoprenalin+propranolol combination: decreased cell proliferation stimulated by ISO |
|  |  | HT-29 | carvedilol 0.1-100 μM for for 12 or 24h | MTS assay, MTT assay | no significant decrease in cell proliferation | - | carvedilol+adrenaline and carvedilol+isoprenaline combination: reversing the proliferative effects of AD and ISO |
|  |  | HT-29 | atenolol 0.1-100 μM for for 12 or 24h | MTS assay, MTT assay | decreased cell proliferation | - | atenolol+adrenaline and atenolol+isoprenaline combination: blocked AD- and ISO-induced cell proliferation |
| **Lung cancer** | **M. Sidorova et al. (2022)**^14^ | A549 | atenolol 500 µM for 72h | MTT assay, colony formation assay | reduced cell viability | induced apoptosis | - |
|  |  | H1299 | atenolol 500 µM for 72h | MTT assay, colony formation assay | reduced cell viability, no reduction in number of colonies | induced both apoptosis and necrosis | - |
|  |  | A549 | betaxolol 500 µM for 72h | MTT assay, colony formation assay | strongly reduced cell viability, strongest completely suppressed colony formation ability | induced apoptosis | - |
|  |  | H1299 | betaxolol 500 µM for 72h | MTT assay, colony formation assay | strongly reduced cell viability, completely suppressed colony formation ability | induced both apoptosis and necrosis | - |
|  |  | A549 | esmolol 500 µM for 72h | MTT assay, colony formation assay | reduced cell viability, slightly stronger inhibited growth of cell colonies | induced apoptosis | - |
|  |  | H1299 | esmolol 500 µM for 72h | MTT assay, colony formation assay | reduced cell viability, slightly stronger inhibited growth of cell colonies | induced both apoptosis and necrosis | - |
|  |  | A549 | metoprolol 500 µM for 72h | MTT assay, colony formation assay | slightly stronger reduced cell viability, slightly stronger inhibited growth of cell colonies | induced apoptosis | - |
|  |  | H1299 | metoprolol 500 µM for 72h | MTT assay, colony formation assay | reduced cell viability, slightly stronger inhibited growth of cell colonies | induced both apoptosis and necrosis | - |
|  |  | A549 | pindolol 500 µM for 72h | MTT assay, colony formation assay | reduced cell viability, inhibited growth of cell colonies | induced apoptosis | - |
|  |  | H1299 | pindolol 500 µM for 72h | MTT assay, colony formation assay | reduced cell viability, inhibited growth of cell colonies | induced both apoptosis and necrosis | - |
|  |  | A549 | propranolol 500 µM for 72h | MTT assay, colony formation assay | strongly reduced cell viability, completely suppressed colony formation ability | induced apoptosis | - |
|  |  | H1299 | propranolol 500 µM for 72h | MTT assay, colony formation assay | most reduced cell viability, completely suppressed colony formation ability | induced both apoptosis and necrosis | - |
|  |  | A549 | timolol 500 µM for 72h | MTT assay, colony formation assay | reduced cell viability, inhibited growth of cell colonies | induced apoptosis | - |
|  |  | H1299 | timolol 500 µM for 72h | MTT assay, colony formation assay | reduced cell viability, inhibited growth of cell colonies | induced both apoptosis and necrosis | - |
| **Lung cancer** | **M. Niu et al. (2021)**^15^ | H1975 | nebivolol | MTS assay | strongly inhibited cell viability | upregulated FBXL2 and downregulated EGFR expression | - |
|  |  | PC-9 | nebivolol 10 µM for 48h | MTS assay | strongly inhibited cell viability | upregulated FBXL2 and downregulated EGFR expression | - |
| **Lung cancer** | **K. R. Chaudhary et al. (2019)**^16^ | PC-9 | propranolol 50 μM + radiation | clonigenic assay | decreased clonogenic survival | downregulates p-PKA, sensitizes to direct cytotoxic effects of radiation | propranolol+cisplatin combination: further decrease of clonogenic survival |
|  |  | A549 | propranolol 50 μM + radiation | clonigenic assay | decreased clonogenic survival | downregulates p-PKA, sensitizes to direct cytotoxic effects of radiation | propranolol+cisplatin combination: further decrease of clonogenic survival |
|  |  | PC9 | propranolol 1 μM + radiation | clonigenic assay | no significant impact on clonogenic survival | - | - |
|  |  | A549 | propranolol 1 μM + radiation | clonigenic assay | no significant impact on clonogenic survival | - | - |
| **Lung cancer** | **M. B. Nilsson et al. (2017)**^17^ | HCC827 | propranolol 1 μM one hour before NE + erlotinib for 24h | MTS assay | BB was used only in combination with norepinephrine | blocked effect on EGFR TKI resistance | norepinephrine+propranolol combination: abrogated NE-induced inactivation of LKB1 and blocked IL-6 |
| **Lung cancer** | **A. Chang et al. (2015)**^18^ | A549 | carvedilol 1, 10, 30 μM | anchorage-independent growth assay in soft agar, SRB assay | inhibited colony formation at high concentrations | decreased viability and cell mobility | - |
| **Lung cancer** | **G-H. Deng et al. (2014)**^19^ | A549 | propranolol 10 μM 30 minutes before adding NE for 6h | ELISA | blocked NE-induced upregulation of VEGF, IL-8, and IL-6 protein levels | - | - |
| **Melanoma** | **L. S. Farhoumand et al. (2022)**^20^ | Mel270 | sotalol for 7days | spheroid viability assay (ATP luminescence assay) | no effect on spheroid viability | - | - |
|  |  | Mel270 | timolol for 7days | spheroid viability assay (ATP luminescence assay) | no effect on spheroid viability | - | - |
|  |  | Mel270 | pindolol for 7days | spheroid viability assay (ATP luminescence assay) | no effect on spheroid viability | - | - |
|  |  | Mel270 | propranolol 150 µM for 7days | spheroid viability assay (ATP luminescence assay) | decreased spheroid viability in a concentration-dependent manner | - | - |
|  |  | Mel270 | labetolol 150 µM for 7days | spheroid viability assay (ATP luminescence assay) | decreased spheroid viability in a concentration-dependent manner | main anti-tumor mechanism due to α receptor blocking | - |
|  |  | Mel270 | carvedilolol 10-50 µM µM for 48h and 7days | spheroid viability assay (ATP luminescence assay), cell viability assay | most potently decreased spheroid viability in a concentration-dependent manner | main anti-tumor mechanism due to α receptor blocking by inducing apoptotic pathways | - |
|  |  | 92-1 | carvedilolol 10-50 µM for 7days | spheroid viability assay (ATP luminescence assay) | reduced spheroid viability | higher concectration needed because of lower penetration of larger tumors | - |
|  |  | UPMD2 | carvedilolol 10-50 µM µM for 7days | spheroid viability assay (ATP luminescence assay) | blocked spheroid viability | - | - |
|  |  | UPMM3 | carvedilolol 10–50 µM for 7days | spheroid viability assay (ATP luminescence assay) | most blocked viability | - | carvedilol+radiation combination: completely blocked repopulation of spreading spheroid |
| **Melanoma** | **P. Bustamante (2019)**^21^ | MEL270 | propranolol 12.5-200 μL for 24h | trypan blue exclusion assay, CCK-8 assay | decreased cell viability in a dose‐dependent manner | reduction in metabolic activity by reduced dehydrogenase activity by 50%, induction of apoptosis and cell cycle arrest (TUNEL assay) | - |
|  |  | OMM2.5 | propranolol 12.5-200 μL for 24h | trypan blue exclusion assay, CCK-8 assay | decreased cell viability in a dose‐dependent manner | reduction in metabolic activity by reduced dehydrogenase activity by 50% | - |
|  |  | MP41 | propranolol 12.5-200 μL for 24h | trypan blue exclusion assay, CCK-8 assay | decreased cell viability in a dose‐dependent manner | reduction in metabolic activity by reduced dehydrogenase activity by 50%, induction of apoptosis and cell cycle arrest (TUNEL assay) | - |
|  |  | MP46 | propranolol 12.5-200 μL for 24h | trypan blue exclusion assay, CCK-8 assay | decreased cell viability in a dose‐dependent manner | reduction in metabolic activity by reduced dehydrogenase activity by 50% | - |
|  |  | WM266.4 | propranolol 12.5-200 μL for 24h | trypan blue exclusion assay, CCK-8 assay | decreased cell viability in a dose‐dependent manner | reduction in metabolic activity by reduced dehydrogenase activity by 50%, induction of apoptosis and cell cycle arrest (TUNEL assay) | - |
| **Melanoma** | **S. Maccari et al. (2017)**^22^ | B16F10 | propranolol 0,01-10 μM for 24, 48 and 72 h | MTS assay | does not affect cell proliferation | - | - |
| **Melanoma** | **C. Zhou et al. (2016)**^23^ | A375 | propranolol 25-400 μM for 24h, 48h and 72h | alamar blue assay | reduced cell proliferation | induced cell cycle arrest, activated mitochondria-mediated apoptosis pathway and inhibited MAPK pathway | - |
|  |  | P-3 (acral human patient) | propranolol 25-400 μM for 24h, 48h and 72h | alamar blue assay | reduced cell proliferation | - | - |
| **Prostate cancer** | **D. Palm et al. (2006)**^24^ | PC-3-luc cells | propranolol 10 μM for 4 days | cell counting | no measurable influence on the proliferation | - | propranolol+norepinephrine combination: inhibited NE-induced increase of migratory activity |

| **Effects of ACEIs on cancer assessed *in vitro*** | | | | | | | |
| --- | --- | --- | --- | --- | --- | --- | --- |
| **Cancer type** | **Study** | **Cell line** | **Treatment** | **Tumor growth measurement** | **Major outcomes by BB** | **Suggested mechanism of action** | **Synergism** |
| **Breast cancer** | **F. Rasha et al. (2020)**^25^ | MCF-7 | captopril 100 μM for 24–72h | MTT assay | did not alter markers of cancer cell growth | - | - |
|  |  | MDA-MB-231 | captopril 100 μM for 24–72h | MTT assay | did not alter markers of cancer cell growth | reduced markers of inflammation | - |
| **Breast cancer** | **T. A. D. Smith at el. (2016)**^8^ | SKBR3 | enalapril 5 μM, 25 ng/ml, 250 ng/ml and 5 μg/ml for 72h | MTT assay | ACEI was used only in combination with doxorubicin and with or without trastuzumab | - | doxorubicin+enalapril and doxorubicin+trastuzumab+enalapril combination: did not interfere with the growth-inhibitory effect of doxorubicin alone nor in combination with trastuzumab |
|  |  | BT474 | enalapril 5 μM, 25 ng/ml, 250 ng/ml and 5 μg/ml for 72h | MTT assay | ACEI was used only in combination with doxorubicin and with or without trastuzumab | - | doxorubicin+enalapril and doxorubicin+trastuzumab+enalapril combination: did not interfere with the growth-inhibitory effect of doxorubicin alone nor in combination with trastuzumab |
| **Breast cancer** | **S. Namazi et al. (2014)**^26^ | MCF-7 | medium containing either 100 μmol/L captopril + 1 μmol/L TAM for 96h | MTT assay | reduced number of viable | - | captopril+losartan combination: increased inhibitory effect of TAM |
|  |  | TAM-R | medium containing either 100 μmol/L captopril + 1 μmol/L TAM | MTT assay | MTT could not be performed | led to cell death | captopril+losartan combination: led to cell death |
| **Breast cancer** | **E. Napoleone et al. (2012)**^27^ | MDA-MB-231 | captopril 10 μg/ml for 6h | MTT assay | no cytotoxic effect | dose-dependent inhibition of TF activity | - |
| **Breast cancer** | **R. E. Brown et al. (2004)**^28^ | SKBR-3 | captopril 3 and 9 mM for 4days | MTS assay | inhibitory effects on cell growth in a dose-dependant manner | decrease in p-ERK1/2 and p-JNK | - |
|  |  | MDA-175 | captopril 3 and 9 mM for 4days | MTS assay | strongest inhibitory effects on cell growth in a dose-dependant manner | decrease in p-Akt and an increase in p-ERK1/2 and p-JNK | - |
|  |  | MDA-231 | captopril 3 and 9 mM for 4days | MTS assay | inhibitory effects on cell growth in a dose-dependant manner | decrease in p-JNK | - |
| **Colorectal cancer** | **Y. Yang et al. (2020)**^29^ | SW620 | enalapril 0-2000 μM for 48 or 72 h | MTT assay, Annexin V/PI apopotosis assay | not significantly affect cell viability | no significant effect on cell apoptosis | 5-FU+enalaprilcombination: extremely decreased cell viability and more profound apoptosis |
|  |  | HCT116 | enalapril 0-2000 μM for 48 or 72 h | MTT assay, Annexin V/PI apopotosis assay | not significantly affect cell viability | no significant effect on cell apoptosis | 5-FU+enalaprilcombination: extremely decreased cell viability and more profound apoptosis |
|  |  | P1 (median 5-FU-resistant) | enalapril 0-2000 μM for 48 or 72 h | MTT assay, Annexin V/PI apopotosis assay | - | - | 5-FU+enalaprilcombination: extremely suppressed cell growth |
|  |  | P2 (5-FU-resistant) | enalapril 0-2000 μM for 48 or 72 h | MTT assay, Annexin V/PI apopotosis assay | - | - | 5-FU+enalaprilcombination: extremely suppressed cell growth |
|  |  | P3 (5-FU-resistant) | enalapril 0-2000 μM for 48 or 72 h | MTT assay, Annexin V/PI apopotosis assay | - | - | 5-FU+enalaprilcombination: extremely suppressed cell growth |
| **Colorectal cancer** | **Y. Lu et al. (2019)**^30^ | HT-29 | S-nitrosocaptopril 0.1-1000 μM for 24h | MTT assay | no statistically significant changes in cell viability | indicating that IC50 was greater than 1000 μM | - |
| **Colorectal cancer** | **Y. Lu et al. (2014)**^31^ | HT-29 | S-nitrosocaptopril 0.1-1000 μM for 24h | MTT assay | could not reach the inhibitory IC50 values | indicating that the IC50 was greater than 1000 μM | - |
| **Melanoma** | **Y. Lu et al. (2019)**^30^ | B16F10 | S-nitrosocaptopril 0.1-1000 μM for 24h | MTT assay | no statistically significant changes in cell viability | indicating that IC50 was greater than 1000 μM | - |

| **Effects of ARBs on cancer assessed *in vitro*** | | | | | | | |
| --- | --- | --- | --- | --- | --- | --- | --- |
| **Cancer type** | **Study** | **Cell line** | **Treatment** | **Tumor growth measurement** | **Major outcomes by BB** | **Suggested mechanism of action** | **Synergism** |
| **Breast cancer** | **F. Rasha et al. (2020)**^25^ | MCF-7 | telmisartan 10 μM for 24-72h | MTT assay | ARB was used only in combination with angiotensin2 | - | angiotensin2+telmisartan combination: blocked Ang2 effects by reducing IL-6 secretion |
|  |  | MDA-MB-231 | telmisartan 10 μM for 24-72h | MTT assay | ARB was used only in combination with angiotensin2 | - | angiotensin2+telmisartan combination: blocked Ang2 effects by reducing IL-6 secretion |
| **Breast cancer** | **S. Ni et al. (2020)**^32^ | T-47D | candesartan cilexetic 5 μM, 10 μM, 15 μM, and 20 μM for 48 h or 10 days | cell proliferation (CCK-8 assay) and cell clonogenic assay | decreased proliferation | inhibiting effect related to unique structure and irrelevant to A2T1R antagonistic effect | - |
|  |  | MCF-7 | candesartan cilexetic 5 μM, 10 μM, 15 μM, and 20 μM for 48 h or 10 days | cell proliferation (CCK-8 assay) and cell clonogenic assay, apoptosis assay (Annexin-V/PI assay) | decreased proliferation | inhibiting effect related to unique structure and irrelevant to A2T1R antagonistic effect | - |
| **Breast cancer** | **M. A. Redondo-Müller et al. (2008)**^33^ | MCF7 | losartan 0.005-25 μM for 48h | XTT assay | no inhibitory effect | - | - |
|  |  | MDA-MB-231 | losartan 0.005-25 μM for 48h | XTT assay | no inhibitory effect | - | - |
|  |  | T47D | losartan 0.005-25 μM for 48h | XTT assay | 43% inhibition at 25 μM | - | - |
| **Breast cancer** | **S. Namazi et al. (2014)**^26^ | MCF-7 | losartan 10 μM | MTT assay | reversal of tamoxifen resistance in combination with captopril | - | captopril+losartan+tamoxifen combination: led to cell death and reversal of tamoxifen resistance |
| **Breast cancer** | **N. Du et al. (2012)**^34^ | MCF-7 | irbesartan 0.1-100 µM for 30 min before Ang2 for 24h | MTT assay | inhibition of angiotensin II mediated proliferation | greatest  inhibition of Ang2-mediated cell  proliferation in a dose-dependent manner | angiotensin2+irbesartan combination: suppression of ANG II effects on proliferation and cell cycle |
|  |  | MCF-7 | losartan 0.1-100 µM for 30 min before Ang2 for 24h | MTT assay | inhibition of angiotensin II mediated proliferation | inhibited Ang2-mediated cell  proliferation in a dose-dependent manner | angiotensin2+losartan combination: suppression of ANG II effects on proliferation and cell cycle |
|  |  | MCF-7 | valsartan 0.1-100 µM for 30 min before Ang2 for 24h | MTT assay | inhibition of angiotensin II mediated proliferation | inhibited Ang2-mediated cell  proliferation in a dose-dependent manner | angiotensin2+valsartan combination: suppression of ANG II effects on proliferation and cell cycle |
|  |  | MCF-7 | candesartan 0.1-100 µM for 30 min before Ang2 for 24h | MTT assay | inhibition of angiotensin II mediated proliferation | inhibited Ang2-mediated cell  proliferation in a dose-dependent manner | angiotensin2+candesartan combination: suppression of ANG II effects on proliferation and cell cycle |
| **Colorectal cancer** | **F. Asgharzadeh et al. (2022)**^35^ | CT26 | valsartan 1 nM-10 mM for 24h | MTT assay, cell scratch assay | dose-dependently inhibited cell proliferation and migration | modulates ROS formation and oxidative stress | 5-FU+valsartan combination: decreased the IC50 value of 5-FU |
| **Colorectal cancer** | **E. Tabatabai et al. (2021)**^36^ | CT-26 | candesartan 0-1000 µM for 24h | growth inhibition (MTT) assay | inhibited cell viability in dose-dependent manner | induced cell death, decreased MMP-3 and MMP-9 expression and increased E-cadherin expression | - |
|  |  | SW-480 | candesartan 0-1000 µM for 24h | growth inhibition (MTT) assay | inhibited cell viability in dose-dependent manner | induced cell death, decreased MMP-3 and MMP-9 expression and increased E-cadherin expression | - |
| **Colorectal cancer** | **M. Hashemzehi et al. (2021)**^37^ | CT-26 | Losartan 0-1000 μM for 24h | MTT assay | decreased cell viability in a concentration-dependent manner, decreased spheroid size and induced tumor shrinkage | induced cell toxicity and apoptosis by upregulating mRNA levels of key pro-apoptotic genes including P53 and BAX, downregulated PI3K, AKT and cyclin D1 expression in a time-dependent manner | - |
| **Lung cancer** | **S. Ni et al. (2020)**^32^ | A549 | candesartan cilexetic 5 μM, 10 μM, 15 μM, and 20 μM for 48 h or 10 days | CCK-8 assay, cell clonogenic assay | inhibited cell proliferation and clonogenic survival in a dose-dependent manner | neddylation inhibition, induced cancer cell apoptosis by increasing total numbers of early and late apoptotic and cleaved-PARP | - |
|  |  | EKVX | candesartan cilexetic 5 μM, 10 μM, 15 μM, and 20 μM for 48 h or 10 days | CCK-8 assay, cell clonogenic assay | decreased proliferation | inhibiting effect related to unique structure and irrelevant to A2T1R antagonistic effect | - |
|  |  | H1299 | candesartan cilexetic 5 μM, 10 μM, 15 μM, and 20 μM for 48 h or 10 days | CCK-8 assay, cell clonogenic assay | decreased proliferation | inhibiting effect related to unique structure and irrelevant to A2T1R antagonistic effect | - |
| **Lung cancer** | **V. R. Martínez et al. (2018)**^38^ | A549 | losartan 2.5, 5, 10, 25, 50, 75, 100, 250, and 500 μM for 24 h | MTT assay | no siginificant effect | - | ZnLos combination: declined cell proliferation in a dose-dependent manner |
| **Lung cancer** | **M. Rasheduzzaman et al. (2018)**^39^ | A549 | candesartan 2.5, 5, 10 μM for 12h | cell viability test, Annexin V assay | ARB was used only in combination with TRAIL | - | TRAIL+candesartan combination: increased observation of apoptotic cell morphologies, induced apoptosis in a dose-dependent manner, decreased cell viability |
|  |  | HCC-15 | candesartan 2.5, 5, 10 μM for 12h | cell viability test, Annexin V assay | ARB was used only in combination with TRAIL | - | TRAIL+candesartan combination: increased observation of apoptotic cell morphologies, induced apoptosis in a dose-dependent manner, decreased cell viability |
| **Melanoma** | **D. N. Olschewski et al. (2018)**^40^ | MV3 | losartan 0.7 µmol/l for 24h | cell counting | no significant effect | - | ATII+losartan combination: no significant effect |
| **Prostate cancer** | **Y. Woo et al. (2017)**^41^ | PC3 | fimasartan 100, 200 and 400 µM for 48 and 72h | WST-1 assay | reduced cell viability with greatest cytotoxicity | induced autophagy by increased LC3-II ecxpression, induced anti-migratory activity | - |
|  |  | DU145 | fimasartan 100, 200 and 400 µM for 48 and 72h | WST-1 assay | reduced cell viability with greatest cytotoxicity | induced anti-migratory activity | - |
|  |  | LNCap-LN3 | fimasartan 100, 200 and 400 µM for 48 and 72h | WST-1 assay | reduced cell viability with greatest cytotoxicity | - | - |
|  |  | PC3 | losartan 100, 200 and 400 µM for 48 and 72h | WST-1 assay | reduced cell viability | induced autophagy by increased LC3-II ecxpression | - |
|  |  | DU145 | losartan 100, 200 and 400 µM for 48 and 72h | WST-1 assay | reduced cell viability | - | - |
|  |  | LNCap-LN3 | losartan 100, 200 and 400 µM for 48 and 72h | WST-1 assay | reduced cell viability | - | - |
|  |  | PC3 | eprosartan 100, 200 and 400 µM for 48 and 72h | WST-1 assay | reduced cell viability | induced autophagy by increased LC3-II ecxpression | - |
|  |  | DU145 | eprosartan 100, 200 and 400 µM for 48 and 72h | WST-1 assay | reduced cell viability | - | - |
|  |  | LNCap-LN3 | eprosartan 100, 200 and 400 µM for 48 and 72h | WST-1 assay | reduced cell viability | - | - |
|  |  | PC3 | valsartan 100, 200 and 400 µM for 48 and 72h | WST-1 assay | reduced cell viability withlowest anti-proliferative activity | induced autophagy by increased LC3-II ecxpression | - |
|  |  | DU145 | valsartan 100, 200 and 400 µM for 48 and 72h | WST-1 assay | reduced cell viability withlowest anti-proliferative activity | - | - |
|  |  | LNCap-LN3 | valsartan 100, 200 and 400 µM for 48 and 72h | WST-1 assay | reduced cell viability withlowest anti-proliferative activity | - | - |
| **Prostate cancer** | **M. S. Islas et al. (2016)**^42^ | LNCaP | irbesartan 25, 50 and 100 μM for 24h | MTT assay | decreased cell viability | - | - |
|  |  | DU145 | irbesartan 25, 50 and 100 μM for 24h | MTT assay | decreased cell viability | - | - |
| **Prostate cancer** | **A. Alhusban et al. (2014)**^43^ | PC3 | candesartan 0.5, 5, 10, 25, and 200 µM for 24h and 72h | BrdU assay, MTT assay, ELISA-based apoptosis assay | induced dose-dependent antiapoptotic effect, did not have effect on the proliferation or viability | inhibited VEGF mRNA expression | - |
|  |  | DU145 | candesartan 0.5, 5, 10, 25, and 200 µM for 24h and 72h | na | na | inhibited VEGF mRNA expression | - |
| **Prostate cancer** | **Y-J. Da et al. (2012)**^44^ | LNCap | losartan 10 μM for 24h | MTT assay | no significant effect | - | angiotensin2+losartan combination: no significant effect |
| **Prostate cancer** | **J-i. Teranishi et al. (2008)**^45^ | LNCaP | olmesartan 10 µM for 30 min before Ang3 treatment for 5 days | cell counter | ARB was used only in combination with angiotensin3 | suppressed cell growth induced by Ang3 treatment | angiotensin3+olmesartan combination: inhibited phosphorylation of MAPK activated by Ang3 |
|  |  | DU145 | olmesartan 10 µM for 30 min before AngIII treatment for 5 days | cell counter | ARB was used only in combination with angiotensin3 | suppressed cell growth induced by Ang3 treatment | angiotensin3+olmesartan combination: inhibited phosphorylation of MAPK activated by Ang3 |
| **Prostate cancer** | **H. Ishiguro et al. (2007)**^46^ | LNCaP | telmisartan 1 and 10 mM for 30 min before Ang2 treatment | MTT assay | inhibited cell growth | - | DHT+telmisartan combination: downregulated PSA expression |
|  |  | DU145 | telmisartan 1 and 10 mM for 30 min before Ang2 treatment | MTT assay | inhibited cell growth | attenuated phosphorylation of MAPK | DHT+telmisartan combination: downregulated PSA expression, GW9662+telmisartan combination: cell growth was also inhibited |
| **Prostate cancer** | **H. Uemura et al. (2005**^47^**)** | PC-3 | losartan 10 µM for 30 min before Ang2 or EGF treatment for 5 days | cell growth analysis | no significant effect | - | - |

| **Effects of MRAs on cancer assessed *in vitro*** | | | | | | | |
| --- | --- | --- | --- | --- | --- | --- | --- |
| **Cancer type** | **Study** | **Cell line** | **Treatment** | **Tumor growth measurement** | **Major outcomes by MRAs** | **Suggested mechanism of action** | **Synergism** |
| **Colorectal cancer** | **W-H. Leung et al. (2013)**^49^ | HCT116 | spironolactone 56 μM for 24h | comet assay | decrease in DNA breakage | induced ATM–ATR pathways and NKG2DL expression requiring RXRγ activation | - |
|  |  | HCT116 | spironolactone 56 μM for 5 days | cytotoxicity assay | not affecting cell viability | upregulated NKG2DLs, increased ULBP2 expression in a dose-dependent manner | - |
|  |  | SW480 | spironolactone 56 μM for 5 days | cytotoxicity assay | not affecting cell viability | increased ULBP2 and ULBP1 expression in a dose-dependent manner, enhanced primary NK cell-mediated lysis | - |
|  |  | HT29 | spironolactone 56 μM for 5 days | cytotoxicity assay | not affecting cell viability | increased ULBP2 expression in a dose-dependent manner, enhanced primary NK cell-mediated lysis | - |
|  |  | HCT15 | spironolactone 56 μM for 5 days | cytotoxicity assay | not affecting cell viability | increased ULBP2 and ULBP1 expression in a dose-dependent manner | - |
| **Lung cancer** | **T. Sanomachi et al. (2019)**^50^ | A549 | spironolactone 25, 50 and 100 μM for 3 days | Trypan blue exclusion assay | induced cell death and inhibited cell growth | - | gemcitabine+spironolactone and osimertinib+spironolactone combination: spironolactone reversed resistance |
|  |  | PC-9 | spironolactone 25, 50 and 100 μM for 3 days | Trypan blue exclusion assay | induced cell death and inhibited cell growth | - | gemcitabine+spironolactone and osimertinib+spironolactone combination: spironolactone reversed resistance |
|  |  | PC-9-(osimertinib resistant) | spironolactone 25, 50 and 100 μM for 3 days | Trypan blue exclusion assay | induced cell death and inhibited cell growth | - | gemcitabine+spironolactone and osimertinib+spironolactone combination: spironolactone reversed resistance |
|  |  | A549 cancer stem cell line | spironolactone 25, 50 and 100 μM for 3 days | Trypan blue exclusion assay | induced cell death and inhibited cell growth | - | - |
| **Melanoma** | **S. Sayedyahossein et al. (2021)**^51^ | 131/4-5B1 | spironolactone 10 μM for 72h | immunoprecipitation | reduced cytoplasmic levels of both PANX1 and β-catenin | - | - |
| **Prostate cancer** | **A. Dovio et al. (2009)**^48^ | LNCaP | eplerenone 1 μM for 24h | na | blocking cortisol’s inhibitiory effect on IL-1β-inducible osteoprtegerin release | - | cortisol+eplerenone combination: completely reverted the effect of cortisol |

| **Effects of SGLT2Is on cancer assessed *in vitro*** | | | | | | | |
| --- | --- | --- | --- | --- | --- | --- | --- |
| **Cancer type** | **Study** | **Cell line** | **Treatment** | **Tumor growth measurement** | **Major outcomes by MRAs** | **Suggested mechanism of action** | **Synergism** |
| **Breast cancer** | **D. Papadopoli et al. (2021)**^52^ | SKBR3 | canagliflozin 50 µM for 24h | automated TC10 cell counting, trypan blue exclusion assay | inhibited cell proliferation | interfere with glutamine-mediated anaplerosis and inhibits glutamine metabolism largely independent of SGLT2 inhibition | - |
|  |  | BT474 | canagliflozin 50 µM for 24h | automated TC10 cell counting, trypan blue exclusion assay | inhibited cell proliferation | largely independent of SGLT2 inhibition | - |
|  |  | NT2196 | canagliflozin 50 µM for 24h | automated TC10 cell counting, trypan blue exclusion assay | modest suppression of cell proliferation | - | - |
|  |  | NT2197 | canagliflozin 50 µM for 24h | automated TC10 cell counting, trypan blue exclusion assay | modest suppression of cell proliferation | - | - |
|  |  | SKBR3 | dapagliflozin 50 µM for 24h | automated TC10 cell counting, trypan blue exclusion assay | inhibited cell proliferation | largely independent of SGLT2 inhibition | - |
|  |  | BT474 | dapagliflozin 50 µM for 24h | automated TC10 cell counting, trypan blue exclusion assay | inhibited cell proliferation | - | - |
| **Breast cancer** | **S. G. Eliaa et al. (2020)**^53^ | MDA-MB-231 | empagliflozin 0,1-100 uM/l for 24 h | MTT assay, Annexin V-FITC apoptosis assay | inhibited cell growth, increase in the percent of apoptotic | clear decline in the cell population at the S phase, interfering mTOR pathway and inhibited calmodulin | doxorubicin+empagliflozin combination: greater cell growth inhibition in a dose-dependent manner |
| **Breast cancer** | **V. Quagliariello et al. (2020)**^54^ | MCF-7 | empagliflozin 500 nM for 72h | MTT assay, Annexin V-FITC apopotosis assay | SGLT2I was used only in combination with ipilimumab or anti CTLA-4 antibody | - | ipilimumab+digoxin combination: ameliorated cell responsiveness to ipilimumab, anti CTLA-4+digoxin combination: increased anticancer efficacy of anti CTLA-4 antibody |
|  |  | MDA-MB-231 | empagliflozin 500 nM for 72h | MTT assay, Annexin V-FITC apopotosis assay | SGLT2I was used only in combination with ipilimumab or anti CTLA-4 antibody | - | ipilimumab+digoxin combination: ameliorated cell responsiveness to ipilimumab, anti CTLA-4+digoxin combination: increased anticancer efficacy of anti CTLA-4 antibody |
| **Breast cancer** | **J. Zhou et al. (2020)**^55^ | MCF-7 | dapagliflozin 3.67, 11, 33, 100, 300 μM for 24h and72h | MTT assay, colony formation assay | blocked cell proliferation and growth and inhibit the clonogenic survival | induced G1/G0 cell cycle arrest, inhibition of mTOR pathway via AMPK activation | - |
|  |  | MCF-7 | canagliflozin 3.67, 11, 33, 100, 300 μM for 72h | MTT assay | blocked cell proliferation | inhibition of mTOR pathway via AMPK activation | - |
|  |  | MCF-7 | canagliflozin 40 µM for 48h | MTT assay | SGLT2I was used only in combination with doxorubicin | - | doxorubicin+canagliflozin combination: increased cytotoxic activity of DOX |
| **Breast cancer** | **S. Komatsu et al. (2020)**^56^ | MCF-7 | ipragliflozin 0-50 μM for 0-4 days | manual cell counting, BrDU incorporation assay | decreased cell number in a dose-dependent manner | through SGLT2 inhibition, inhibited DNA synthesis | - |
| **Lung cancer** | **L. Yamamoto et al. (2021)**^57^ | A549 | canagliflozin 1-50 μM for 0-3 days | manual cell counting, BrDU incorporation assay | decreased cell number in a dose-dependent manner | inhibited DNA synthesis in a dose-dependent manner, attenuated cell cycle progression | - |
|  |  | H520 | canagliflozin 1-50 μM for 0-3 days | manual cell counting, BrDU incorporation assay | attenuated cell proliferation | - | - |
|  |  | H1975 | canagliflozin 1-50 μM for 0-3 days | manual cell counting, BrDU incorporation assay | attenuated cell proliferation | - | - |

**Supplementary table 3.:** *In vitro* studies investigating the effect of guideline-directed HF pharmacotherapies on cancer.

**References:**

1. Jdeed, S., Erdős, E., Bálint, B. L. & Uray, I. P. The Role of ARID1A in the Nonestrogenic Modulation of IGF-1 Signaling. *Mol. Cancer Res.* **20**, 1071–1082 (2022).

2. Gillis, R. D. *et al.* Carvedilol blocks neural regulation of breast cancer progression in vivo and is associated with reduced breast cancer mortality in patients. *Eur. J. Cancer* **147**, 106–116 (2021).

3. Xie, W.-Y. *et al.* β‑blockers inhibit the viability of breast cancer by regulating the ERK/COX‑2 signaling pathway and the drug response is affected by ADRB2 single‑nucleotide polymorphisms. *Oncol. Rep.* **41**, 341–350 (2019).

4. Ma, Z., Liu, X., Zhang, Q., Yu, Z. & Gao, D. Carvedilol suppresses malignant proliferation of mammary epithelial through inhibition of the ROS‑mediated PI3K/AKT signaling pathway. *Oncol. Rep.* **41**, 811–818 (2019).

5. Montoya, A. *et al.* Use of non-selective β-blockers is associated with decreased tumor proliferative indices in early stage breast cancer. *Oncotarget* **8**, 6446–6460 (2017).

6. Choy, C. *et al.* Inhibition of β2-adrenergic receptor reduces triple-negative breast cancer brain metastases: The potential benefit of perioperative β-blockade. *Oncol. Rep.* **35**, 3135–3142 (2016).

7. Talarico, G. *et al.* Aspirin and atenolol enhance metformin activity against breast cancer by targeting both neoplastic and microenvironment. *Sci. Rep.* **6**, 18673 (2016).

8. Smith, T. A. D., Phyu, S. M. & Akabuogu, E. U. Effects of Administered Cardioprotective Drugs on Treatment Response of Breast Cancer. *Anticancer Res.* **36**, 87–93 (2016).

9. Wilson, J. M., Lorimer, E., Tyburski, M. D. & Williams, C. L. β-Adrenergic receptors suppress Rap1B prenylation and promote the metastatic phenotype in breast cancer. *Cancer Biol. Ther.* **16**, 1364–1374 (2015).

10. Dezong, G., Zhongbing, M., Qinye, F. & Zhigang, Y. Carvedilol suppresses migration and invasion of malignant breast by inactivating Src involving cAMP/PKA and PKCδ signaling pathway. *J. Cancer Res. Ther.* **10**, 998–1003 (2014).

11. Szewczyk, M., Richter, C., Briese, V. & Richter, D.-U. A retrospective in vitro study of the impact of anti-diabetics and cardioselective pharmaceuticals on breast cancer. *Anticancer Res.* **32**, 2133–2138 (2012).

12. Hu, J. *et al.* β-Adrenergic Receptor Inhibitor and Oncolytic Herpesvirus Combination Therapy Shows Enhanced Antitumoral and Antiangiogenic Effects on Colorectal Cancer. *Front. Pharmacol.* **12**, 735278 (2021).

13. Coelho, M. *et al.* Antiproliferative effects of β-blockers on human colorectal cancer. *Oncol. Rep.* **33**, 2513–2520 (2015).

14. Sidorova, M. & Petrikaitė, V. The Effect of Beta Adrenoreceptor Blockers on Viability and Cell Colony Formation of Non-Small Cell Lung Cancer Cell Lines A549 and H1299. *Molecules* **27**, (2022).

15. Niu, M. *et al.* FBXL2 counteracts Grp94 to destabilize EGFR and inhibit EGFR-driven NSCLC growth. *Nat. Commun.* **12**, 5919 (2021).

16. Chaudhary, K. R. *et al.* Effects of β-Adrenergic Antagonists on Chemoradiation Therapy for Locally Advanced Non-Small Cell Lung Cancer. *J. Clin. Med.* **8**, (2019).

17. Nilsson, M. B. *et al.* Stress hormones promote EGFR inhibitor resistance in NSCLC: Implications for combinations with β-blockers. *Sci. Transl. Med.* **9**, (2017).

18. Chang, A. *et al.* Prevention of skin carcinogenesis by the β-blocker carvedilol. *Cancer Prev. Res. (Phila).* **8**, 27–36 (2015).

19. Deng, G.-H. *et al.* Exogenous norepinephrine attenuates the efficacy of sunitinib in a mouse cancer model. *J. Exp. Clin. Cancer Res.* **33**, 21 (2014).

20. Farhoumand, L. S. *et al.* The Adrenergic Receptor Antagonist Carvedilol Elicits Anti-Tumor Responses in Uveal Melanoma 3D Tumor Spheroids and May Serve as Co-Adjuvant Therapy with Radiation. *Cancers (Basel).* **14**, (2022).

21. Bustamante, P. *et al.* Beta-blockers exert potent anti-tumor effects in cutaneous and uveal melanoma. *Cancer Med.* **8**, 7265–7277 (2019).

22. Maccari, S. *et al.* Biphasic effects of propranolol on tumour growth in B16F10 melanoma-bearing mice. *Br. J. Pharmacol.* **174**, 139–149 (2017).

23. Zhou, C. *et al.* Propranolol induced G0/G1/S phase arrest and apoptosis in melanoma via AKT/MAPK pathway. *Oncotarget* **7**, 68314–68327 (2016).

24. Palm, D. *et al.* The norepinephrine-driven metastasis development of PC-3 human prostate cancer in BALB/c nude mice is inhibited by beta-blockers. *Int. J. cancer* **118**, 2744–2749 (2006).

25. Rasha, F. *et al.* Renin angiotensin system inhibition attenuates adipocyte-breast cancer cell interactions. *Exp. Cell Res.* **394**, 112114 (2020).

26. Namazi, S. *et al.* The role of captopril and losartan in prevention and regression of tamoxifen-induced resistance of breast cancer cell line MCF-7: An in vitro study. *Biomed. Pharmacother.* **68**, 565–571 (2014).

27. Napoleone, E. *et al.* Inhibition of the renin-angiotensin system downregulates tissue factor and vascular endothelial growth factor in human breast carcinoma. *Thromb. Res.* **129**, 736–742 (2012).

28. Brown, R. E., Lun, M., Prichard, J. W., Blasick, T. M. & Zhang, P. L. Morphoproteomic and pharmacoproteomic correlates in hormone-receptor-negative breast carcinoma cell lines. *Ann. Clin. Lab. Sci.* **34**, 251–262 (2004).

29. Yang, Y. *et al.* Enalapril overcomes chemoresistance and potentiates antitumor efficacy of 5-FU in colorectal cancer by suppressing proliferation, angiogenesis, and NF-κB/STAT3-regulated proteins. *Cell Death Dis.* **11**, 477 (2020).

30. Lu, Y. *et al.* S-Nitrosocaptopril prevents cancer metastasis in vivo by creating the hostile bloodstream microenvironment against circulating tumor. *Pharmacol. Res.* **139**, 535–549 (2019).

31. Lu, Y. *et al.* Nitric oxide inhibits hetero-adhesion of cancer to endothelial: restraining circulating tumor from initiating metastatic cascade. *Sci. Rep.* **4**, 4344 (2014).

32. Ni, S. *et al.* Discovery of candesartan cilexetic as a novel neddylation inhibitor for suppressing tumor growth. *Eur. J. Med. Chem.* **185**, 111848 (2020).

33. Redondo-Müller, M. A., Stevanovic-Walker, M., Barker, S., Puddefoot, J. R. & Vinson, G. P. Anti-cancer actions of a recombinant antibody (R6313/G2) against the angiotensin II AT1 receptor. *Endocr. Relat. Cancer* **15**, 277–288 (2008).

34. Du, N. *et al.* Angiotensin II receptor type 1 blockers suppress the cell proliferation effects of angiotensin II in breast cancer by inhibiting AT1R signaling. *Oncol. Rep.* **27**, 1893–1903 (2012).

35. Asgharzadeh, F. *et al.* Inhibition of angiotensin pathway via valsartan reduces tumor growth in models of colorectal cancer. *Toxicol. Appl. Pharmacol.* **440**, 115951 (2022).

36. Tabatabai, E. *et al.* Inhibition of angiotensin II type 1 receptor by candesartan reduces tumor growth and ameliorates fibrosis in colorectal cancer. *EXCLI J.* **20**, 863–878 (2021).

37. Hashemzehi, M. *et al.* Angiotensin receptor blocker Losartan inhibits tumor growth of colorectal cancer. *EXCLI J.* **20**, 506–521 (2021).

38. Martínez, V. R. *et al.* Interaction of Zn with Losartan. Activation of Intrinsic Apoptotic Signaling Pathway in Lung Cancer and Effects on Alkaline and Acid Phosphatases. *Biol. Trace Elem. Res.* **186**, 413–429 (2018).

39. Rasheduzzaman, M., Jeong, J.-K. & Park, S.-Y. Resveratrol sensitizes lung cancer cell to TRAIL by p53 independent and suppression of Akt/NF-κB signaling. *Life Sci.* **208**, 208–220 (2018).

40. Olschewski, D. N. *et al.* The Angiotensin II Type 1 Receptor Antagonist Losartan Affects NHE1-Dependent Melanoma Cell Behavior. *Cell. Physiol. Biochem. Int. J. Exp. Cell. Physiol. Biochem. Pharmacol.* **45**, 2560–2576 (2018).

41. Woo, Y. & Jung, Y. Angiotensin II receptor blockers induce autophagy in prostate cancer. *Oncol Lett* **13**, 3579–3585 (2017).

42. Islas, M. S. *et al.* Experimental and DFT characterization, antioxidant and anticancer activities of a Cu(II)-irbesartan complex: structure-antihypertensive activity relationships in Cu(II)-sartan complexes. *J. Biol. Inorg. Chem. JBIC a Publ. Soc. Biol. Inorg. Chem.* **21**, 851–863 (2016).

43. Alhusban, A. *et al.* Clinically relevant doses of candesartan inhibit growth of prostate tumor xenografts in vivo through modulation of tumor angiogenesis. *J. Pharmacol. Exp. Ther.* **350**, 635–645 (2014).

44. Da, Y. *et al.* Synthesis and biological evaluation of new fluorine substituted derivatives as angiotensin II receptor antagonists with anti-hypertension and anti-tumor effects. *Bioorg. Med. Chem.* **20**, 7101–7111 (2012).

45. Teranishi, J. *et al.* Evaluation of role of angiotensin III and aminopeptidases in prostate cancer . *Prostate* **68**, 1666–1673 (2008).

46. Ishiguro, H., Ishiguro, Y., Kubota, Y. & Uemura, H. Regulation of prostate cancer cell growth and PSA expression by angiotensin II receptor blocker with peroxisome proliferator-activated receptor gamma ligand like action. *Prostate* **67**, 924–932 (2007).

47. Uemura, H. *et al.* Antiproliferative activity of angiotensin II receptor blocker through cross-talk between stromal and epithelial prostate cancer. *Mol. Cancer Ther.* **4**, 1699–1709 (2005).

48. Dovio, A. *et al.* Differential expression of determinants of glucocorticoid sensitivity in androgen-dependent and androgen-independent human prostate cancer cell lines. *J. Steroid Biochem. Mol. Biol.* **116**, 29–36 (2009).

49. Leung, W.-H. *et al.* Modulation of NKG2D ligand expression and metastasis in tumors by spironolactone via RXRγ activation. *J. Exp. Med.* **210**, 2675–2692 (2013).

50. Sanomachi, T. *et al.* Spironolactone, a Classic Potassium-Sparing Diuretic, Reduces Survivin Expression and Chemosensitizes Cancer to Non-DNA-Damaging Anticancer Drugs. *Cancers (Basel).* **11**, (2019).

51. Sayedyahossein, S. *et al.* Pannexin 1 binds β-catenin to modulate melanoma cell growth and metabolism. *J. Biol. Chem.* **296**, 100478 (2021).

52. Papadopoli, D. *et al.* Perturbations of cancer cell metabolism by the antidiabetic drug canagliflozin. *Neoplasia* **23**, 391–399 (2021).

53. Eliaa, S. G., Al-Karmalawy, A. A., Saleh, R. M. & Elshal, M. F. Empagliflozin and Doxorubicin Synergistically Inhibit the Survival of Triple-Negative Breast Cancer via Interfering with the mTOR Pathway and Inhibition of Calmodulin: In Vitro and Molecular Docking Studies. *ACS Pharmacol. Transl. Sci.* **3**, 1330–1338 (2020).

54. Quagliariello, V. *et al.* NLRP3 as Putative Marker of Ipilimumab-Induced Cardiotoxicity in the Presence of Hyperglycemia in Estrogen-Responsive and Triple-Negative Breast Cancer. *Int. J. Mol. Sci.* **21**, (2020).

55. Zhou, J. *et al.* Sodium-glucose co-transporter-2 (SGLT-2) inhibition reduces glucose uptake to induce breast cancer cell growth arrest through AMPK/mTOR pathway. *Biomed. Pharmacother.* **132**, 110821 (2020).

56. Komatsu, S. *et al.* SGLT2 inhibitor ipragliflozin attenuates breast cancer cell proliferation. *Endocr. J.* **67**, 99–106 (2020).

57. Yamamoto, L. *et al.* Sodium-glucose cotransporter 2 inhibitor canagliflozin attenuates lung cancer cell proliferation in vitro. *Diabetol. Int.* **12**, 389–398 (2021).
