# Supplementary material for: Heart failure pharmacotherapy and cancer: pathways and pre-clinical/clinical evidence
Source: Eur Heart J. 2024 Mar 5;45(14):1224–40. doi: 10.1093/eurheartj/ehae105 (PMC11023004; doi:10.1093/eurheartj/ehae105)
Supplement: ehae105_Supplementary_Data [file ehae105_supplementary_data.zip › Supplementary_table_4_20231211.docx]

| **Effects of BBs on cancer assessed by meta-analyses of clinical studies** | | | | | | | | | | | |
| --- | --- | --- | --- | --- | --- | --- | --- | --- | --- | --- | --- |
| **ID** | **PRISMA or MOOSE guidelines** | **Baseline cancer status** | **Main aim** | **Number of included studies (study type)** | **Main outcomes** | **Outcomes by cancer types (number of studies)** | | | | | |
|  |  |  |  |  |  | **Breast cancer** | **Lung cancer** | **Colorectal cancer** | **Melanoma** | **Prostate** | **Other cancers** |
| **M. Monami et al. (2013)**[1] | Followed | Cancer-free patients | To investigate the relationship between BB treatment and the incidence of cancer in diabetic and non-diabetic patients. | 9 (RCT) | **BB use is associated with non-significant trend toward lower risk of cancer.** | Outcome was not broken down to cancer types | | | | | |
| **A. Yap et al. (2018)**[2] | Followed | Cancer patients | To investigate the association between BB use and cancer recurrence (CR), disease-free survival (DFS), and overall survival (OS). | 27 (observational) | BB use has no effect on CR, and has mixed effects on different cancer types. | **No effect** (3) | **No effect** (3) | **No effect** (3) | **Improved  DFS and OS** (1) | **Worsened OS** (2) | **Ovarian: no effect** (6) **Endometrial:** **no effect on DFS**, **worsened OS** (1) **Head & Neck: no effect on DFS**, **worsened OS** (1) **Renal:** **no effect** (1) **Esophagus: no effect** (1) **Pancreatic:** **no effect** (2) **Stomach:** **no effect** (1) |
| **Z. Na et al. (2018)**[3] | Followed | Cancer patients | To investigate the relationship between BB exposure and survival outcomes of various cancers. | 36 (observational and RCT) | BB use is associated with **significant increase of CSS**, but has no effect on OS, ACM, DFS, PFS, RFS. | **No effect** (6) | **No effect** (7) | **No effect** (2) | **Improved  OS** (2) | N/A | **Ovarian: improved OS** (5) **Endometrial:** N/A **Head & Neck:** N/A **Renal:** N/A **Esophagus:** N/A **Pancreatic:** **Improved OS** (2) **Stomach:** N/A |
| **C. Choi et al. (2014)**[4] | Followed | Cancer patients | To assess whether adding beta blockers to the treatment regimen of patients with various types of cancer had an impact on survival. | 18 (observational) | BB use is associated with **significant increase of OS and DFS.** | Outcome was not broken down to cancer types | | | | | |
| **J. Weberpals et al. (2016)**[5] | Followed | Cancer patients | To summarize evidence on the association between pre- and post-diagnostic beta blocker exposure and cancer survival. | 30 (observational) | BB use is associated with **significant increase of OS and CSS,** mainly driven by post-diagnostic BB use. No associations between selectivity of BB and OS or CSS. | **No effect** (6) | **No effect** (3) | **No effect** (5) | **Improved  OS** (2) | **No effect** (6) | **Ovarian: no effect** (2) **Endometrial:** N/A **Head & Neck:** N/A **Renal:** **no effect** (1) **Esophagus: no effect** (1) **Pancreatic:** **no effect** (2) **Stomach:** **no effect** (1) |
| **S. Zhong et al. (2016)**[6] | Followed | Cancer patients | To assess the relationship between postdiagnostic and prediagnostic β-blocker use and the survival of cancer patients for both all-cause mortality and cancer-specific mortality. | 24 (observational) | Prediagnostic BB use showed no benefit, but **postdiagnostic BB use improved all-cause mortality or cancer specific mortality**. | **Improved all cause mortality** (3) | **No effect** (4) | **No effect** (2) | **No effect**  (4) | **No effect** (2) | **Ovarian: improved all cause mortality** (1) **Endometrial:** N/A **Head & Neck:** N/A **Renal:** N/A **Esophagus:** N/A **Pancreatic:** N/A **Stomach:** N/A |
| **S. Riamondi et al. (2016)**[7] | Followed | Cancer patients | To shed light on possible role of the use of antihypertensive drugs in breast cancer development and survival. | 10 (observational) | BB use is associated with **significant improvement in OS and CSS**, and borderline improvement in DFS. | **Improved  OS and CSS** (8) | N/A | N/A | N/A | N/A | **Ovarian:** N/A **Endometrial:** N/A **Head & Neck:** N/A **Renal:** N/A **Esophagus:** N/A **Pancreatic:** N/A **Stomach:** N/A |
| **Z. Y. Wen et al. (2021)**[8] | Followed | Cancer patients | To further identify the correlation between post-diagnostic BB usage and ovarian cancer prognosis. | 11 (observational) | Post-diagnostic BB use is not associated with ovarian cancer prognosis. | N/A | N/A | N/A | N/A | N/A | **Ovarian: no effect** (11) **Endometrial:** N/A **Head & Neck:** N/A **Renal:** N/A **Esophagus:** N/A **Pancreatic:** N/A **Stomach:** N/A |
| **Z. Lei et al. (2021)**[9] | Followed | Cancer patients | To provide a systematic evaluation of the association between BB use and survival of lung cancer. | 10 (observational) | BB use was not associated with significantly affected OS in lung cancer. | N/A | **Improved OS in Stage III**  (4) **Improved OS when no surgery was performed** (2) **nsBBs significantly worsened OS** (4) | N/A | N/A | N/A | **Ovarian:** N/A **Endometrial:** N/A **Head & Neck:** N/A **Renal:** N/A **Esophagus:** N/A **Pancreatic:** N/A **Stomach:** N/A |
| **A. Majidi et al. (2020)**[10] | Not followed | Cancer patients | To review the evidence for a possible relation between common chronic disease medications and survival among women with ovarian cancer | 11 (observational) | **BB use has no effect on OS of women with ovarian cancer.** | N/A | N/A | N/A | N/A | N/A | **Ovarian: No effect** (11) **Endometrial:** N/A **Head & Neck:** N/A **Renal:** N/A **Esophagus:** N/A **Pancreatic:** N/A **Stomach:** N/A |
| **H. Tang et al. (2018)**[11] | Not followed | Cancer-free patients | To quantify the association between use of antihypertensive drugs and malignant melanoma risk | 8 (observational | **BB significantly increases risk of melanoma.** | N/A | N/A | N/A | **Increased risk of cancer**  (n=3) | N/A | **Ovarian:** No effect (11) **Endometrial:** N/A **Head & Neck:** N/A **Renal:** N/A **Esophagus:** N/A **Pancreatic:** N/A **Stomach:** N/A |
| **W. K. Childrens et al. (2015)**[12] | Not followed | Cancer patients | To perform a systematic review and meta-analysis of the effect of β-blockers on breast cancer outcomes | 7 (observational) | **BB use has no effect on breast CR or all cause mortality, but significantly decreases cancer death.** | **No effect on CR** (n=5) **significantly decreased cancer death** (n=4) **No effect on all cause mortality** (n=4) | N/A | N/A | N/A | N/A | **Ovarian:** N/A **Endometrial:** N/A **Head & Neck:** N/A **Renal:** N/A **Esophagus:** N/A **Pancreatic:** N/A **Stomach:** N/A |
| **M. Thiele et al. (2015)**[13] | Followed | Cancer-free patients | To evaluate the effect of nsBB on HCC. | 12 (RCT) | **nsBB use decrease risk of HCC.** | N/A | N/A | N/A | N/A | N/A | **Ovarian:** N/A **Endometrial:** N/A **Head & Neck:** N/A **Renal:** N/A **Esophagus:** N/A **Pancreatic:** N/A **Stomach:** N/A |
| **S. Gandini et al. (2018)**[14] | Followed | Cancer-free patients | To investigate the association between use of anti-hypertensive drugs and the risk of cutaneous melanoma and non-melanoma skin cancer | 19 (observational) | **BB significantly increases risk of skin cancer.** | N/A | N/A | N/A | **Increased risk of cancer**  (n=4) | N/A | **Ovarian:** N/A **Endometrial:** N/A **Head & Neck:** N/A **Renal:** N/A **Esophagus:** N/A **Pancreatic:** N/A **Stomach:** N/A **Hepatocellular:** N/A |
| **S. Bangalore et al. (2011)**[15] | Followed | Cancer-free patients | To assess the association between antihypertensive drugs and the risk of cancer in a comprehensive analysis of data from randomised clinical trials. | 70 (RCT) | **BB has no effect on risk of cancer or cancer mortality.** | Outcome was not broken down to cancer types | | | | | |
| **Y. Xie et al. (2019)**[16] | Followed | Cancer-free patients | To investigate how the use of antihypertensive medications may influence the incidence of bladder/kidney cancer | 31 (observational) | **BB significantly increases kidney cancer risk.** | N/A | N/A | N/A | N/A | N/A | **Ovarian:** N/A **Endometrial:** N/A **Head & Neck:** N/A **Renal:** **increased risk** (n=12) **Esophagus:** N/A **Pancreatic:** N/A **Stomach:** N/A **Hepatocellular:** N/A |
| **E. Copland et al. (2021)**[17] | Followed | Cancer-free patients | To investigate the association between antihypertensive medication and cancer in a large individual patient data meta-analysis of randomised clinical trials | 33 (RCT) | **BB use has no effect on risk of cancer or cancer mortality.** | **No effect on cacer risk** (n=4) | **No effect on cacer risk** (n=4) | **No effect on cancer risk** (n=3) | N/A | **No effect on cacer risk** (n=3) | **Ovarian:** N/A **Endometrial:** N/A **Head & Neck:** N/A **Renal: N/A** **Esophagus:** N/A **Pancreatic:** N/A **Stomach:** N/A **Hepatocellular:** N/A |
| **J. Qi et al. (2022)**[18] | Followed | Cancer-free patients | To investigate associations associations between colorectal cancer risk and antihypertensive medications: angiotensin-converting enzyme inhibitors (ACEIs), angiotensin II receptor blockers (ARBs), beta-blockers (BBs), calcium channel blockers (CCBs), and diuretics. | 13 (observational) | **BB use has no effect on colorectal cacer risk.** | N/A | N/A | **No effect on cancer risk** (n=4) | N/A | N/A | **Ovarian:** N/A **Endometrial:** N/A **Head & Neck:** N/A **Renal:** N/A **Esophagus:** N/A **Pancreatic:** N/A **Stomach:** N/A **Hepatocellular:** N/A |

| **Effects of RAASIs (ACEIs, ARBs, MRAs) on cancer assessed by meta-analyses of clinical studies** | | | | | | | | | | | |
| --- | --- | --- | --- | --- | --- | --- | --- | --- | --- | --- | --- |
| **ID** | **PRISMA or MOOSE guidelines** | **Baseline cancer status** | **Main aim** | **number of included studies (study type)** | **Main outcomes** | **Outcomes by cancer types (number of studies)** | | | | | |
|  |  |  |  |  |  | **Breast cancer** | **Lung cancer** | **Colorectal cancer** | **Melanoma** | **Prostate** | **Other cancers** |
| **C. I. Coleman et al. (2008)**[19] | Not followed | Cancer-free patients | To determine the association between commonly used antihypertensive agents and the incidence of cancer. | 27 (RCT) | Neither ARBs, nor ACEis had significant effects on incidence of cancer. | Outcome was not broken down to cancer types | | | | | |
| **I. Sipahi et al. (2010)**[20] | Not followed | Cancer-free patients | To examine the effect of ARBs on occurrence of new cancers. | 9 (RCT) | **ARBs significantly increased risk of new cancer compared to placebo. ARBs significantly increased the occurrence of new cancer in patients with ACEi background therapy, compared to ACEi alone.** No significant effect on cancer death. | No effect  (5) | **Increased risk** (5) | N/A | N/A | No effect  (5) | **Ovarian:** N/A **Endometrial:** N/A **Head & Neck:** N/A **Renal:** N/A **Esophagus:** N/A **Pancreatic:** N/A **Stomach:** N/A **Hepatocellular:** N/A |
| **S. Bangalore et al. (2011)**[15] | Followed | Cancer-free patients | To assess the association between antihypertensive drugs and the risk of cancer in randomised clinical trials. | 70 (RCT) | ARBs or ACEis had no significant effect on cancer risk, or cancer related deaths, compared to placebo. **The combination of ARBs and ACEis significantly increased cancer risk,** but not cancer deaths. | Outcome was not broken down to cancer types | | | | | |
| **I. Sipahi et al. (2011)**[21] | Followed | Cancer-free patients | To determine the effect of ACE inhibitors on cancer occurrence and cancer death, and on occurrence of gastrointestinal cancers given previous concerns of increased risk. | 14 (RCT) | ACEis have no significant effect on cancer risk, cancer deaths, or gastrointestinal cancers. | No effect  (14) | N/A | N/A | N/A | N/A | **Ovarian:** N/A **Endometrial:** N/A **Head & Neck:** N/A **Renal:** N/A **Esophagus:** N/A **Pancreatic:** N/A **Stomach:** N/A **Hepatocellular:** N/A |
| **C. Yoon et al. (2011)**[22] | Not followed | Cancer-free patients | To assess the association between use of ACEis or ARBs and cancer risk. | 28 (observational) | No significant association between the use of ACEis or ARBs and the risk of cancer in all of the studies. **Beneficial effect of ACEis or ARBs on risk of cancer in Cohort studies, Nested case-control studies, and studies with long-term follow-up.** **ACEis or ARBs marginally increased the risk of cancer in Conventional case-control studies.** | No effect  (6, 8) | No effect  (4, 3) | No effect  (4, 2) | **Increased risk** (3, 1) | No effect  (8, 5) | **Ovarian:** N/A **Endometrial:** N/A **Head & Neck:** N/A **Renal:** **increased risk** (6, 2) **Esophagus: decreased risk** (2, 2) **Pancreatic:** N/A **Stomach:** no effect (2, 2) **Hepatocellular:** N/A |
| **Y. Dai et al. (2015)**[23] | Followed | Both cancer-free and cancer patients | To investigate the association between ACEi or ARB therapy and colorectal cancer. | 11 (observational) | ACEi or ARB use **significantly decreases colorectal cancer incidence**, but have no significant effect on CSS. No significant dose-reponse relationship was found. | N/A | N/A | **Decreased incidence**  (6) **No effect on CSS** (3) | N/A | N/A | **Ovarian:** N/A **Endometrial:** N/A **Head & Neck:** N/A **Renal:** N/A **Esophagus:** N/A **Pancreatic:** N/A **Stomach:** N/A **Hepatocellular:** N/A |
| **Y. Mao et al. (2016)**[24] | Followed | Cancer-free patients | To summarize and to quantity the existing evidence on the relationship between RAS inhibitors and prostate cancer based on all relevant cohort studies. | 9 (observational) | ACEi or ARB use **significantly decreases risk for prostate cancer**. | N/A | N/A | N/A | N/A | **Decreased incidence**  (6) | **Ovarian:** N/A **Endometrial:** N/A **Head & Neck:** N/A **Renal:** N/A **Esophagus:** N/A **Pancreatic:** N/A **Stomach:** N/A **Hepatocellular:** N/A |
| **J. Shen et al. (2016)**[25] | Followed | Both cancer-free and cancer patients | To assess the association between ACEI/ARB use and risk of cancer and death. | 31 (17 observational, 14 RCT) | **ACEI/ARB users had a lower incidence of cancer in the observational studies but not in the RCTs  Mortality reduction with ARB/ACEI was marginally signifcant in the observational studies but not in the RCTs  Incidence reduction was not significantly different with the duration of the follow-up** | **No effect on incidence** (n=5 observational) **No effect on incidence** (n=8 RCT) | **Decreased incidence** (n=6 observational) **No effect on incidence** (n=9 RCT) | **No effect on incidence**  (n=5 observational) | N/A | **No effect on incidence** (n=5 observational) **No effect on incidence** (n=9 RCT) | **Ovarian:** N/A **Endometrial:** N/A **Head & Neck:** N/A **Renal:** N/A **Esophagus:** N/A **Pancreatic:** N/A **Stomach:** N/A **Hepatocellular:** N/A |
| **T. Song et al. (2017)**[26] | Followed | Cancer patients | To assess the current evidence on the potential benefit of angiotensin-converting enzyme inhibitors (ACEIs) or angiotensin receptor blockers (ARBs) on cancer recurrence and survival. | 11 (observational) | **The use of ACEIs or ARBs in cancer patients can lead to a 40 and 25% reduction in the risk of cancer recurrence and mortality.** | Outcome was not broken down to cancer types | | | | | |
| **Y. Zhao et al. (2016)**[27] | Not followed | Cancer patients | To investigate the risk of cancer associated with ARB at different background ACEI levels. | 17 (RCT) | **No significant differences in cancer incidence when compared ARB alone with placebo alone, ARB alone with ACEI alone, ARB plus partial use of ACEI with placebo plus partial use of ACEI, or ARB plus ACEI combination with ACEI** | Outcome was not broken down to cancer types | | | | | |
| **H. Sun et al. (2017)**[28] | Not followed | Cancer patients | To evaluate the effect of RAS inhibitors on recurrence, metastasis, and survival in cancer patients. | 55 (observational) | **Mixed effect on OS depending on cancer types. Overall OS, DFS and PFS show significant improvement mainly driven by ARBs, but not ACEis** | No effect on OS (n=7) | **No effect on OS** (n=7) | **No effect on OS** (n=5) | **No effect on OS** (n=1) | **No effect on OS** (n=2) | **Ovarian:** N/A **Endometrial:** N/A **Head & Neck: no effect on OS** (n=1) **Renal:** **improved OS** (n=7) **Esophagus: no effect on OS** (n=2) **Pancreatic:** **improved OS** (n=2) **Stomach:** **improved OS** (n=2) **Hepatocellular:** **improved OS** (n=2) |
| **S. Gandini et al. (2018)**[14] | Followed | Cancer-free patients | To investigate the association between use of anti-hypertensive drugs and the risk of cutaneous melanoma and non-melanoma skin cancer. | 19 (observational) | **ACEi and ARB have no effect on risk of skin cancer, including melanoma** | N/A | N/A | N/A | **No effect on risk of cancer** (n=5 for ACEi, n=3 for ARB) | N/A | **Ovarian:** N/A **Endometrial:** N/A **Head & Neck:** N/A **Renal:** N/A **Esophagus:** N/A **Pancreatic:** N/A **Stomach:** N/A **Hepatocellular:** N/A |
| **L. Cao et al. (2018)**[29] | Followed | Cancer-free patients | To investigate the relationship between antihypertensive drugs use and the risk of prostate cancer. | 21 (observational) | **ACEi and ARB have no effect on risk of prostate cancer neither in cohort, nor in case control studies** | N/A | N/A | N/A | N/A | **No effect on risk of cancer** (n=10 for ACEi, n=5 for ARB) | **Ovarian:** N/A **Endometrial:** N/A **Head & Neck:** N/A **Renal:** N/A **Esophagus:** N/A **Pancreatic:** N/A **Stomach:** N/A **Hepatocellular:** N/A |
| **T. Datzmann et al. (2019)**[30] | Followed | Cancer-free patients | To investigate the available randomised and observational study data on ARBs and carcinogenesis. | 12 (7 RCT, 5 observational) | **Risk of cancer was elevated in case-constrol studies**, **but not in RCTs or in cohort studies. No effect on tumour specific mortality.** | **No effect on tumor specific mortality** (n=6) | **No effect on tumor specific mortality** (n=5) | N/A | N/A | **No effect on tumor specific mortality** (n=3) | **Ovarian:** N/A **Endometrial:** N/A **Head & Neck:** N/A **Renal:** N/A **Esophagus:** N/A **Pancreatic:** N/A **Stomach:** N/A **Hepatocellular:** N/A |
| **Q. Zhou et al. (2020)**[31] | Followed | Cancer patients | To investigate the effect of long-term oral RAS-blockade (ACEi or ARB) on digestive system malignancies. | 13 (observational) | **RAS blockade improves CSS, OS and RFS of digestive system malignancies, which is mainly driven by ARBs** | N/A | N/A | **Improved OS** (n=5) | N/A | N/A | **Ovarian:** N/A **Endometrial:** N/A **Head & Neck:** N/A **Renal:** N/A **Esophagus:** N/A **Pancreatic: improved OS** (n=3) **Stomach:** N/A **Hepatocellular:** **improved OS** (n=2) |
| **X. Chen et al. (2020)**[32] | Not followed | Cancer patients | To determine whether use of the renin–angiotensin system (RAS) inhibitors would increase colorectal cancer morbidity and mortality. | 16 (observational) | **RAS blockade improves risk of colorectal cancer and CSS** | N/A | N/A | **Improved risk of cancer** (n=16) **Improved** **CSS** (n=3) | N/A | N/A | **Ovarian:** N/A **Endometrial:** N/A **Head & Neck:** N/A **Renal:** N/A **Esophagus:** N/A **Pancreatic:** N/A **Stomach:** N/A **Hepatocellular:** N/A |
| **F. Asgharzadeh et al. (2020)**[33] | Not followed | Cancer patients | To explore the potential clinical impact of ACEI/ARB in renal cancer. | 9  (observational) | **RAS blockade improves RCC mortality, mainly driven by ARBs.** | N/A | N/A | N/A | N/A | N/A | **Ovarian:** N/A **Endometrial:** N/A **Head & Neck:** N/A **Renal:** **improved mortality** (n=3) **Esophagus:** N/A **Pancreatic:** N/A **Stomach:** N/A **Hepatocellular:** N/A |
| **Y. Xie et al. (2019)**[16] | Followed | Cancer-free patients | To investigate how the use of antihypertensive medications may influence the incidence of bladder/kidney cancer | 31 (observational) | **ARB and ACEi significantly increases kidney cancer risk. ARB siggnificantly increase bladder cancer risk.** | N/A | N/A | N/A | N/A | N/A | **Ovarian:** N/A **Endometrial:** N/A **Head & Neck:** N/A **Renal:** **increased risk** (n=4 for ARB, n=10 for ACEi) **Esophagus:** N/A **Pancreatic:** N/A **Stomach:** N/A **Hepatocellular:** N/A |
| **M. Batais et al. (2021)**[34] | Followed | Cancer-free patients | To investigate relationship between the use of angiotensin converting enzyme inhibitors (ACEIs) and the risk of lung cancer | 13 (1 RCT, 12 observational) | **ACEI use has no effect on lung cancer risk.** | N/A | **No effect on cancer risk** (n=13) | N/A | N/A | N/A | **Ovarian:** N/A **Endometrial:** N/A **Head & Neck:** N/A **Renal:** N/A **Esophagus:** N/A **Pancreatic:** N/A **Stomach:** N/A **Hepatocellular:** N/A |
| **E. Copland et al. (2021)**[17] | Possibly followed | Cancer-free patients | To investigate the association between antihypertensive medication and cancer in a large individual patient data meta-analysis of randomised clinical trials. | 33 (RCT) | **ARB or ACEI use have no effect on cacer risk or cancer mortality.** | **No effect on cacer risk for ARB** (n=10) **or ACEI** (n=11) | **No effect on cacer risk for ARB** (n=12) **or ACEI** (n=13) | **No effect on cacer risk for ARB** (n=12) **or ACEI** (n=11) | N/A | **No effect on cacer risk for ARB** (n=12) **or ACEI** (n=11) | **Ovarian:** N/A **Endometrial:** N/A **Head & Neck:** N/A **Renal:** N/A **Esophagus:** N/A **Pancreatic:** N/A **Stomach:** N/A **Hepatocellular:** N/A |
| **J. Qi et al. (2022)**[18] | Followed | Cancer-free patients | To investigate associations associations between colorectal cancer risk and antihypertensive medications: angiotensin-converting enzyme inhibitors (ACEIs), angiotensin II receptor blockers (ARBs), beta-blockers (BBs), calcium channel blockers (CCBs), and diuretics. | 13 (observational) | **ARB or ACEI use have no effect on colorectal cacer risk.** | N/A | N/A | No effect on cancer risk for ACEI (n=5) or ARB (n=4) | N/A | N/A | **Ovarian:** N/A **Endometrial:** N/A **Head & Neck:** N/A **Renal:** N/A **Esophagus:** N/A **Pancreatic:** N/A **Stomach:** N/A **Hepatocellular:** N/A |
| **H. Tang et al. (2018)**[11] | Not followed | Cancer-free patients | To quantify the association between use of antihypertensive drugs and malignant melanoma risk. | 8 (observational) | **ACEI or ARB has no effect on risk of melanoma** | N/A | N/A | N/A | **No effect risk of cancer**  **for ARB** (n=3) **or ACEI** (n=4) | N/A | **Ovarian: No effect** (n=11) **Endometrial:** N/A **Head & Neck:** N/A **Renal:** N/A **Esophagus:** N/A **Pancreatic:** N/A **Stomach:** N/A |
| **K. Bommareddy et al. (2022)**[35] | Followed | Cancer-free patients | To determine the pooled occurrence of cancers, in particular breast and prostate cancers, among those who were ever treated with spironolactone. | 7 (observational) | **Spironolacton use significantly decreased risk of prostate cancer**, but had no effect on other cancer types | No effect (3) | N/A | N/A | N/A | **Decreased risk of cancer** (n=4) | **Ovarian:** no effect (n=2) **Endometrial:** N/A **Head & Neck:** N/A **Renal:** no effect (n=3) **Esophagus:** no effect (n=2) **Pancreatic:** N/A **Stomach:** no effect (n=2) **Hepatocellular:** N/A |

| **Effects of SGLT2Is on cancer assessed by meta-analyses of clinical studies** | | | | | | | | | | | |
| --- | --- | --- | --- | --- | --- | --- | --- | --- | --- | --- | --- |
| **ID** | **PRISMA or MOOSE guidelines** | **Baseline cancer status** | **Main aim** | **number of included studies (study type)** | **Main outcomes** | **Outcomes by cancer types (number of studies)** | | | | | |
|  |  |  |  |  |  | **Breast cancer** | **Lung cancer** | **Colorectal cancer** | **Melanoma** | **Prostate** | **Other cancers** |
| **H. Cui et al. (2022)**[35] | Followed | Cancer-free patients | To assess the effects of antidiabetic medications on pancreatic cancer in patients with diabetes mellitus. | 47 (RCTs & Observational) | **Observational studies show no effect of metformin/TZD/SU/INS/DPP4i on prostate cancer. RCTs show significant decrease in prostatce cancer risk by TZDs/GLP-1RAs, but not with SGLT2is.** | N/A | N/A | N/A | N/A | **No effect** (n=7) | **Ovarian:** N/A **Endometrial:** N/A **Head & Neck:** N/A **Renal:** N/A **Esophagus:** N/A **Pancreatic:** N/A **Stomach:** N/A **Hepatocellular:** N/A |
| **R. Benedetti et al. (2022)**[35] | Followed | Cancer-free patients | To assess the effects of SGLT2i on all cause cancer incidence in patients with hyperglycaemia. | 20 (RCTs) | **Overall reduced risk of cancer compared to placebo particularly by dapagliflozin and ertugliflozin. 2 big trials shift the overall effect size to benefitial effect of SGLT2is, overall big CIs (small studies) with no effect, EMPA shows increased risk in 3 big trials but had no effect on overall effect size** | Outcome was not broken down to cancer types | | | | | |
| **I. Dicembrini et al. (2019)**[36] | Followed | Cancer-free patients | To assess the effects of SGLT2i on all cause cancer incidence. | 27 (RCTs) | **No effect of SGLT2is on incidence of overall cancer** | **No effect** (n=15) | **No effect** (n=16) | N/A | N/A | **No effect** (n=16) | **Ovarian:** N/A **Endometrial:** N/A **Head & Neck:** N/A **Renal:** N**o effect** (n=7) **Esophagus:** N/A **Pancreatic:** N**o effect** (n=8) **Stomach:** N/A **Hepatocellular: No effect** (n=5) |
| **H. Tang et al. (2018)**[37] | Not followed | Cancer-free patients | To assess the effect of SGLT2i on skin cancer. | 21 (RCTs) | **Almost (but not significantly) increased melanoma risk, almost (but not significantly) decreased non-melanoma skin cancer risk** | N/A | N/A | N/A | **No effect (n=7)** | N/A | **Ovarian:** N/A **Endometrial:** N/A **Head & Neck:** N/A **Renal:** N/A **Esophagus:** N/A **Pancreatic:** N/A **Stomach:** N/A **Hepatocellular:** N/A |
| **H. Tang et al. (2017)**[38] | Followed | Cancer-free patients | To assess the effect of SGLT2i on all cause cancer risk in T2DM patients. | 46 (RCTs) | **Risk of bladder cancer might be increased with SGLT2 inhibitors, especially empagliflozin. Canagliflozin might be protective against gastrointestinal cancers.** | **No effect** | **No effect** | N/A | N/A | **No effect** | **Ovarian:** N/A **Endometrial:** N/A **Head & Neck:** N/A **Renal:** no effect **Esophagus:** N/A **Pancreatic:** N/A **Stomach:** N/A **Hepatocellular:** N/A |
| **N. Shi et al. (2021)**[39] | Followed | Cancer-free patients | To determine the relationship between SGLT-2i and malignancy risk in T2DM patients. | 84 (RCTs) | **DAPA significantly increased risk of overall cancer compared to other antidiabetic drugs. EMPA significantly increases risk of overall cancer compared to placebo (n=15). EMPA significantly increases digestive system malignancies compared to placebo (n=8).** | **No effect** (n=23) | **No effect** (n=19) | N/A | N/A | N/A | **Ovarian:** N/A **Endometrial:** N/A **Head & Neck:** N/A **Renal:** N/A **Esophagus:** N/A **Pancreatic:** N/A **Stomach:** N/A **Hepatocellular:** N/A |

**Supplementary table 4.:** Meta-analyses of observational studies or randomized controlled trials investigating the effect of guideline-directed HF pharmacotherapies on cancer.

**References**

1. Monami, M.; Filippi, L.; Ungar, A.; Sgrilli, F.; Antenore, A.; Dicembrini, I.; et al. Further Data on Beta-Blockers and Cancer Risk: Observational Study and Meta-Analysis of Randomized Clinical Trials. *Curr. Med. Res. Opin.* **2013**, *29*, 369–378, doi:10.1185/03007995.2013.772505.

2. Yap, A.; Lopez-Olivo, M.A.; Dubowitz, J.; Pratt, G.; Hiller, J.; Gottumukkala, V.; et al. Effect of Beta-Blockers on Cancer Recurrence and Survival: A Meta-Analysis of Epidemiological and Perioperative Studies. *Br. J. Anaesth.* **2018**, *121*, 45–57, doi:https://doi.org/10.1016/j.bja.2018.03.024.

3. Na, Z.; Qiao, X.; Hao, X.; Fan, L.; Xiao, Y.; Shao, Y.; et al. The Effects of Beta-Blocker Use on Cancer Prognosis: A Meta-Analysis Based on 319,006 Patients. *Onco. Targets. Ther.* **2018**, *11*, 4913–4944, doi:10.2147/OTT.S167422.

4. Choi, C.H.; Song, T.; Kim, T.H.; Choi, J.K.; Park, J.-Y.; Yoon, A.; et al. Meta-Analysis of the Effects of Beta Blocker on Survival Time in Cancer Patients. *J. Cancer Res. Clin. Oncol.* **2014**, *140*, 1179–1188, doi:10.1007/s00432-014-1658-7.

5. Weberpals, J.; Jansen, L.; Carr, P.R.; Hoffmeister, M.; Brenner, H. Beta Blockers and Cancer Prognosis – The Role of Immortal Time Bias: A Systematic Review and Meta-Analysis. *Cancer Treat. Rev.* **2016**, *47*, 1–11, doi:https://doi.org/10.1016/j.ctrv.2016.04.004.

6. Zhong, S.; Yu, D.; Zhang, X.; Chen, X.; Yang, S.; Tang, J.; et al. β-Blocker Use and Mortality in Cancer Patients: Systematic Review and Meta-Analysis of Observational Studies. *Eur. J. cancer Prev. Off. J. Eur. Cancer Prev. Organ.* **2016**, *25*, 440–448, doi:10.1097/CEJ.0000000000000192.

7. Raimondi, S.; Botteri, E.; Munzone, E.; Cipolla, C.; Rotmensz, N.; DeCensi, A.; et al. Use of Beta-Blockers, Angiotensin-Converting Enzyme Inhibitors and Angiotensin Receptor Blockers and Breast Cancer Survival: Systematic Review and Meta-Analysis. *Int. J. Cancer* **2016**, *139*, 212–219, doi:https://doi.org/10.1002/ijc.30062.

8. Wen, Z.-Y.; Gao, S.; Gong, T.-T.; Jiang, Y.-T.; Zhang, J.-Y.; Zhao, Y.-H.; et al. Post-Diagnostic Beta Blocker Use and Prognosis of Ovarian Cancer: A Systematic Review and Meta-Analysis of 11 Cohort Studies With 20,274 Patients . *Front. Oncol.*  2021, *11*.

9. Lei, Z.; Yang, W.; Zuo, Y. Beta-Blocker and Survival in Patients with Lung Cancer: A Meta-Analysis. *PLoS One* **2021**, *16*, e0245773.

10. Majidi, A.; Na, R.; Dixon-Suen, S.; Jordan, S.J.; Webb, P.M. Common Medications and Survival in Women with Ovarian Cancer: A Systematic Review and Meta-Analysis. *Gynecol. Oncol.* **2020**, *157*, 678–685, doi:https://doi.org/10.1016/j.ygyno.2020.03.028.

11. Tang, H.; Fu, S.; Zhai, S.; Song, Y.; Han, J. Use of Antihypertensive Drugs and Risk of Malignant Melanoma: A Meta-Analysis of Observational Studies. *Drug Saf.* **2018**, *41*, 161–169, doi:10.1007/s40264-017-0599-x.

12. Childers, W.K.; Hollenbeak, C.S.; Cheriyath, P. β-Blockers Reduce Breast Cancer Recurrence and Breast Cancer Death: A Meta-Analysis. *Clin. Breast Cancer* **2015**, *15*, 426–431, doi:10.1016/j.clbc.2015.07.001.

13. Thiele, M.; Albillos, A.; Abazi, R.; Wiest, R.; Gluud, L.L.; Krag, A. Non-Selective Beta-Blockers May Reduce Risk of Hepatocellular Carcinoma: A Meta-Analysis of Randomized Trials. *Liver Int.* **2015**, *35*, 2009–2016, doi:https://doi.org/10.1111/liv.12782.

14. Gandini, S.; Palli, D.; Spadola, G.; Bendinelli, B.; Cocorocchio, E.; Stanganelli, I.; et al. Anti-Hypertensive Drugs and Skin Cancer Risk: A Review of the Literature and Meta-Analysis. *Crit. Rev. Oncol. Hematol.* **2018**, *122*, 1–9, doi:https://doi.org/10.1016/j.critrevonc.2017.12.003.

15. Bangalore, S.; Kumar, S.; Kjeldsen, S.E.; Makani, H.; Grossman, E.; Wetterslev, J.; et al. Antihypertensive Drugs and Risk of Cancer: Network Meta-Analyses and Trial Sequential Analyses of 324 168 Participants from Randomised Trials. *Lancet Oncol.* **2011**, *12*, 65–82, doi:https://doi.org/10.1016/S1470-2045(10)70260-6.

16. Xie, Y.; Xu, P.; Wang, M.; Zheng, Y.; Tian, T.; Yang, S.; et al. Antihypertensive Medications Are Associated with the Risk of Kidney and Bladder Cancer: A Systematic Review and Meta-Analysis. *Aging (Albany. NY).* **2020**, *12*, 1545–1562, doi:10.18632/aging.102699.

17. Copland, E.; Canoy, D.; Nazarzadeh, M.; Bidel, Z.; Ramakrishnan, R.; Woodward, M.; et al. Antihypertensive Treatment and Risk of Cancer: An Individual Participant Data Meta-Analysis. *Lancet Oncol.* **2021**, *22*, 558–570, doi:10.1016/S1470-2045(21)00033-4.

18. Qi, J.; An, R.; Bhatti, P.; Spinelli, J.J.; Murphy, R.A. Anti-Hypertensive Medications and Risk of Colorectal Cancer: A Systematic Review and Meta-Analysis. *Cancer causes &amp; Control CCC* **2022**, *33*, 801–812, doi:10.1007/s10552-022-01570-1.

19. Coleman, C.I.; Baker, W.L.; Kluger, J.; White, C.M. Antihypertensive Medication and Their Impact on Cancer Incidence: A Mixed Treatment Comparison Meta-Analysis of Randomized Controlled Trials. *J. Hypertens.* **2008**, *26*.

20. Sipahi, I.; Debanne, S.M.; Rowland, D.Y.; Simon, D.I.; Fang, J.C. Angiotensin-Receptor Blockade and Risk of Cancer: Meta-Analysis of Randomised Controlled Trials. *Lancet Oncol.* **2010**, *11*, 627–636, doi:https://doi.org/10.1016/S1470-2045(10)70106-6.

21. Sipahi, I.; Chou, J.; Mishra, P.; Debanne, S.M.; Simon, D.I.; Fang, J.C. Meta-Analysis of Randomized Controlled Trials on Effect of Angiotensin-Converting Enzyme Inhibitors on Cancer Risk. *Am. J. Cardiol.* **2011**, *108*, 294–301, doi:https://doi.org/10.1016/j.amjcard.2011.03.038.

22. Yoon, C.; Yang, H.-S.; Jeon, I.; Chang, Y.; Park, S.M. Use of Angiotensin-Converting-Enzyme Inhibitors or Angiotensin-Receptor Blockers and Cancer Risk: A Meta-Analysis of Observational Studies. *CMAJ* **2011**, *183*, E1073-84, doi:10.1503/cmaj.101497.

23. Dai, Y.-N.; Wang, J.-H.; Zhu, J.-Z.; Lin, J.-Q.; Yu, C.-H.; Li, Y.-M. Angiotensin-Converting Enzyme Inhibitors/Angiotensin Receptor Blockers Therapy and Colorectal Cancer: A Systematic Review and Meta-Analysis. *Cancer Causes Control* **2015**, *26*, 1245–1255, doi:10.1007/s10552-015-0617-1.

24. Mao, Y.; Xu, X.; Wang, X.; Zheng, X.; Xie, L. Is Angiotensin-Converting Enzyme Inhibitors/Angiotensin Receptor Blockers Therapy Protective against Prostate Cancer? *Oncotarget* **2016**, *7*, 6765–6773, doi:10.18632/oncotarget.6837.

25. Shen, J.; Huang, Y.-M.; Wang, M.; Hong, X.-Z.; Song, X.-N.; Zou, X.; et al. Renin-Angiotensin System Blockade for the Risk of Cancer and Death. *J. Renin. Angiotensin. Aldosterone. Syst.* **2016**, *17*, doi:10.1177/1470320316656679.

26. Song, T.; Choi, C.H.; Kim, M.K.; Kim, M.-L.; Yun, B.S.; Seong, S.J. The Effect of Angiotensin System Inhibitors (Angiotensin-Converting Enzyme Inhibitors or Angiotensin Receptor Blockers) on Cancer Recurrence and Survival: A Meta-Analysis. *Eur. J. cancer Prev. Off. J. Eur. Cancer Prev. Organ.* **2017**, *26*, 78–85, doi:10.1097/CEJ.0000000000000269.

27. Zhao, Y.-T.; Li, P.-Y.; Zhang, J.-Q.; Wang, L.; Yi, Z. Angiotensin II Receptor Blockers and Cancer Risk: A Meta-Analysis of Randomized Controlled Trials. *Medicine (Baltimore).* **2016**, *95*, e3600, doi:10.1097/MD.0000000000003600.

28. Sun, H.; Li, T.; Zhuang, R.; Cai, W.; Zheng, Y. Do Renin–Angiotensin System Inhibitors Influence the Recurrence, Metastasis, and Survival in Cancer Patients? *Med.* **2017**, *96*, e6394, doi:10.1097/md.0000000000006394.

29. Cao, L.; Zhang, S.; Jia, C.-M.; He, W.; Wu, L.-T.; Li, Y.-Q.; et al. Antihypertensive Drugs Use and the Risk of Prostate Cancer: A Meta-Analysis of 21 Observational Studies. *BMC Urol.* **2018**, *18*, 17, doi:10.1186/s12894-018-0318-7.

30. Datzmann, T.; Fuchs, S.; Andree, D.; Hohenstein, B.; Schmitt, J.; Schindler, C. Systematic Review and Meta-Analysis of Randomised Controlled Clinical Trial Evidence Refutes Relationship between Pharmacotherapy with Angiotensin-Receptor Blockers and an Increased Risk of Cancer. *Eur. J. Intern. Med.* **2019**, *64*, 1–9, doi:10.1016/j.ejim.2019.04.019.

31. Zhou, Q.; Chen, D.-S.; Xin, L.; Zhou, L.-Q.; Zhang, H.-T.; Liu, L.; et al. The Renin-Angiotensin System Blockers and Survival in Digestive System Malignancies: A Systematic Review and Meta-Analysis. *Medicine (Baltimore).* **2020**, *99*, e19075, doi:10.1097/MD.0000000000019075.

32. Chen, X.; Yi, C.-H.; Ya, K.-G. Renin-Angiotensin System Inhibitor Use and Colorectal Cancer Risk and Mortality: A Dose-Response Meta Analysis. *J. Renin. Angiotensin. Aldosterone. Syst.* **2020**, *21*, 1470320319895646, doi:10.1177/1470320319895646.

33. Asgharzadeh, F.; Hashemzehi, M.; Moradi-Marjaneh, R.; Hassanian, S.M.; Ferns, G.A.; Khazaei, M.; et al. Angiotensin-Converting Enzyme Inhibitors and Angiotensin Receptor Blockers as Therapeutic Options in the Treatment of Renal Cancer: A Meta-Analysis. *Life Sci.* **2020**, *242*, 117181, doi:10.1016/j.lfs.2019.117181.

34. Batais, M.; Almigbal, T.; Alotaibi, K.; Alodhayani, A.; Alkhushail, A.; Altheaby, A.; et al. Angiotensin Converting Enzyme Inhibitors and Risk of Lung Cancer: A Systematic Review and Meta-Analysis. *Medicine (Baltimore).* **2021**, *100*, e25714, doi:10.1097/MD.0000000000025714.

35. Bommareddy, K.; Hamade, H.; Lopez-Olivo, M.A.; Wehner, M.; Tosh, T.; Barbieri, J.S. Association of Spironolactone Use With Risk of Cancer: A Systematic Review and Meta-Analysis. *JAMA dermatology* **2022**, *158*, 275–282, doi:10.1001/jamadermatol.2021.5866.

36. Dicembrini, I.; Nreu, B.; Mannucci, E.; Monami, M. Sodium-Glucose Co-Transporter-2 (SGLT-2) Inhibitors and Cancer: A Meta-Analysis of Randomized Controlled Trials. *Diabetes. Obes. Metab.* **2019**, *21*, 1871–1877, doi:10.1111/dom.13745.

37. Tang, H.; Yang, K.; Song, Y.; Han, J. Meta-Analysis of the Association between Sodium-Glucose Co-Transporter-2 Inhibitors and Risk of Skin Cancer among Patients with Type 2 Diabetes. *Diabetes. Obes. Metab.* **2018**, *20*, 2919–2924, doi:10.1111/dom.13474.

38. Tang, H.; Dai, Q.; Shi, W.; Zhai, S.; Song, Y.; Han, J. SGLT2 Inhibitors and Risk of Cancer in Type 2 Diabetes: A Systematic Review and Meta-Analysis of Randomised Controlled Trials. *Diabetologia* **2017**, *60*, 1862–1872, doi:10.1007/s00125-017-4370-8.

39. Shi, N.; Shi, Y.; Xu, J.; Si, Y.; Yang, T.; Zhang, M.; et al. SGLT-2i and Risk of Malignancy in Type 2 Diabetes: A Meta-Analysis of Randomized Controlled Trials . *Front. Public Heal.*  2021, *9*.
